# A computational pipeline for a neurotransmitter-centric analysis of the effects of psychiatric medication on EEG spectral power

**DOI:** 10.3389/fpsyt.2026.1737357

**Published:** 2026-06-19

**Authors:** Samar Samy Zekerallah, Anna Alexandra Maxion, Jana Zweerings, Paula Teucher, Klaus Mathiak, Ekaterina Kutafina, Arnim Johannes Gaebler

**Affiliations:** 1 Department of Psychiatry, Psychotherapy and Psychosomatics, Faculty of Medicine, RWTH Aachen University, Aachen, Germany; 2 Joint Research Center for Computational Biomedicine, Faculty of Medicine, RWTH Aachen University, Aachen, Germany; 3 Scientific Center for Neuropathic Pain Aachen, Faculty of Medicine, RWTH Aachen University, Aachen, Germany; 4 Center for Human Genetics and Genomic Medicine, Faculty of Medicine, RWTH Aachen University, Aachen, Germany; 5 Institute for Biomedical Informatics, Faculty of Medicine and University Hospital Cologne, University of Cologne, Cologne, Germany; 6 Institute for Neurophysiology, Faculty of Medicine, RWTH Aachen University, Aachen, Germany

**Keywords:** biomarkers, linear mixed-effects models, neurotransmitter systems, personalized psychiatry, pharmaco-EEG, psychotropic medications, receptor profiles, spectral power

## Abstract

**Introduction:**

Traditional pharmaco-electroencephalography (EEG) studies have mainly examined the effects of psychotropic medications at the level of individual drugs or broad drug classes, limiting biological specificity and clinical translation. This study aimed to determine whether modeling EEG spectral power changes according to the engagement of distinct neurotransmitter systems provides a more mechanistic understanding of psychotropic drug effects in a real-world clinical population.

**Methods:**

We analyzed 4,128 EEG sessions from 2,083 patients in the Temple University Hospital EEG Corpus, a large heterogeneous dataset. EEG data were preprocessed and segmented into canonical frequency bands (delta, theta, alpha, beta, and gamma). Psychotropic medication data were systematically extracted and coded at the receptor level for serotonin, dopamine, norepinephrine, histamine, and acetylcholine systems using the Neuroscience-based Nomenclature framework. Receptor profiles were summarized to represent each patient’s overall neurotransmitter engagement (agonistic, neutral, antagonistic, or mixed). Linear mixed-effects models were applied to assess relationships between neurotransmitter profiles and log-transformed spectral power while controlling for electrode location and patient-level variability.

**Results:**

Frequency- and region-specific EEG patterns were identified across neurotransmitter systems. Dopamine antagonists were associated with higher delta and theta power at central electrode locations and lower alpha power at occipital and temporal locations, whereas dopamine agonists were associated with higher delta activity at occipital locations and increased frontal gamma power. Serotonin antagonists showed associations with elevated slow-wave and alpha power, while serotonin agonists were linked to increased frontal alpha, decreased occipital alpha, and enhanced temporal gamma power. Both norepinephrine antagonists and agonists showed positive relationships with delta power, with a broader topographical pattern for antagonists. Theta power was positively associated with norepinephrine antagonists and negatively associated with norepinephrine agonists. Norepinephrine antagonists were related to lower temporal alpha and higher frontal and parietal gamma power. Histamine antagonists and mixed histaminergic agents were associated with lower delta, theta, and alpha power. Acetylcholine antagonists were linked to higher delta, theta, and alpha power across electrode locations.

**Discussion:**

Modeling psychotropic medication effects on EEG at the neurotransmitter receptor level offers a biologically grounded and clinically relevant improvement over traditional drug class-based approaches. This neurotransmitter-centric framework enhances mechanistic interpretability and may support the development of EEG biomarkers for personalized, mechanism-based psychiatric care.

## 1 Introduction

Understanding the modulation of brain activity by psychotropic medications remains a central challenge in psychiatric neuroscience, with direct implications for both clinical practice and biomarker development. Electroencephalography (EEG) is widely recognized as a sensitive, non-invasive tool for capturing neural oscillatory dynamics and tracking the effects of pharmacological interventions in real time, offering high temporal resolution and the ability to detect subtle neurophysiological changes across diverse clinical populations (1, 2). EEG has been instrumental in identifying characteristic alterations in brain rhythms associated with various neuropsychiatric conditions and pharmacotherapies, such as increased beta activity following antipsychotic administration or reduced alpha power with certain antidepressants (1, 3).

Despite decades of pharmaco-EEG research, the majority of studies have centered on broad therapeutic drug classes—e.g. antipsychotics, antidepressants, mood stabilizers or individual drugs—rather than the underlying neurochemical mechanisms that drive these EEG changes. This therapeutic class or single drug based focus usually overlooks the pharmacodynamic complexity and heterogeneity inherent in modern psychopharmacological regimens, where most agents act on multiple neurotransmitter systems simultaneously and patients frequently receive polypharmacy (4). For example, most antipsychotics do not only block the dopamine D2 receptor, but typically also affect receptors of other neurotransmitter systems such as the serotonin, acetylcholine, norepinephrine or histamine system (5). Similar pleiotropic effects can be seen for many antidepressant drugs (6).

As different neurotransmitters may differentially shape neural oscillations across frequency bands and brain regions (7), frequency band-specific EEG signatures associated with the manipulation of specific neurotransmitter pathways may be obscured when analyses are restricted to therapeutic drug classes or individual drug comparisons.

This lack of biological specificity has limited the clinical translation of EEG-based biomarkers, particularly their potential to inform treatment selection or monitor pharmacological response. A potential solution may be the assessment of EEG-recordings for drugs with more selective behavior such as selective serotonin reuptake inhibitors (SSRIs). For instance, SSRIs such as escitalopram have been associated with early alterations in alpha and theta activity, linked to mood regulation and cognitive processing (1, 6) while dopaminergic agents including L-DOPA, apomorphin, and SKF-38393 (agonists) as well as haloperidol and raclopride (antagonists) influence beta and gamma oscillations implicated in attention and executive function (7). Traditional therapeutic class-based analyses risk conflating these distinct neurochemical actions, impeding the identification of mechanistically meaningful EEG signatures and limiting the development of personalized biomarkers.

The present study aims at disentangling these distinct neurochemical effects and moving toward a more mechanistically informed, individualized understanding of medication-induced brain changes. By systematically coding medications according to their primary pharmacodynamic actions at specific neurotransmitter receptors, it becomes possible to map the differential influence of serotonergic, dopaminergic, noradrenergic, histaminergic, and cholinergic systems on EEG spectral power patterns, even within complex polypharmacy contexts and clinical heterogeneity. This neurotransmitter-centered classification approach follows the principles of the Neuroscience-based Nomenclature NbN (8), which organizes psychotropic drugs according to their primary neurotransmitter receptor targets rather than clinical indications. By applying this framework, our study aims to identify medication-specific neural signatures that may guide individualized treatment selection through matching patients’ EEG response profiles with drugs sharing similar neurotransmitter mechanisms.

The present study addresses this gap by integrating detailed, session-level medication profiles—systematically coded according to primary neurotransmitter-receptor actions—with large-scale EEG data from the Temple University Hospital Corpus, which contains over 26,846 anonymized EEG data files.

Whereas typical pharmaco-EEG studies rely on small samples and acute drug challenges, our analysis captures chronic medication exposure and pools recordings across multiple psychotropic agents, thereby markedly enlarging the effective sample size for each neurotransmitter system. We test whether modeling spectral-power changes as a function of neurotransmitter profiles provides a more accurate, biologically grounded account of psychotropic drug effects than traditional therapeutic-class comparisons (2).

The primary objective of this research is to characterize the frequency- and region-specific EEG signatures associated with aggregated serotonergic, dopaminergic, noradrenergic, histaminergic, and cholinergic modulation in a real-world clinical context. We hypothesize that a neurotransmitter-centric analytic framework will reveal distinct, mechanistically plausible patterns of EEG modulation that are not captured by conventional drug class labels, and that these patterns will demonstrate translational potential for the development of more precise biomarkers and personalized treatment strategies in psychiatry. Accordingly, the present work is designed as a large-scale, exploratory mapping study rather than a confirmatory or predictive modeling effort.

## 2 Methods

### 2.1 Data source and study population

The present study utilized data from the Temple University Hospital EEG Corpus (TUH-EEG), one of the largest publicly available clinical EEG datasets worldwide (2). The TUH-EEG Corpus comprises over 26,846 EEG recording sessions, encompassing a wide age range from infants to elderly adults and a diverse spectrum of neurological and psychiatric conditions. This heterogeneity reflects real-world clinical variability, including differences in recording conditions and patient demographics, thereby providing a robust foundation for computational modeling and pharmaco-EEG analyses.

EEG data within the corpus are organized hierarchically by patient and session, with each session containing one or more EEG files in EDF format alongside de-identified physician reports. The dataset includes recordings with varying channel counts (typically 31 channels) and sampling rates (predominantly 250 Hz), as well as annotations for signal events and artifacts.

Inclusion criteria for this study encompassed all EEG sessions with available corresponding medication data and sufficient signal quality for spectral analysis. Sessions were excluded if critical metadata were missing or if the EEG recordings were deemed unusable due to excessive artifacts. Patients with multiple drug exposures were not excluded, reflecting the clinical reality of polypharmacy in psychiatric populations.

Ethical considerations were addressed by utilizing the fully de-identified TUH-EEG dataset, which complies with HIPAA standards for patient privacy and confidentiality. The use of this publicly available dataset for secondary analysis was approved by the institutional review board of Temple University Hospital, ensuring adherence to ethical research practices.

### 2.2 Psychopharmacological data extraction and coding

By integrating detailed, session-level psychopharmacological profiles with EEG data, this study accounts for both single-drug and multi-drug effects.

Medication data were systematically extracted from the de-identified physician reports accompanying each EEG session in the TUH-EEG Corpus. For every patient and session, all prescribed psychotropic medications were identified and cataloged, with particular attention to antipsychotics, antidepressants, and other central nervous system (CNS)-active agents. This comprehensive approach reflects the clinical reality of both monotherapy and polypharmacy in psychiatric populations (Obeid and Picone, 2016).

Drug receptor properties were obtained from the Neuroscience-based Nomenclature (NbN) search engine https://nbn2r.com/search (8) and double-checked for accuracy by a human expert. Each medication was coded according to its primary pharmacodynamic actions on the different receptors, transporters or enzymes related to the serotonin, dopamine, norepinephrine, histamine, and acetylcholine systems. For each postsynaptic receptor, the effect was coded as neutral, agonistic, antagonistic or mixed (for partial agonists). Inhibitors of presynaptic transporters (e.g. SERT), presynaptic autoreceptors (e.g., alpha2, 5HT1) or degrading enzymes (e.g. acetylcholinesterase) were coded as agonistic due to their positive effect on postsynaptic neurotransmitter signaling. Accordingly, agonists of presynaptic autoreceptors were coded as antagonistic. For muscarinic receptors (M1–M4), all drugs were coded as either neutral or antagonists; although antagonism at presynaptic M2 and M4 autoreceptors increases acetylcholine release, the net effect was classified as antagonistic for consistency (9). Mirtazapine’s overall serotonergic effect was coded as mixed, reflecting its dual action as an alpha2 heteroreceptor antagonist (leading to increased serotonin release), and 5-HT2C antagonist (10) (see **Supplementary Tables 1**-**S5**). Finally, based on the codings for each receptor, transporter or enzyme, a total categorical score was assigned to each neurotransmitter system for each drug. If all receptor codings for a given neurotransmitter were either agonistic or antagonistic, the total effect for that neurotransmitter system in a session was coded accordingly as agonistic or antagonistic, respectively. If at least one receptor showed a mixed effect, or if both agonistic and antagonistic effects were present among the receptors, the total effect was coded as a mixed effect. This receptor-level approach provides a biologically meaningful and nuanced representation of each session’s overall pharmacological profile, in line with contemporary methodological recommendations (11, 12), which emphasize pharmacodynamic modeling rather than categorical drug classifications to capture the underlying mechanisms of action more accurately.

### 2.3 Computational pipeline workflow

**Figure 1:**
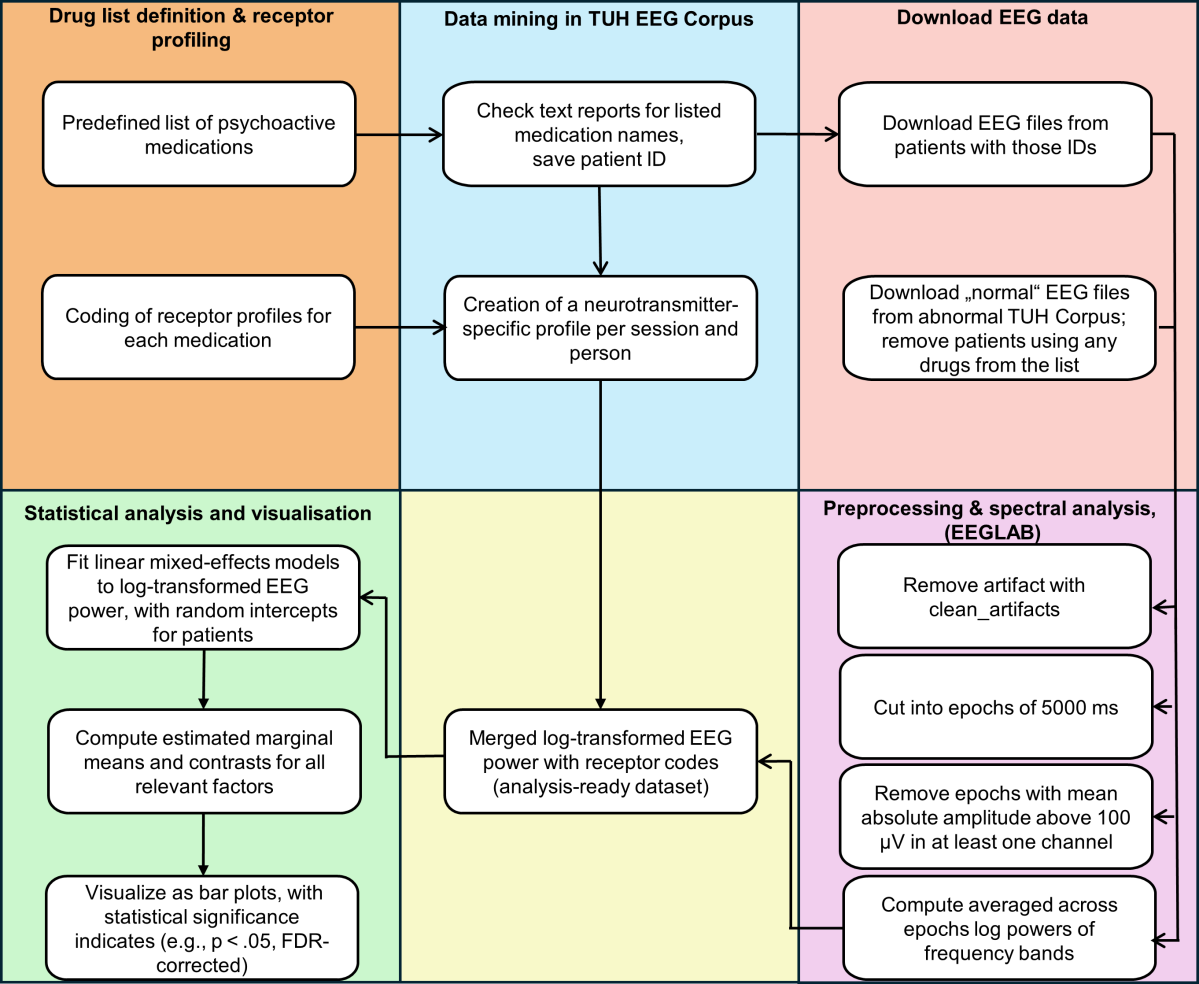
Computational pipeline for the analysis of psychotropic medication effects on EEG spectral power. The workflow illustrates the stepwise integration of medication data extraction, receptor profile coding, EEG preprocessing, spectral analysis, and statistical modeling. Medication profiles were systematically coded at the neurotransmitter receptor level for each session, and EEG data were processed to extract log-transformed power values across canonical frequency bands. Linear mixed-effects models were fitted to assess the influence of neurotransmitter modulation on EEG activity, accounting for electrode location and patient-level variability. Adapted from “Spectral changes in electroencephalography linked to neuroactive medications: A computational pipeline for data mining and analysis” by Maxion et al., Licensed under CC BY-NC-ND 4.0, 10.1016/j.cmpb.2024.108319 (4).

The computational pipeline (see **Figure 1**), adapted from Maxion et al. (4), was designed to provide a reproducible, and empirically rigorous framework for analyzing the effects of psychotropic medications on EEG spectral power.

In the first step, drug receptor properties were obtained and coded as described above (see section 2.2). EEG recordings were selected from the Temple University Hospital EEG Corpus (TUH-EEG). All sessions in which patients were administered psychotropic medications from a predefined list were included. In addition, EEG recordings labeled as “normal” and free from any psychotropic medication exposure were extracted to serve as unmedicated reference data (use of non-CNS-active drugs such as aspirin was permitted).

EEG unmedicated recordings labeled as “normal” were annotated based on routine clinical EEG assessment to exclude overt pathological patterns. While the unmedicated recordings do not represent healthy controls and may introduce systematic bias, comparisons within a shared clinical population may partially control for disease-related EEG alterations that are present in both medicated and unmedicated patients, thereby highlighting relative medication-associated effects rather than absolute deviations from health (13, 14).

EEG preprocessing and spectral analysis were performed in MATLAB using the EEGLAB toolbox. Artifacts were removed from the continuous EEG using the *clean_artifacts* function, and the cleaned data were segmented into consecutive, non-overlapping 5-s epochs. Epochs exceeding a mean absolute amplitude of 100 µV in any channel were excluded to minimize residual noise. For each retained epoch and electrode, power spectral density was computed across canonical frequency bands—delta (1–4 Hz), theta (4–8 Hz), alpha (8–12 Hz), beta (13–30 Hz), and gamma (30–100 Hz). Band-specific power values were log-transformed to approximate normality and then averaged across valid epochs, resulting in robust spectral power estimates per electrode and session.

The resulting band power values were log-transformed to normalize their distribution and then averaged across all valid epochs, yielding robust, session-level spectral features for subsequent statistical modeling.

The log-transformed EEG power values were subsequently merged with the pharmacological receptor-coding information. This merging step produced a comprehensive, analysis-ready dataset containing both medicated and unmedicated (“normal”) recordings, with all receptor codes set to 0 for the latter. This dataset contained 4128 recordings from 2083 patients.

For each frequency band, a linear mixed-effects model was fitted with the log-transformed EEG power as the dependent variable using R lme4 package (15). Fixed effects included electrode location (frontal, central, temporal, parietal, occipital) and the total neurotransmitter scores for dopamine, serotonin, norepinephrine, histamine, and acetylcholine, as well as their interactions with electrode location. Patient ID was entered as a random intercept to account for repeated measures within participants. The model specification was as follows:

log(Power) ~ ElectrodeLocation * TotalDopamine* + ElectrodeLocation * TotalSerotonin + ElectrodeLocation* TotalNorepinephrine + ElectrodeLocation* TotalHistamine + ElectrodeLocation* TotalAcetylcholine + (ElectrodeLocation | Electrode) + (1 | PatientNr).

Estimated marginal means and pairwise contrasts were computed using the R emmeans package (16), contrasts are reported throughout as z ratios based on asymptotic degrees of freedom, with Bonferroni-corrected p-values controlling for multiple comparisons. Results were visualized as 95% confidence intervals of estimated marginal means, with statistical significance indicated directly in the figures (e.g. p <.05, Bonferroni-corrected).

To evaluate the robustness of the estimated marginal means contrasts, a nonparametric bootstrap procedure was applied. For each bootstrap sample, the linear mixed-effects model was re-estimated, and the corresponding estimated marginal means contrasts were recomputed. For each contrast, bootstrap-derived estimates were used to compute empirical 95% confidence intervals based on the percentile method.

To assess robustness, we evaluated (i) the directional consistency between bootstrap-derived and original model estimates, and (ii) whether contrasts that were statistically significant in the original model exhibited bootstrap confidence intervals excluding zero (see **Supplementary Tables 11**-**S15**).

As a complementary robustness analysis, a simplified mixed-effects model was fitted for each frequency band using the same fixed-effects structure as the primary model but a reduced random-effects specification including a random intercept on the patient level only. This approach was used to mitigate potential overparameterization and singular fit issues associated with the more complex random-effects structure, and to evaluate the stability of the observed effects under a more parsimonious specification. The simplified models were estimated on the same dataset as the primary analyses.

In addition, the beta frequency band (13–30 Hz) was further subdivided into two sub-bands (13–19 Hz and 19–30 Hz) to explore potential frequency-specific effects within the beta range. These sub-band analyses were conducted using the simplified model only due to singular fit issues in the more complex model.

All results from the simplified models, including the beta sub-band analyses, are reported in the **Supplementary Materials** (see **Supplementary Results** and **Supplementary Tables 16**-**S21**).

### 2.4 Data availability

All code used for data analysis can be retrieved from https://github.com/rwth-imi/Drug_induced_spectral_changes_TUH.

## 3 Results

### 3.1 Descriptive statistics

A total of 4128 EEG recordings from 2083 patients were included in the final analysis (see **Table 1**). Across all recordings, dopaminergic modulators were observed in 2276 recordings, serotonergic in 3568, noradrenergic in 2698, histaminergic in 1502, and cholinergic in 592 patients.

**Table 1:** Drug groups with corresponding drugs and their trade names, number of files downloaded and cleaned, as well as number of patients before and after outlier removal.

Drug	Trade names	Down-loaded files	Cleaned files	patients	Patients after outlier removal
Normal					
		1521	1521 w/o meds: 881	840	632
Group AP					
Aripiprazole	Abilify, Abilify Maintena, Aristada, Aristada Initio	196	189	84	63
Clozapine	Clozaril, Clopine, FazaClo, Clozapine Synthon	64	62	22	18
Haloperidol	Haldol, Haldol Decanoate, haloperidol lactate	622	613	325	313
Olanzapine	Zyprexa, Zyprexa Zydis, Zyprexa Relprevv, Zyprexa Intramuscular	218	174	83	82
Quetiapine	Seroquel, Seroquel XR	788	777	315	267
Risperidone	Risperdal, Risperdal Consta, Perseris, Risperdal M-Tab	946	392	84	62
Ziprasidone	Geodon, Zeldox	53	50	32	19
Group AD					
Bupropion	Wellbutrin, Wellbutrin XL, Wellbutrin SR, Zyban	120	119	59	37
Escitalopram	Lexapro	295	284	111	72
Fluoxetine	Prozac, Prozac Weekly, Sarafem, Rapiflux	568	377	152	146
Mirtazapine	Remeron, Remeron SolTab	192	188	104	88
Paroxetine	Paxil, Paxil CR, Brisdelle, Pexeva	220	217	111	110
Sertraline	Zoloft, sertraline hydrochloride	638	614	265	263
Trazodone	Desyrel, Oleptro, Desyrel Dividose	482	473	202	152
Venlafaxine	Effexor XR, Effexor, venlafaxine hydrochloride	178	176	90	63

AP, antipsychotics; AD, antidepressants.

A detailed breakdown of neurotransmitter-specific modulators (antagonistic, neutral, agonistic, and mixed) across all recordings and patients is provided in **Table 2**.

**Table 2:** Overview of neurotransmitter modulation by psychotropic drugs across recordings.

Effect	Dopamine	Serotonin	Norepinephrine	Histamine	Acetylcholine
Antagonist	1422/761	335/194	773/450	865/439	592/281
Neutral	1852/923	560/412	1430/792	2626/1405	3536/1821
Agonist	566/310	1534/713	440/207	0/0	0/0
Mixed	288/150	1699/837	1485/710	637/291	0/0

Values represent the number of cleaned files followed by the number of patients after outlier removal. Note that the sum of patients for each neurotransmitter may exceed the total number of patients as medication may change between recording sessions.

### 3.2 Frequency band-specific effects

#### 3.2.1 Delta frequency band (1-4 Hz)

**Figure 2:**
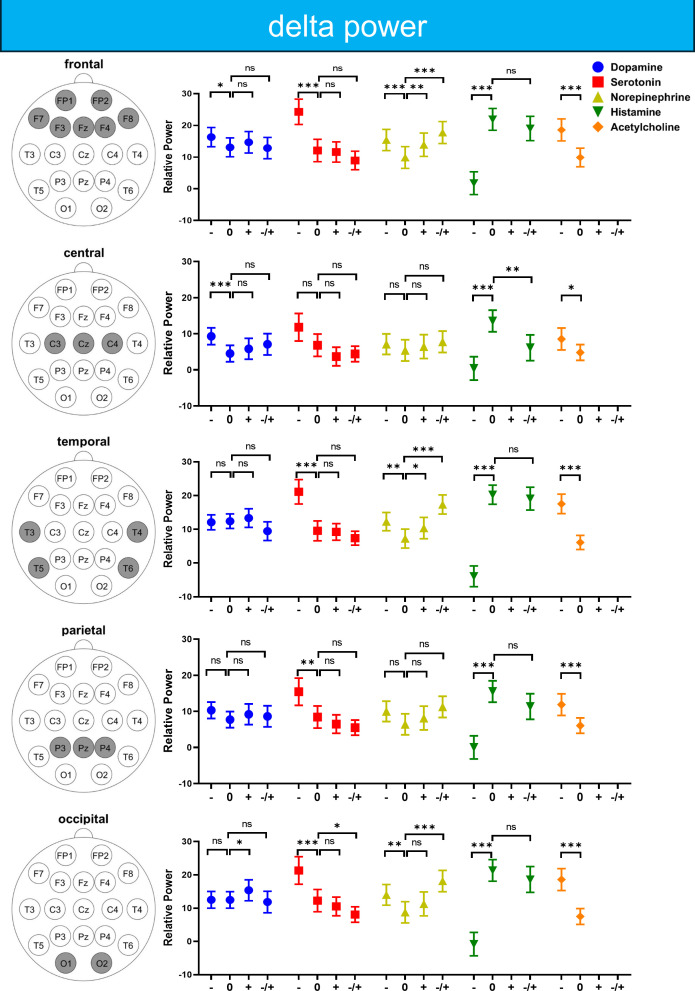
Neurotransmitter effects on delta power. Estimated marginal means of delta-band (1–4 Hz) power ± 95% CI across electrode locations (frontal, central, temporal, parietal, occipital) for neurotransmitter effect categories: antagonistic (–), neutral (0), agonistic (+), mixed (–/+) according to the linear mixed-effects model (N = 2083 patients). All p-values are Bonferroni-corrected: *p <.05, **p <.01, ***p <.001; ns, not significant.

In the delta frequency band (see **Figure 2**; **Supplementary Table 6**), the linear mixed-effects model revealed a significant main effect of electrode location, *F*(4, 32) = 15.34, *p* <.001. Significant main effects were also observed for dopamine, *F*(3, 12,771) = 3.01, *p* = .029; serotonin, *F*(3, 7,580) = 18.89, *p* <.001; norepinephrine, *F*(3, 8,518) = 5.38, *p* = .001; histamine, *F*(2, 10,843) = 54.88, *p* <.001; and acetylcholine, *F*(1, 11,119) = 32.93, *p* <.001.

All neurotransmitter systems further showed significant interactions with electrode location, including dopamine × electrode location, *F*(12, 72,727) = 3.91, *p* <.001; serotonin × electrode location, *F*(12, 72,728) = 4.75, *p* <.001; norepinephrine × electrode location, *F*(12, 72,727) = 3.16, *p* <.001; histamine × electrode location, *F*(8, 72,727) = 19.86, *p* <.001; and acetylcholine × electrode location, *F*(4, 72,730) = 9.75, *p* <.001, indicating region-specific modulation of delta power.

##### 3.2.1.1 Region-specific *post hoc* patterns

Dopamine antagonists were associated with increases in delta power at frontal (*z* = 2.86, *p* = .013) and central sites (*z* = 3.71, *p* <.001), with no significant effects at temporal, parietal, or occipital locations. Dopamine agonists showed an increase at occipital sites (*z* = 2.47, *p* = .040), but not at frontal, central, temporal, or parietal regions. Mixed dopaminergic agents did not show significant effects.

Serotonin antagonists were associated with increased delta power at frontal (*z* = 6.39, *p* <.001), temporal (*z* = 5.64, *p* <.001), parietal (*z* = 3.26, *p* = .003), and occipital sites (*z* = 3.87, *p* <.001), whereas the central effect did not remain significant after correction for multiple comparisons (*p* = .063). Serotonin agonists showed no significant effects, and mixed serotonergic agents showed a decrease at occipital sites (*z* = -2.56, *p* = .031).

Norepinephrine-related effects showed region-specific increases in delta power. Antagonists were associated with increases at frontal (*z* = 3.99, *p* <.001), temporal (*z* = 3.41, *p* = .002), and occipital (*z* = 3.16, *p* = .005) sites. Agonists showed increases at frontal (*z* = 3.56, *p* = .001) and temporal (*z* = 2.58, *p* = .029) locations. Mixed effects were observed at frontal (*z* = 3.86, *p* <.001), temporal (*z* = 4.63, *p* <.001), and occipital (*z* = 3.80, *p* <.001) sites.

Histamine antagonists were associated with reductions in delta power across all regions, including frontal (*z* = -8.51, *p* <.001), central (*z* = -4.85, *p* <.001), temporal (*z* = -9.42, *p* <.001), parietal (*z* = -5.74, *p* <.001), and occipital (*z* = -7.51, *p*<.001). Mixed histaminergic effects were observed at central sites only (*z* = -3.43, *p* = .001).

Acetylcholine antagonists were associated with increases in delta power across all regions, including frontal (*z* = 5.74, *p* <.001), central (*z* = 2.15, *p* = .031), temporal (*z* = 6.97, *p* <.001), parietal (*z* = 3.37, *p* <.001), and occipital (*z* = 5.92, *p* <.001).

The simplified model confirms the robustness of the primary findings, reproducing the core topographical and neurotransmitter-specific patterns, with differences primarily reflecting reduced model complexity rather than qualitative changes in effects. For detailed results from the simplified model, see **Supplementary Results** and **Supplementary Table 16**.

#### 3.2.2 Theta frequency band analysis (4-8 Hz)

**Figure 3:**
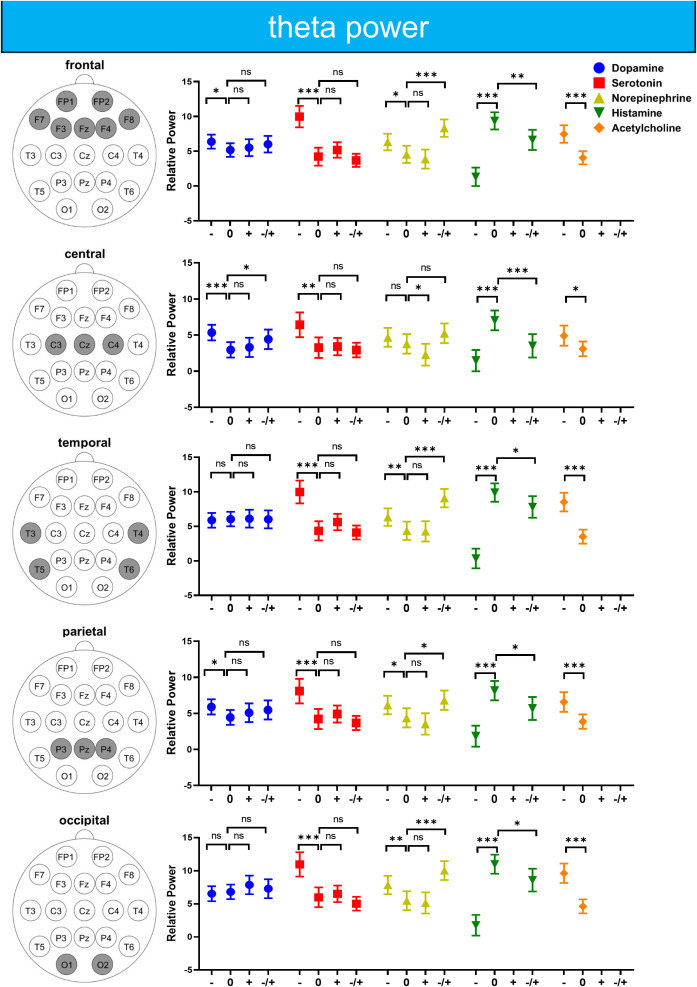
Neurotransmitter effects on theta power. Estimated marginal means of theta-band (4-8 Hz) power ± 95% CI across electrode locations (frontal, central, temporal, parietal, occipital) for neurotransmitter effect categories: antagonistic (–), neutral (0), agonistic (+), mixed (–/+).according to the linear mixed-effects model (N = 2083 patients). All p-values are Bonferroni-corrected: *p <.05, **p <.01, ***p <.001; ns, not significant.

Within the theta frequency range (see **Figure 3**; **Supplementary Table 7**), the mixed-effects model revealed a significant main effect of electrode location, *F*(4, 20) = 10.78, *p* <.001. Dopamine did not show a significant main effect, *F*(3, 14,115) = 1.79, *p*= .147, whereas significant main effects were observed for serotonin, *F*(3, 8,181) = 19.88, *p* <.001; norepinephrine, *F*(3, 9,327) = 7.97, *p* <.001; histamine, *F*(2, 12,001) = 36.48, *p* <.001; and acetylcholine, *F*(1, 12,272) = 32.04, *p* <.001.

All neurotransmitter systems further showed significant interactions with electrode location, including dopamine × electrode location, *F*(12, 72,719) = 3.97, *p* <.001; serotonin × electrode location, *F*(12, 72,720) = 3.04, *p* <.001; norepinephrine × electrode location, *F*(12, 72,720) = 2.75, *p* <.001; histamine × electrode location, *F*(8, 72,719) = 10.12, *p* <.001; and acetylcholine × electrode location, *F*(4, 72,722) = 8.84, *p* <.001.

##### 3.2.2.1 Region-specific *post hoc* patterns

Dopaminergic agents demonstrated region-specific effects. Dopamine antagonists were associated with increased theta power at frontal (*z* = 2.41, *p* = .048), central (*z* = 4.19, *p* <.001), and parietal (*z* = 2.58, *p* = .030) sites, but not at temporal (*z* = -0.33, *p* = 1.000) or occipital (*z* = -0.45, *p* = 1.000) sites. Mixed dopaminergic agents showed an increase at central sites only (*z* = 2.48, *p* = .040), whereas agonists showed no effects.

Serotonergic antagonists showed widespread increases in theta power including frontal (*z* = 6.75, *p* <.001), central (*z* = 3.29, *p* = .003), temporal (*z* = 6.15, *p* <.001), parietal (*z* = 4.05, *p* <.001), and occipital (*z* = 4.80, *p* <.001) sites. Agonists and mixed compounds showed no effects.

Norepinephrine-related effects showed regionally differentiated patterns. Antagonists were associated with increases at frontal (*z* = 2.86, *p* = .013), temporal (*z* = 2.99, *p* = .008), parietal (*z* = 2.60, *p* = .028), and occipital (*z* = 3.20, *p* = .004) sites, whereas agonists were associated with decreases at central sites (*z* = -2.67, *p* = .023). Mixed compounds showed increases at frontal (*z* = 4.13, *p* <.001), temporal (*z* = 4.86, *p* <.001), parietal (*z* = 2.41, *p* = .048), and occipital (*z* = 4.15, *p* <.001) sites.

Histaminergic agents showed reductions in theta power across all regions. Antagonists were associated with decreases at frontal (z = -7.64, p <.001), central (z = -4.64, p <.001), temporal (z = -8.38, p <.001), parietal (z = -5.28, p <.001), and occipital sites (z = -7.08, p <.001). Mixed compounds showed a similar pattern, with decreases observed at frontal (z = -3.24, p = .002), central (z = -3.69, p <.001), temporal (z = -2.29, p = .045), parietal (z = -2.59, p = .019), and occipital sites (z = -2.31, p = .042).

Acetylcholine antagonists were associated with increases in theta power across all regions, including frontal (*z* = 5.05, *p* <.001), central (*z* = 2.39, *p* = .017), temporal (*z* = 6.87, *p* <.001), parietal (*z* = 3.55, *p* <.001), and occipital (*z* = 6.03, *p* <.001).

The simplified model confirms the robustness of the primary theta-band findings by reproducing the core neurotransmitter-specific and topographical patterns observed in the complex model including only minor changes of single electrode sites. For detailed results from the simplified model, see **Supplementary Results** and **Supplementary Table 17**.

#### 3.2.3 Alpha frequency band analysis (8-12 Hz)

**Figure 4:**
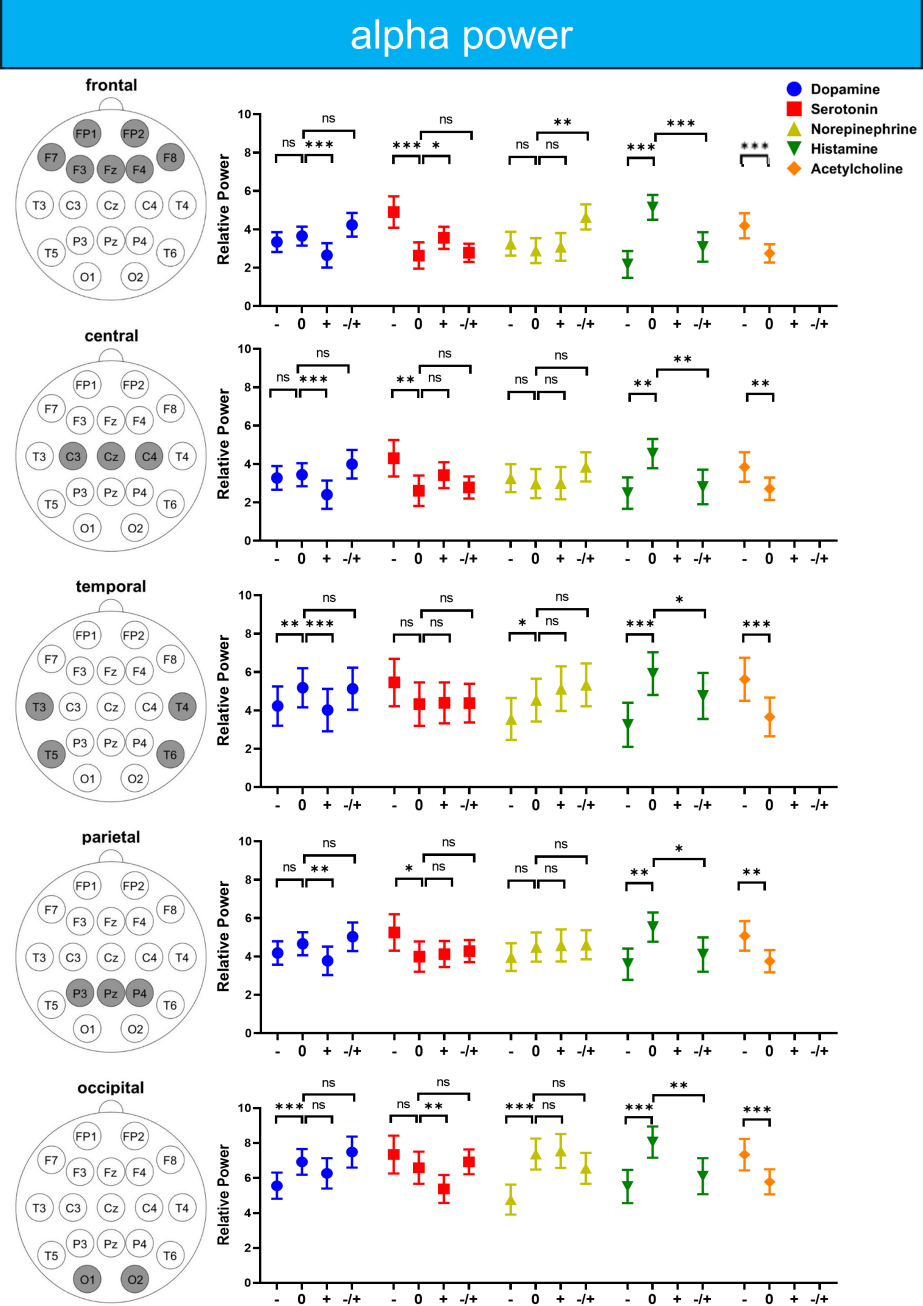
Neurotransmitter effects on alpha power. Estimated marginal means of alpha-band (8-12 Hz) power ± 95% CI across electrode locations (frontal, central, temporal, parietal, occipital) for neurotransmitter effect categories: antagonistic (–), neutral (0), agonistic (+), mixed (–/+) according to the linear mixed-effects model (N = 2083 patients). All p-values are Bonferroni-corrected: *p <.05, **p <.01, ***p <.001; ns, not significant.

Within the alpha frequency range (see **Figure 4**; **Supplementary Table 8**), the mixed-effects model revealed a significant main effect of electrode location, *F*(4, 10) = 29.93, *p* <.001. Significant main effects were observed for dopamine, *F*(3, 15,926) = 14.19, *p* <.001; serotonin, *F*(3, 8,963) = 3.92, *p* = .008; norepinephrine, *F*(3, 10,414) = 4.88, *p* = .002; histamine, *F*(2, 13,573) = 10.45, *p* <.001; and acetylcholine, *F*(1, 13,822) = 18.09, *p* <.001.

Significant interactions with electrode location were observed for dopamine × electrode location, *F*(12, 72,694) = 3.15, *p*<.001; serotonin × electrode location, *F*(12, 72,695) = 12.37, *p* <.001; norepinephrine × electrode location, *F*(12, 72,695) = 13.79, *p* <.001; and histamine × electrode location, *F*(8, 72,694) = 2.64, *p* = .007. In contrast, the acetylcholine × electrode location interaction was not significant, *F*(4, 72,697) = 1.80, *p* = .126.

##### 3.2.3.1 Region-specific *post hoc* patterns

Dopaminergic agents showed region-specific reductions in alpha power. Dopamine antagonists were associated with decreases at temporal (*z* = -3.24, *p* = .004) and occipital sites (*z* = -4.03, *p* <.001), but not at frontal, central, or parietal locations. Dopamine agonists were associated with decreases at frontal (*z* = -4.15, *p* <.001), central (*z* = -3.90, *p* <.001), temporal (*z* = -4.56, *p* <.001), and parietal sites (*z* = -3.36, *p* = .002), with a trend at occipital locations (*z* = -2.32, *p* = .062). Mixed dopaminergic compounds showed no significant effects.

Serotonergic agents showed a regionally differentiated pattern. Serotonin antagonists were associated with increases at frontal (*z* = 4.83, *p* <.001), central (*z* = 3.23, *p* = .004), and parietal sites (*z* = 2.41, *p* = .049), but not at temporal or occipital locations. Serotonin agonists were associated with an increase at frontal (*z* = 2.86, *p* = .013) and a decrease at occipital sites (*z* = -3.18, *p* = .004), with no significant effects at central, temporal, or parietal locations. Mixed serotonergic compounds showed no significant effects.

Norepinephrine-related effects showed region-specific patterns. Norepinephrine antagonists were associated with decreases at temporal (*z* = -2.70, *p* = .021) and occipital sites (*z* = -6.43, *p* <.001), but not at frontal, central, or parietal locations. Agonists showed no significant effects. Mixed compounds were associated with increases at frontal sites only (*z*= 3.48, *p* = .002).

Histaminergic agents were associated with consistent reductions in alpha power across all regions. Antagonists were associated with decreases at frontal (z = -5.16, p <.001), central (z = -3.15, p = .003), temporal (z = -4.28, p <.001), parietal (z = -2.97, p = .006), and occipital sites (z = -3.58, p = .001). Mixed compounds showed a similar pattern, with decreases observed at frontal (z = -4.45, p <.001), central (z = -3.34, p = .002), temporal (z = -2.34, p = .039), parietal (z = -2.76, p = .012), and occipital sites (z = -3.44, p = .001).

Acetylcholine antagonists were associated with increases in alpha power across all regions, including frontal (*z* = 3.88, *p*<.001), central (*z* = 2.71, *p* = .007), temporal (*z* = 4.94, *p* <.001), parietal (*z* = 3.19, *p* = .001), and occipital sites (*z* = 3.45, *p* = .001). These findings showed limited convergence with bootstrap results.

The simplified model confirms the robustness of the primary findings, reproducing the core topographical and neurotransmitter-specific patterns of alpha modulation. Observed differences were primarily quantitative, reflecting reduced model complexity, while the overall structure of effects remained stable. For detailed results from the simplified model, see **Supplementary Results** and **Supplementary Table 18**.

#### 3.2.4 Beta frequency band analysis (13–30 Hz)

**Figure 5:**
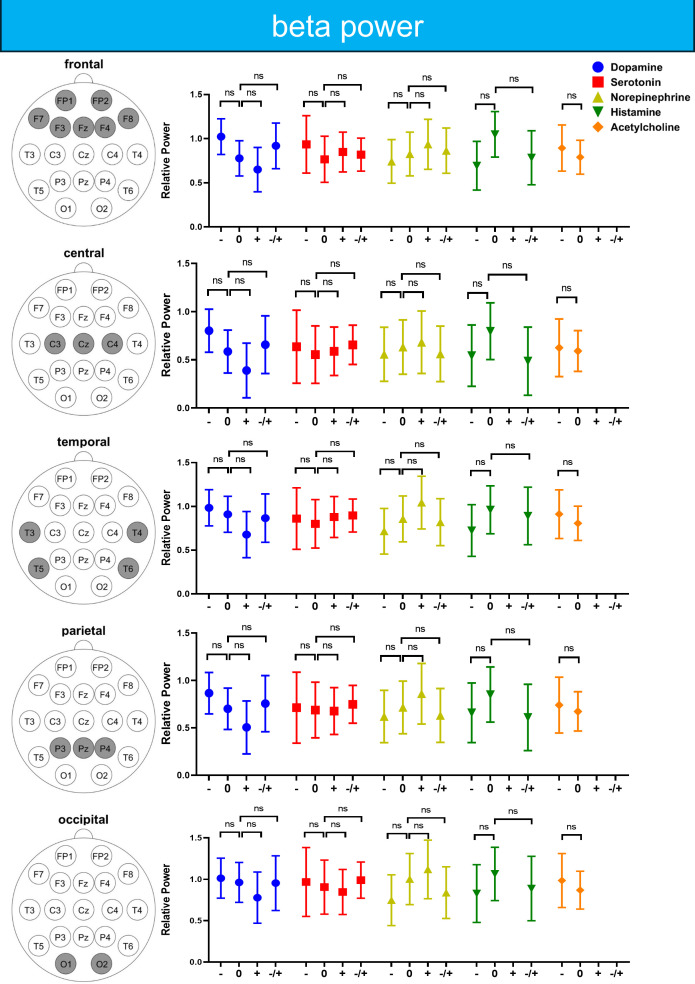
Neurotransmitter effects on beta power. Estimated marginal means of beta-band (13-30 Hz) power ± 95% CI across electrode locations (frontal, central, temporal, parietal, occipital) for neurotransmitter effect categories: antagonistic (–), neutral (0), agonistic (+), mixed (–/+)according to the linear mixed-effects model (N = 2083 patients). All p-values are Bonferroni-corrected: ns, not significant.

Within the beta frequency range (see **Figure 5**; **Supplementary Table 9**), the mixed-effects model revealed a marginal main effect of dopamine, *F*(3, 6,618) = 2.61, *p* = .050. In contrast, no significant main effects were observed for serotonin, *F*(3, 4,623) = 0.18, *p* = .911; norepinephrine, *F*(3, 4,830) = 1.27, *p* = .284; histamine, *F*(2, 5,673) = 0.95, *p* = .388; or acetylcholine, *F*(1, 5,846) = 0.42, *p* = .519.

No significant interactions between electrode location and any neurotransmitter system were observed (all *p* >.80), indicating an absence of region-specific modulation of beta power in the fitted model.

##### 3.2.4.1 Region-specific *post hoc* patterns

Dopaminergic agents showed only non-significant trends. Dopamine agonists were associated with a tendency toward reduced beta power at frontal (*z* = -1.41, *p* = .481) and central sites (*z* = -1.86, *p* = .189), whereas antagonists showed a slight tendency toward increased beta power at frontal (*z* = 2.25, *p* = .074) and central locations (*z* = 1.66, *p* = .293). Mixed dopaminergic compounds showed no significant effects across electrode locations.

Serotonergic, norepinephrine-related, histaminergic, and acetylcholine-related agents showed no significant associations with beta power across any electrode location. All pairwise contrasts remained non-significant after Bonferroni correction (all *p* >.32).

The simplified model confirms for beta-1 (13–19 Hz) and beta-2 (19–30 Hz) the overall pattern observed in the complex model: while small global dopaminergic effects can be detected, there is no evidence for significant consistent region-specific modulation. Differences between the complex and simplified models are therefore quantitative rather than qualitative, reflecting reduced model complexity rather than meaningful changes in the underlying effects. For results from the simplified model, see **Supplementary Results** and **Supplementary Tables 19**, **S20**.

#### 3.2.5 Gamma frequency band analysis (30–100 Hz)

**Figure 6:**
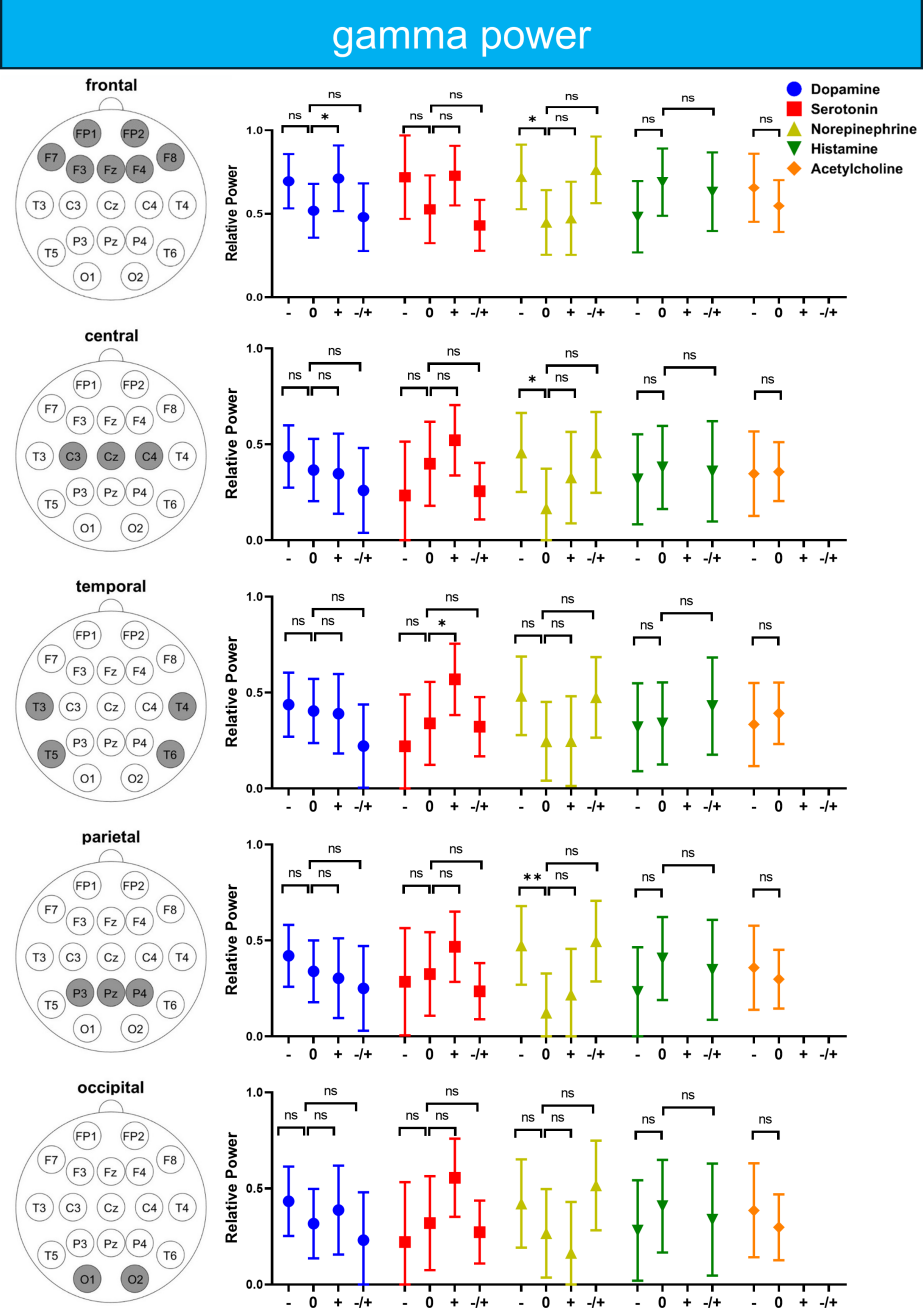
Neurotransmitter effects on gamma power. Estimated marginal means of gamma-band (30-100 Hz) power ± 95% CI across electrode locations (frontal, central, temporal, parietal, occipital) for neurotransmitter effect categories: antagonistic (–), neutral (0), agonistic (+), mixed (–/+) according to the linear mixed-effects model (N = 2083 patients). All p-values are Bonferroni-corrected: *p <.05, **p <.01, ns, not significant.

Within the gamma frequency range (see **Figure 6**; **Supplementary Table 10**), the mixed-effects model revealed a significant main effect of electrode location, *F*(4, 86) = 4.19, *p* = .004. A trend-level main effect was observed for dopamine, *F*(3, 6303) = 2.24, *p* = .081. Significant main effects were observed for serotonin, *F*(3, 4489) = 4.34, *p* = .005, and norepinephrine, *F*(3, 4661) = 3.26, *p* = .021, whereas histamine, *F*(2, 5422) = 0.44, *p* = .643, and acetylcholine, *F*(1, 5583) = 0.15, *p* = .698, showed no significant effects.

A significant interaction between electrode location and dopamine was observed, *F*(12, 72750) = 2.20, *p* = .010, whereas interactions for serotonin, norepinephrine, histamine, and acetylcholine were not significant.

##### 3.2.1 Region-specific *post hoc* patterns

Dopaminergic agents showed mixed region-specific patterns in the fitted model. Dopamine agonists were associated with increased gamma activity at frontal sites (*z* = 3.45, *p* <.001), whereas antagonists showed a non-significant increase and mixed compounds showed a non-significant trend toward reduced gamma power.

Serotonergic agents showed limited regional effects. No significant effects were observed for antagonists or mixed compounds, whereas serotonin agonists were associated with increased gamma power at frontal (*z* = 2.41, *p* = .048), and temporal sites (*z* = 3.12, *p* = .002).

Norepinephrine-related effects showed selective regional modulation in *post hoc* comparisons, despite the significant global main effect in the fitted model. Noradrenergic antagonism relative to neutral profiles was associated with increased gamma power in frontal (z = 2.88, p = .012), central (z = 2.62, p = .026), and parietal regions (z = 3.18, p = .005).

Histaminergic effects showed no significant regional modulation in *post hoc* comparisons in the gamma band. None of the original model contrasts reached statistical significance (all ps ≥.432).

Cholinergic effects showed no significant regional modulation in *post hoc* comparisons in the gamma band. None of the original model contrasts reached statistical significance (all ps ≥.313).

The simplified model partially reproduces the pattern observed in the complex model, confirming global effects for serotonin and norepinephrine and a weaker dopaminergic contribution. However, while the simplified model suggests additional interaction effects for dopamine and serotonin, these were not consistently supported across regions and contrast levels. For detailed results from the simplified model, see **Supplementary Results** and **Supplementary Table 21**.

#### 3.2.6 Bootstrap robustness analyses

The bootstrap analyses provided an additional robustness check by repeatedly refitting the full mixed-effects model on resampled data. Across all frequency bands and contrasts, 100% of bootstrap-derived contrast estimates showed the same direction as the corresponding original estimated marginal means contrasts.

In addition, 100% of the contrasts that reached statistical significance in the original model were associated with bootstrap-derived 95% confidence intervals excluding zero, whereas effects that were not statistically supported in the primary model were not corroborated by the bootstrap analyses, with confidence intervals consistently including zero.

This pattern indicates that the bootstrap results did not simply reproduce significance patterns, but systematically differentiated between stable and non-stable effects, thereby suggesting the robustness of the estimated contrasts under bootstrap resampling. Detailed results are provided in **Supplementary Tables 11**-**S15**.

## 4 Discussion

This neurotransmitter-centric pharmaco-EEG analysis demonstrated frequency- and region-specific modulation of EEG spectral power by psychotropic agents, providing mechanistic insights and identifying candidate biomarkers for personalized psychiatry. Consistent with prior literature, each neurotransmitter system exhibited distinct spectral signatures that may inform both pathophysiology and treatment monitoring (17).

### 4.1 Dopaminergic effects on neural oscillations across frequency bands

#### 4.1.1 Delta and theta band

Administration of dopamine antagonists in our antipsychotic-treated cohort, was associated with prominently increased slow-wave activity at central electrode sites with additional involvement of frontal electrodes for the delta band and parietal electrodes for the theta band. In contrast, dopamine agonists were associated with higher delta power at occipital electrodes only.

Increase of theta band activity is one of the most consistent findings for antipsychotics with high dopamine D2 receptor affinity such as haloperidol (18–22) or amisulpride (23) and has been linked to some extent to treatment response in patients with schizophrenia (24).

Similarly, increases in slow-wave activity were reported for healthy subjects after intake of chlorpromazine, haloperidol, or sulpiride (22). Concerning the delta frequency band, the literature is less consistent with some studies reporting increased (19, 25, 26) and others reporting decreased delta activity after treatment with D2-/D3 antagonists (18, 27). Such divergent findings may be related to differences in patient population characteristics, treatment duration, and EEG recording parameters as well as the considerably lower sample sizes as compared to our dataset.

#### 4.1.2 Alpha-band

For dopamine antagonists, significantly lower alpha-band power emerged at occipital and temporal electrodes. Previous studies on dopamine antagonists have demonstrated heterogeneous alpha-band effects. For haloperidol, both increases (28) and decreases have been reported (19). Similarly, while risperidone has been associated with increases in centro-parietal alpha rhythms (29), our previous study identified lower alpha power across multiple electrode positions in patients treated with risperidone. However, it should be noted that these findings are based on the same dataset presented here.

Again, methodological variations including EEG montage configurations, dosing protocols, acute versus chronic administration paradigms, regional specificity of dopaminergic modulation, distinct off-target receptor binding profiles among individual antagonists as well as the much higher sample size in our study have to be taken into account.

Additionally, we observed widespread decreases in alpha power for dopamine agonists. However, our dataset did not include any pure dopamine agonists (e.g., L-Dopa). Instead, the observed effects primarily stem from antidepressants with a dopaminergic reuptake inhibition component, such as bupropion. Notably, bupropion has been shown to significantly reduce absolute alpha-2 power in a previous study (30).

#### 4.1.3 Beta and gamma band

For dopamine antagonists, a trend towards higher beta and gamma power was observed which would match relative beta power increases documented for amisulpride (6) as well as beta 1 and gamma power increases documented for haloperidol (31) (32). Dopamine agonists were associated with significantly higher frontal gamma power, as well as a trend towards lower beta power which is in line with findings of increased gamma and decreased beta power induced by treatment with L-Dopa in rodent parkinson disease models (33).

### 4.2 Serotonergic effects on neural oscillations across frequency bands

#### 4.2.1 Delta-and theta band

Serotonin antagonists were associated with significantly higher slow wave activity across all electrode sites, closely resembling the patterns observed in patients treated with olanzapine or clozapine (34).

Serotonergic agonists did not show significant delta or theta activity changes in our study, consistent with previous reports of only marginal NREM-EEG alterations (35, 36) and mixed findings in wake EEG studies (37).

#### 4.2.2 Alpha-band

In our cohort, serotonin antagonists, showed robust and statistically significant alpha power increases at frontal, central, and parietal electrode sites. This widespread alpha enhancement pattern aligns with established pharmaco-EEG research demonstrating significantly higher alpha power in patient group treated with the antipsychotics risperidone, quetiapine, olanzapine, aripiprazole, amisulpride, paliperidone, ziprasidone and haloperidol most of which are serotonin antagonists (34, 38).

Serotonin agonists exhibited a novel regional dissociation pattern, with significant frontal alpha increases coupled with significant occipital alpha decreases. This contrasts with previous studies showing diffuse alpha power reductions following chronic SSRI treatment, including paroxetine-induced widespread alpha decreases (39) and citalopram-associated reductions in fronto-parietal EEG power density across the 5-20 Hz range (40).

#### 4.2.3 Beta and gamma-band

Beta oscillations remained largely unaffected by serotonergic modulation, with no statistically significant changes observed across agonist, antagonist, or mixed compound categories, suggesting potentially distinct neural circuit mechanisms compared to dopaminergic systems (41). Serotonergic agonists demonstrated significant gamma power increases, particularly at temporal electrode sites, consistent with established effects of escitalopram and vortioxetine on high-frequency oscillations (42).

### 4.3 Noradrenergic effects on neural oscillations across frequency bands

#### 4.3.1 Delta-band

The significant enhancement of delta power by norepinephrine agonists at frontal and temporal electrode sites represents a novel finding in our study which may appear counterintuitive given norepinephrine’s stimulating effects on arousal. Importantly, our study investigates the effects of chronic treatment, which may yield opposing results compared to those observed following acute administration.

The clinical literature provides partial support, as atomoxetine—a selective norepinephrine reuptake inhibitor—has been shown to increase delta activity in children with ADHD (43). Similarly, clomipramine—a noradrenergic/serotonergic reuptake inhibitor—has been reported to produce an increase in power in the delta frequency range that was correlated with clinical responsiveness (44).

Notably, both norepinephrine agonists and antagonists showed significant delta power enhancement—agonists primarily at frontal and temporal sites, and antagonists exhibiting broader effects that extended to occipital regions—contradicting simple agonist–antagonist models and implying complex receptor-mediated mechanisms. The observed regional specificity, with frontal and temporal predominance, aligns with anatomical studies demonstrating that locus coeruleus (LC) projections to the cortex are nonuniform. Early retrograde tracing in rodents revealed denser noradrenergic innervation in prefrontal cortices compared to motor and somatosensory regions (45). More recent viral-genetic tracing confirmed that distinct LC neuronal subpopulations preferentially target prefrontal versus motor areas, enabling independent modulation of executive and motor circuits (46). Finally Chandler (47) reviewed evidence showing that, despite widespread LC reach, axonal density and receptor subtype expression vary regionally, supporting the notion that noradrenergic modulation of slow-wave oscillations differs across cortical territories.

#### 4.3.2 Theta-band

Noradrenergic agonists were associated with reduced theta power in our cohort, in line with preclinical findings demonstrating that increased noradrenergic transmission can suppress hippocampal formation (HPC) type 2 theta rhythms (48). Comparable effects on theta power have also been reported for bupropion, further supporting the relevance of catecholaminergic signaling in modulating cortical oscillatory dynamics (30).

#### 4.3.3 Alpha-band

Noradrenergic mixed modulators were associated with significant increases in frontal alpha activity. The frontal predominance of noradrenergic alpha changes corresponds with dense noradrenergic innervation of prefrontal cortex and its involvement in attentional processes.

#### 4.3.4 Beta and gamma-band

Although selective norepinephrine reuptake inhibitors SNRI showed no statistically significant beta‐band changes, we observed subtle directional trends towards higher beta activity for NE agonists and lower beta activity for NE antagonists that warrant consideration. Indeed, previous studies demonstrated that NE reuptake inhibitors reboxetine and atomoxetine enhanced beta oscillations (43, 49).

Noradrenergic antagonists were associated with enhanced gamma oscillations (30–80 Hz) in frontal, central, and parietal cortices. While -to the best of our knowledge – findings on the effects of NE antagonists on gamma power in humans have not been reported yet, alpha1-antagonists reversed alpha 1 agonist induced reduction of gamma frequency activity in the hippocampus *in vitro* (50).

### 4.4 Histaminergic effects on neural oscillations across frequency bands

Our pharmaco-EEG data revealed that both histamine antagonists and mixed histaminergic agents were associated with a pronounced reduction in delta power and histamine antagonists were also associated with lower alpha power. In contrast, clinical EEG studies such as Pichlmayr and Lips (51) reported that the H_1_-antagonist promethazine was associated with increased delta activity in patients. This discrepancy may be explained by the fact that many antihistaminergic drugs such as promethazine or diphenhydramine are also potent muscarinic M1 antagonists. In line with this notion, Masuoka et al. (52) demonstrated that the EEG effects of H_1_-antagonists are strongly modulated by their antimuscarinic properties: promethazine, diphenhydramine, and chlorpheniramine—all potent muscarinic antagonists—increased hippocampal theta power, whereas triprolidine, with weak muscarinic activity, decreased theta power. By modeling both histaminergic and cholinergic effects, our model may be suited to disentangle the real opposing effects of the two receptor systems.

### 4.5 Cholinergic effects on neural oscillations across frequency bands

In our cohort, acetylcholine antagonists were associated with a uniform increase in delta power across all electrode sites, consistent with human wake and sleep-EEG studies, in humans using scopolamine (53, 54), [Kikuchi et al., 1999]. Drugs with combined H_1_- and muscarinic antagonism such as promethazine have also been reported to increase delta and theta activity in clinical EEG studies (51), supporting the interpretation that its pronounced effects on slow-wave generation are largely mediated by anticholinergic mechanisms.

For the alpha band, cholinergic antagonists were associated with widespread enhancements, which is in contrast with other studies of muscarinic blockade (55)that reported alpha reduction (53, 56). Again, the discrepancy with our findings may reflect the chronic exposure in our cohort.

### 4.6 Strengths, limitations, and clinical implications

Taken together, these findings not only corroborate key patterns reported in previous pharmaco-EEG studies but also extend them by demonstrating that a neurotransmitter-centric, receptor-level approach can reveal distinct, biologically plausible EEG signatures for different pharmacological profiles. The results suggest that the action of psychotropic medications on neurotransmitter systems, rather than their broad class labels, is a more accurate determinant of EEG spectral changes. This has important implications for the development of EEG-based biomarkers and for advancing personalized medicine in psychiatry (57, 58).

Importantly, these conclusions were evaluated across multiple analytical levels, including the primary mixed-effects models, bootstrap-based robustness checks, simplified model validation, and additional beta sub-band analyses. This multi-step strategy allowed us to distinguish stable effects from findings that were sensitive to model specification.

From a diagnostic perspective, the demonstration that specific neurotransmitter systems exert distinct, frequency- and region-specific effects on EEG power provides a mechanistic basis for the development of more sensitive and specific biomarkers. Such biomarkers could ultimately contribute to a more nuanced differentiation of neuropsychiatric conditions or subtypes, based on neurochemical signatures rather than solely on symptom-based classifications. Furthermore, the ability to objectively quantify the neurophysiological impact of psychotropic medications using EEG may assist clinicians in optimizing dosing, minimizing side effects, and tailoring interventions to the neurobiological profile of each patient (59, 60).

Although primary psychiatric diagnoses were not explicitly modeled, the present large-scale analysis remains informative for several reasons. First, comparisons were performed within a shared clinical population, which may partially control for disease-related EEG alterations present in both medicated and unmedicated recordings (13, 14). Second, psychotropic medications are prescribed transdiagnostically and exert their effects at the level of neurotransmitter systems rather than diagnostic categories (61, 62). Accordingly, a neurotransmitter-centric analysis may capture biologically meaningful EEG patterns that generalize across diagnostic boundaries (63). Finally, the large and clinically heterogeneous sample—spanning a wide range of diagnoses, medication profiles, and recording conditions—introduces substantial variability that would be expected to produce diffuse or inconsistent effects if the observed associations were primarily driven by diagnosis-related confounding. In contrast, we observed structured, frequency- and region-specific patterns across multiple analytical approaches. This consistency argues against a purely confounding-driven explanation and supports the interpretation that the identified effects reflect, at least in part, systematic medication-associated modulation of EEG activity (64, 65). Together, these considerations support the interpretation of the present findings as exploratory and hypothesis-generating, providing a real-world foundation for future diagnosis-stratified and longitudinal studies.

Scientifically, the neurotransmitter-centric computational framework introduced here offers a scalable and reproducible model for integrating pharmacological, neurophysiological, and clinical data. This methodology can be extended to other datasets, populations, and neuroimaging modalities, paving the way for prospective studies that aim to validate robust, mechanistically anchored EEG biomarkers for predicting treatment response, remission, or relapse risk in psychiatric disorders. By mapping the effects of specific neurotransmitter systems on neural oscillations, future research can further elucidate the pathways linking pharmacological interventions to cognitive and affective outcomes, thereby informing both drug development and clinical trial design. The integration of this approach with genetic, imaging, or behavioral data holds promise for further enhancing the precision and predictive value of neuropsychiatric biomarkers (66, 67).

Taken together, the present findings indicate that adopting a biologically informed, receptor-level framework for analyzing psychotropic medication effects on EEG may enhance the mechanistic interpretability and clinical relevance of electrophysiological biomarkers, thereby contributing to the broader aim of advancing psychiatry toward personalized, mechanism-based care.

A major strength of this study is the use of the Temple University Hospital EEG Corpus, which represents one of the largest and most diverse clinical EEG datasets worldwide. The inclusion of over 4128 EEG sessions from more than 2083 patients ensures high statistical power and enhances the generalizability of the findings to real-world clinical populations (2, 4). Additionally, the study’s embrace of clinical heterogeneity—including polypharmacy and a wide spectrum of diagnoses—mirrors actual psychiatric practice and strengthens the ecological validity of the results.

Internal negative controls and specificity. Beyond statistically significant effects, the dataset also contained systematic null findings that can be interpreted as internal negative controls (i.e., falsification endpoints) to probe residual confounding and analytic artifacts in an observational setting (68, 69). Importantly, these null patterns were consistent across multiple analytical levels, including the complex mixed-effects models, simplified model specifications, beta sub-band decomposition, and bootstrap-based robustness analyses.

Notably, several theoretically plausible contrasts showed no measurable or no robust associations—most prominently the largely absent beta-band effects across neurotransmitter systems (dopamine, serotonin, norepinephrine, histamine, and acetylcholine). This null pattern was consistently observed in the full beta model and remained stable across beta-1 and beta-2 sub-band analyses, with only small, spatially unstructured dopaminergic effects that were not consistently supported by *post hoc* comparisons or bootstrap confidence intervals. In addition, the non-significant dopaminergic main effect in the theta band further illustrates that even biologically plausible effects do not uniformly emerge across frequency ranges.

In contrast, other frequency bands exhibited pronounced, topographically structured modulation, particularly in the delta, theta, and alpha ranges. Such structured null patterns strengthen specificity because unmeasured global factors (e.g., arousal state, sedation, or recording context) typically induce broad, state-related changes across multiple frequency components rather than selective neurotransmitter- and region-specific signatures (70, 71). In pharmaco-EEG research, treatment-related effects are known to be heterogeneous across frequency bands and studies, with beta- and gamma-band findings often less consistent than slow-wave and alpha/theta signatures, further motivating the value of within-dataset negative controls for specificity (65). Accordingly, the co-occurrence of robust band- and region-specific effects alongside systematic null findings—replicated across multiple model specifications—argues for mechanistically differentiated modulation rather than a ubiquitous, nonspecific EEG power shift.

Nevertheless, future work will explicitly quantify the stability of these mappings across diagnostic subgroups, medication combinations, and alternative modeling specifications.

Despite these strengths, several limitations must be acknowledged. The cross-sectional, observational design precludes causal inference, limiting the ability to definitively establish direct effects of specific neurotransmitter profiles on EEG outcomes. Residual confounding is possible, as unmeasured variables such as primary diagnosis, illness severity, or undocumented medication changes may influence both drug exposure and EEG patterns. Although the use of internal negative controls and structured null findings partially mitigates concerns regarding global confounding, causal interpretations remain limited.

The extraction of medication information from unstructured, free-text clinical reports introduces potential for misclassification and missing data, even with systematic coding procedures (2). Furthermore, the modeling approach aggregates effects at the neurotransmitter system level, without differentiation between individual receptor subtypes or consideration of potential interactions between neurotransmitter systems—a necessary simplification to maintain statistical power and interpretability, but one that may obscure more subtle pharmacodynamic relationships.

The study also does not incorporate medication dosage, treatment duration, or active metabolites, all of which can modulate neurophysiological effects. Moreover, while the sample is large and heterogeneous, reliance on a single clinical EEG corpus may limit the generalizability to populations or clinical contexts not represented in the dataset.

Another methodological consideration concerns the unequal representation of neurotransmitter systems in the analyzed dataset. The distribution of patients across neurotransmitter categories showed moderate imbalance, with serotonergic modulation being most prevalent (1,061 patients) and dopaminergic modulation also relatively frequent (861 patients). In contrast, noradrenergic (565), histaminergic (607), cholinergic (519), and neutral (632) groups were more evenly represented and of comparable magnitude.

Within each system, the proportion of antagonistic, neutral, agonistic, and mixed compounds varied substantially (see **Table 2**). For instance, dopaminergic modulation was primarily driven by antagonistic agents (1422 recordings), reflecting the predominance of antipsychotic medications, whereas agonistic or mixed dopaminergic compounds were less frequent.

In contrast, serotonergic modulation displayed a predominance of agonists (1534 recordings) and mixed serotonergic compounds (1699 recordings).

Systems such as noradrenergic, histaminergic, and cholinergic were characterized by a higher proportion of neutral or antagonistic profiles, while pure agonists were rare or absent.

Although such differences in sample size may influence the precision of parameter estimates for less frequent systems, mixed-effects models are generally robust to moderate imbalance, and the overall conclusions are unlikely to be driven by these imbalances (72, 73). In addition, the consistency of key findings across complex and simplified model specifications, as well as across bootstrap-based robustness analyses, suggests that the overall conclusions are unlikely to be driven by these imbalances. Nevertheless, the present analyses should be interpreted as exploratory and hypothesis-generating.

A further methodological limitation concerns the definition of frequency bands, particularly the gamma range (30–100 Hz), and, to a lesser extent, the beta range (13–30 Hz). Although the beta band was further subdivided into beta-1 (13–19 Hz) and beta-2 (19–30 Hz) in additional analyses, both sub-bands remain relatively broad and may still encompass functionally distinct oscillatory processes. Likewise, the gamma band covers a wide frequency interval within which lower and higher gamma components may reflect different neural mechanisms and pharmacological sensitivities. Aggregating effects across such broad bands may therefore mask more nuanced, frequency-specific drug effects and limit mechanistic interpretability, particularly in higher-frequency ranges where robustness was reduced (74–76).

Longitudinal studies are needed to elucidate the temporal dynamics of EEG changes in response to medication initiation, titration, and discontinuation (77). Incorporating detailed information on dosage, treatment duration, and plasma drug levels could enhance the precision of pharmacodynamic modeling (78).

Finally, an important methodological limitation relates to the exclusive focus on spectral power as the primary EEG feature. Spectral power captures only first-order amplitude information and does not directly reflect higher-order network dynamics such as phase synchronization, cross-frequency coupling, or large-scale network topology. Accordingly, the present analyses provide an indirect and partial view of neurotransmitter-specific neural dynamics, and future work will extend this framework to include connectivity-based and cross-frequency measures.

In future work, further confounding factors such as diagnosis, gender, and other demographic variables should be explicitly controlled. In addition, the present study did not include formal subsample analyses (e.g., stratification by diagnostic groups or medication classes) or cross-validation procedures to assess the stability of findings across independent data partitions. While the consistency of results across bootstrap analyses, simplified model specifications, and beta sub-band decompositions provides indirect support for robustness, future studies should explicitly incorporate subgroup analyses and cross-validation frameworks to further strengthen generalizability and assess potential heterogeneity of effects.

Integrating EEG with other modalities—such as neuroimaging, genetic, or behavioral data—could further enhance the specificity and predictive value of neuropsychiatric biomarkers (79). Additionally, future work should consider the standardization of EEG acquisition protocols and the inclusion of structured clinical and behavioral assessments to reduce variability and improve interpretability and facilitate the automated extraction of relevant metadata for integration into large-scale analyses. Open questions remain regarding the mechanisms by which specific neurotransmitter systems drive distinct oscillatory changes, as well as the influence of comorbid conditions.

## 5 Conclusion

Modeling psychotropic medication effects on EEG spectral power at the level of cumulative neurotransmitter receptor profiles offers a biologically grounded and clinically relevant advancement over traditional drug class-based approaches. By leveraging a large, heterogeneous clinical dataset and a mechanistically informed analytic strategy, we extended traditional EEG research beyond phenomenological spectral analyses toward a receptor-level framework, revealing distinct frequency- and region-specific EEG signatures for major neurotransmitter systems—including serotonin, dopamine, norepinephrine, histamine, and acetylcholine—even in the context of polypharmacy and real-world clinical variability. These findings underscore the translational potential of EEG as a biomarker platform for personalized psychiatry, supporting more precise treatment selection, monitoring, and prediction of therapeutic response through receptor-informed EEG markers that reflect the pharmacodynamic engagement of specific neurotransmitter systems.

Future work should focus on validating these results in prospective longitudinal studies that track within-subject changes over time. Such studies could integrate multimodal data by aligning EEG measures with neuroimaging-derived network activity, genetic polymorphisms, and behavioral indices for maximizing the clinical utility of EEG-based biomarkers in neuropsychiatric care.

## Data Availability

The original contributions presented in the study are included in the article/**Supplementary Material**. Further inquiries can be directed to the corresponding author.
