# Supplementary material for: A computational pipeline for a neurotransmitter-centric analysis of the effects of psychiatric medication on EEG spectral power
Source: Front Psychiatry. 2026 Jun 19;17:1737357. doi: 10.3389/fpsyt.2026.1737357 (PMC13328094; doi:10.3389/fpsyt.2026.1737357)
Supplement: Supplementary file 1 [file SupplementaryFile1.docx]

# Supplementary Materials

## Supplementary Results

## Simplified mixed-effects model

As a complementary robustness analysis to the primary mixed-effects model including electrode-level random slopes, a simplified model was fitted with an identical fixed-effects structure but a reduced random-effects specification including a random intercept for patient only. The simplified model was based on 99,832 observations from 3,026 patients.

### *Results of the simplified mixed-effects model for the delta frequency band.*

The simplified model confirmed the main findings of the primary analysis. A significant main effect of electrode location was observed, F(4, 96772) = 43.43, p < .001, indicating stable topographical differences in delta power across frontal, central, temporal, parietal, and occipital regions. Significant main effects were also present for all neurotransmitter systems, including dopamine, F(3, 37106) = 8.13, p < .001; serotonin, F(3, 29105) = 12.86, p < .001; norepinephrine, F(3, 29626) = 9.12, p < .001; histamine, F(2, 33425) = 47.69, p < .001; and acetylcholine, F(1, 30530) = 42.90, p < .001.

In line with the primary model, all neurotransmitter systems showed significant interactions with electrode location (all p < .001), supporting region-specific modulation of delta power.

Post hoc analyses largely replicated the spatial patterns observed in the primary model, with minor differences in the extent and statistical strength of effects.

For dopamine, antagonistic profiles were associated with increased delta power at frontal and central regions, whereas agonistic profiles showed increases at occipital regions. In addition, mixed dopaminergic profiles were associated with reduced delta power at temporal sites, indicating a partially extended regional pattern compared to the primary model.

For serotonin, antagonistic profiles were associated with increased delta power at frontal, temporal, and occipital regions, while central effects were not significant. Parietal effects were attenuated and did not survive multiple-comparison correction, indicating a slightly reduced spatial extent compared to the primary model.

For norepinephrine, significant increases were observed at frontal, temporal, and occipital regions across pharmacological profiles, with additional effects at parietal sites for antagonistic profiles. This pattern is consistent with the primary model, although spatial effects appeared more uniformly distributed.

For histamine, antagonistic profiles were associated with robust reductions in delta power across all regions, whereas mixed profiles showed a reduction restricted to central sites. This pattern fully replicated the primary model.

For acetylcholine, antagonistic profiles were associated with increased delta power at frontal, temporal, parietal, and occipital regions, while no significant effect was observed at central sites. Compared to the primary model, this indicates a slightly reduced spatial extent of acetylcholinergic effects.

Detailed results are reported in Supplementary **Table S16**.

### *Results of the simplified mixed-effects model for the theta frequency band*

A significant main effect of electrode location was observed, F(4, 96752) = 23.38, p < .001, indicating stable topographical differences in theta power across frontal, central, temporal, parietal, and occipital regions. In addition, significant main effects were found for all neurotransmitter systems, including dopamine, F(3, 39762) = 6.46, p < .001; serotonin, F(3, 31250) = 17.22, p < .001; norepinephrine, F(3, 31841) = 6.87, p < .001; histamine, F(2, 35983) = 41.97, p< .001; and acetylcholine, F(1, 32919) = 39.97, p < .001.

All neurotransmitter systems showed significant interactions with electrode location—dopamine, F(12, 96757) = 6.87, p < .001; serotonin, F(12, 96763) = 3.55, p < .001; norepinephrine, F(12, 96758) = 3.43, p < .001; histamine, F(8, 96757) = 15.27, p < .001; and acetylcholine, F(4, 96762) = 13.08, p < .001—demonstrating that theta-band modulation is strongly region-dependent.

Post hoc analyses largely replicated the spatial and neurotransmitter-specific patterns observed in the complex model, with minor differences in statistical significance and effect stability, particularly for dopaminergic and noradrenergic systems.

For dopamine, antagonistic profiles were associated with increased theta power at frontal (p = .003), central (p < .001), and parietal (p = .002) regions, whereas effects at temporal (p = 1.000) and occipital (p = 1.000) sites were not significant. Agonistic profiles showed a selective increase at the occipital region (p = .019), with no significant effects at frontal, central, temporal, or parietal regions (all ps ≥ .167). Mixed profiles did not show significant effects at any electrode location (all ps ≥ .060).

This spatially selective pattern is consistent with the complex model, in which dopaminergic effects were primarily interaction-driven and region-specific rather than global.

For serotonin, antagonistic profiles were associated with increased theta power at frontal (p < .001), temporal (p < .001), parietal (p = .002), and occipital (p < .001) regions, while the central effect did not reach significance (p = .071). Agonistic profiles showed a selective increase at the frontal region (p = .044), with no significant effects at central, temporal, parietal, or occipital sites (all ps ≥ .189). Mixed profiles did not show significant effects (all ps ≥ .197).
This pattern closely replicates the complex model, confirming a predominantly cortical increase in theta power with serotonergic antagonism.

For norepinephrine, antagonistic profiles were associated with increased theta power at frontal (p = .003), temporal (p = .007), parietal (p = .002), and occipital (p = .001) regions, whereas the central effect was not significant (p = .217). Agonistic profiles did not show significant effects at any electrode location (all ps ≥ .070). In contrast, mixed profiles were associated with increased theta power at frontal (p < .001), temporal (p < .001), and occipital (p < .001) regions, but not at central or parietal sites.

Notably, compared to the complex model—where norepinephrine showed a more heterogeneous pattern including central decreases—the simplified model revealed a more consistent cortically distributed increase, suggesting improved estimation stability.

For histamine, antagonistic profiles were associated with decreased theta power across all electrode locations: frontal (p < .001), central (p = .004), temporal (p < .001), parietal (p < .001), and occipital (p < .001). Mixed profiles showed a significant decrease only at the central site (p = .038), with no significant effects at frontal, temporal, parietal, or occipital regions.
This highly consistent global suppression replicates the complex model and represents the most robust and spatially uniform effect across all neurotransmitter systems.

For Acetylcholine, antagonistic profiles were associated with increased theta power at frontal (p < .001), temporal (p < .001), parietal (p = .001), and occipital (p < .001) regions, whereas the central effect did not reach significance (p = .085).
This pattern mirrors the complex model and further supports a predominantly cortical enhancement of theta activity under cholinergic antagonism.

Detailed results are reported in Supplementary Table **17**.

### *2.3. Results of the simplified mixed-effects model for the alpha frequency band*

The simplified model confirmed the main findings of the primary analysis. A significant main effect of electrode location was observed, F(4, 96721) = 85.96, p < .001, indicating stable topographical differences in alpha power across frontal, central, temporal, parietal, and occipital regions. Significant main effects were also present for all neurotransmitter systems, including dopamine, F(3, 57690) = 9.05, p < .001; serotonin, F(3, 46917) = 6.92, p < .001; norepinephrine, F(3, 47917) = 4.36, p = .004; histamine, F(2, 53906) = 11.42, p < .001; and acetylcholine, F(1, 50157) = 16.42, p < .001 .

In line with the primary model, significant interactions between neurotransmitter systems and electrode location were observed for dopamine, serotonin, norepinephrine, and histamine (all p < .001), supporting region-specific modulation of alpha power. In contrast, the interaction between acetylcholine and electrode location did not reach significance (F(4, 96728) = 1.85, p = .115), indicating a more spatially uniform effect .

Post hoc analyses largely replicated the spatial patterns observed in the primary model, with minor differences in effect size and spatial extent.

For dopamine, agonistic profiles were associated with significant reductions in alpha power at frontal, central, and temporal regions, while parietal effects were not significant and occipital effects were primarily observed for antagonistic profiles. Mixed dopaminergic profiles showed limited or no significant effects, consistent with the primary model, indicating a predominantly desynchronizing influence of dopaminergic modulation.

For serotonin, antagonistic profiles were associated with increased alpha power at frontal, central, and parietal regions, while temporal effects did not reach significance and occipital effects were not significant. In contrast, agonistic profiles showed significant reductions in occipital regions and additional decreases at temporal sites, alongside selective frontal modulation. This pattern confirms the anterior–posterior dissociation observed in the primary model, although effects appeared more spatially differentiated in the simplified model.

For norepinephrine, antagonistic profiles were associated with reduced alpha power particularly in temporal and occipital regions, while frontal and central effects were not significant and parietal effects were limited. Agonistic profiles showed only minor or non-significant effects across frontal, central, temporal, parietal, and occipital regions. Compared to the primary model, this indicates a slightly more restricted spatial pattern with a clear posterior dominance.

For histamine, antagonistic profiles were associated with robust reductions in alpha power across frontal, central, temporal, parietal, and occipital regions, while mixed profiles showed weaker and more regionally restricted effects. This pattern closely replicates the primary model, confirming a consistent global suppressive effect of histaminergic modulation.

For acetylcholine, antagonistic profiles were associated with increased alpha power across frontal, central, temporal, parietal, and occipital regions. In contrast to other neurotransmitter systems, this effect did not vary significantly across electrode locations, consistent with the absence of a significant interaction term and indicating a spatially uniform modulatory pattern.

Detailed results are reported in Supplementary **Table 18**.

### *2.4. Results of the simplified mixed-effects models for the beta sub-bands (beta-1 and beta-2)*

### *2.4.1. Beta-1 frequency band*

The simplified model for the beta-1 frequency band partially replicated the pattern observed in the complex model. A significant main effect of electrode location was observed, F(4, 96737) = 4.95, p < .001, indicating small but reliable topographical differences in beta-1 power across frontal, central, temporal, parietal, and occipital regions. In addition, a significant main effect of dopamine was found, F(3, 15394) = 2.97, p = .030, whereas serotonin, F(3, 12507) = 0.51, p = .675; norepinephrine, F(3, 12493) = 1.50, p = .213; histamine, F(2, 13534) = 2.51, p = .082; and acetylcholine, F(1, 12429) = 3.23, p = .072, did not reach significance.

In line with the complex model of the overall beta band (prior to sub-band decomposition), no significant interactions between electrode location and any neurotransmitter system were observed (all p > .37), indicating an absence of region-specific modulation of beta-1 power.

Post hoc analyses revealed only isolated effects without a consistent spatial pattern. For dopamine, a single significant effect was observed at temporal sites (agonistic vs. neutral), whereas no consistent effects were found across frontal, central, parietal, or occipital regions. Serotonin, norepinephrine, histamine, and acetylcholine showed no significant effects across any electrode location after multiple-comparison correction.

Overall, the simplified beta-1 model reproduces the absence of robust neurotransmitter-specific modulation observed in the complex model of the overall beta band (prior to sub-band decomposition). The small main effect of dopamine reflects a weak global shift in beta-1 power rather than a spatially structured effect, indicating limited neurophysiological specificity.

Detailed results are reported in Supplementary **Table 19**.

### *2.4.2. Beta-2 frequency band*

The simplified model for the beta-2 frequency band closely mirrored the findings of the complex model of the overall beta band (prior to sub-band decomposition) while providing slightly stronger evidence for dopaminergic effects. A significant main effect of electrode location was observed, F(4, 96700) = 6.28, p < .001, again indicating small topographical differences across frontal, central, temporal, parietal, and occipital regions. A significant main effect of dopamine was also present, F(3, 25181) = 3.00, p = .029. In contrast, serotonin, F(3, 19770) = 0.74, p = .528; norepinephrine, F(3, 19993) = 1.64, p = .177; histamine, F(2, 22269) = 0.77, p = .462; and acetylcholine, F(1, 20265) = 0.64, p = .424, showed no significant main effects.

Consistent with both the complex model and the beta-1 findings, no significant interactions between electrode location and neurotransmitter systems were observed (all p > .43), indicating a lack of region-specific modulation.

Post hoc analyses again revealed only limited and spatially inconsistent effects. For dopamine, a significant effect was observed at frontal sites (antagonistic vs. neutral), while no consistent effects emerged across central, temporal, parietal, or occipital regions. All other neurotransmitter systems (serotonin, norepinephrine, histamine, acetylcholine) showed no significant associations with beta-2 power across any electrode location.

Taken together, the simplified beta-2 model confirms the absence of robust neurotransmitter-specific modulation in the beta range. Although dopaminergic effects reached statistical significance at the global level, these effects were small in magnitude, not spatially organized, and not consistently supported across regions.

Detailed results are reported in Supplementary **Table 20**.

### *2.5. Results of the simplified mixed-effects model for the gamma frequency band*

The simplified model for the gamma frequency band largely replicated the main findings of the complex model of the gamma band, while providing a clearer and more stable pattern of effects. A significant main effect of electrode location was observed, F(4, 96797) = 13.24, p < .001, indicating reliable topographical differences in gamma power across frontal, central, temporal, parietal, and occipital regions. Significant main effects were also found for dopamine, F(3, 17661) = 2.95, p = .032; serotonin, F(3, 14207) = 6.37, p < .001; and norepinephrine, F(3, 14237) = 4.27, p = .005, whereas histamine, F(2, 15529) = 1.20, p = .301, and acetylcholine, F(1, 14217) = 1.14, p = .286, showed no significant effects .

In contrast to the complex model of the gamma band, which showed a significant interaction for dopamine only, the simplified model revealed significant interactions for both dopamine, F(12, 96808) = 3.57, p < .001, and serotonin, F(12, 96820) = 3.20, p < .001, while interactions for norepinephrine, histamine, and acetylcholine were not significant. This pattern suggests a somewhat stronger indication of region-specific modulation for dopaminergic and serotonergic systems in the simplified model compared to the complex model.

Post hoc analyses, however, indicated that these interaction effects were limited in spatial consistency. For dopamine, significant effects were primarily observed at frontal regions, whereas central, temporal, parietal, and occipital regions did not show consistent effects across contrasts. For serotonin, significant increases in gamma power were observed at central, frontal, and parietal regions for antagonistic profiles, with additional effects for higher levels at parietal sites, whereas temporal and occipital regions did not show consistent effects. For norepinephrine, significant effects were observed at central regions, with additional effects at frontal and parietal sites, although these were not supported by significant interaction terms at the model level. Histamine and acetylcholine showed no significant effects across any electrode location.

Detailed results are reported in Supplementary **Table21**.

## Supplementary Tables

***Supplementary Table S1.*** *Dopaminergic receptor coding of psychotropic drugs*

| **Drug Name** | **Drug Group** | **Cat.** | **D1** | **D2** | **D3** | **D4** | **D5** | **DAT** | **Enz. D** | **Rel. D** | **Tot. DA** |
| --- | --- | --- | --- | --- | --- | --- | --- | --- | --- | --- | --- |
| Risperidone | AP | SGA |  | -1 | -1 |  |  |  |  |  | -1 |
| Olanzapine | AP | SGA | -1 | -1 | -1 |  |  |  |  |  | -1 |
| Quetiapine | AP | SGA | -1 | -1 | -1 |  |  |  |  |  | -1 |
| Aripiprazole | AP |  |  | 2 | 2 |  |  |  |  |  | 2 |
| Ziprasidone | AP | SGA | -1 | -1 | -1 |  |  |  |  |  | -1 |
| Haloperidol | AP | FGA | -1 | -1 | -1 |  |  |  |  |  | -1 |
| Clozapin | AP |  | -1 | -1 | -1 |  |  |  |  |  | -1 |
| Escitalopram | AD |  |  |  |  |  |  |  |  |  | 0 |
| Sertraline | AD |  |  |  |  |  |  | 1 |  |  | 1 |
| Paroxetine | AD |  |  |  |  |  |  |  |  |  | 0 |
| Fluoxetine | AD |  |  |  |  |  |  |  |  |  | 0 |
| Bupropion | AD |  |  |  |  |  |  | 1 |  | 1 | 1 |
| Venlafaxine | AD |  |  |  |  |  |  |  |  |  | 0 |
| Mirtazapine | AD |  |  |  |  |  |  |  |  | 1 | 1 |
| Trazodone | AD |  |  |  |  |  |  |  |  |  | 0 |

**Note.** Each medication was coded based on its primary pharmacodynamic actions at dopaminergic receptor subtypes and transporters. Numeric codes indicate receptor-specific actions: +1 = agonist or reuptake inhibitor; −1 = antagonist; 2 = mixed; 0 = neutral. Blank cells indicate no coded effect at the respective receptor or transporter.
The Tot. DA column summarizes the overall dopaminergic effect per drug.
AP = antipsychotic; AD = antidepressant; SGA = second-generation antipsychotic; FGA = first-generation antipsychotic; CLZ = clozapine; Cat. = drug class; Enz. D = enzymatic dopamine modulation; Rel. D = dopamine release modulation.

***Supplementary Table S2. Serotonergic receptor coding of psychotropic drugs***

| Drug Name | Drug Group | Cat. | 5HT1A | 5HT1B | 5HT1D | 5HT2 (unspec.) | 5HT2A | | 5HT2B | 5HT2C | 5HT3 | 5HT6 | 5HT7 | SERT | Enz. SE | Rel. SE | Tot.  5-HT |
| --- | --- | --- | --- | --- | --- | --- | --- | --- | --- | --- | --- | --- | --- | --- | --- | --- | --- |
| Risperidone | AP | SGA |  |  |  |  | -1 |  | |  |  |  |  |  |  |  | -1 |
| Olanzapine | AP | SGA |  |  |  | -1 |  |  | |  |  |  |  |  |  |  | -1 |
| Quetiapine | AP | SGA |  |  |  | -1 |  |  | |  |  |  |  |  |  | 1 | 2 |
| Aripiprazole | AP |  | 2 |  |  |  | -1 |  | |  |  |  |  |  |  |  | 2 |
| Ziprasidone | AP | SGA | 2 | 1 | 2 |  | -1 |  | | -1 |  |  | -1 | 1 |  |  | 2 |
| Haloperidol | AP | FGA |  |  |  |  |  |  | |  |  |  |  |  |  |  | 0 |
| Clozapin | AP |  |  |  |  | -1 |  |  | |  |  |  |  |  |  |  | -1 |
| Escitalopram | AD |  |  |  |  |  |  |  | |  |  |  |  | 1 |  | 1 | 1 |
| Sertraline | AD |  | 1 |  |  |  |  |  | |  |  |  |  | 1 |  | 1 | 1 |
| Paroxetine | AD |  |  |  |  |  |  |  | |  |  |  |  | 1 |  | 1 | 1 |
| Fluoxetine | AD |  |  |  |  |  |  |  | |  |  |  |  | 1 |  | 1 | 1 |
| Bupropion | AD |  |  |  |  |  |  |  | |  |  |  |  |  |  | 1 | 1 |
| Venlafaxine | AD |  |  |  |  |  |  |  | |  |  |  |  | 1 |  | 1 | 1 |
| Mirtazapine | AD |  |  |  |  | -1 |  |  | |  | -1 |  |  |  |  |  | 2 |
| Trazodone | AD |  | 2 |  |  | -1 |  |  | |  |  |  |  | 1 |  | 1 | 2 |

**Note.** Each medication was coded based on its primary pharmacodynamic actions at serotonergic receptor subtypes. Numeric codes indicate receptor-specific actions: +1 = agonist or reuptake inhibitor; −1 = antagonist; 2 = mixed; 0 = neutral. The Total 5-HT column (Tot. 5-HAT) summarizes the overall serotonergic effect per drug. AP = antipsychotic; AD = antidepressant; SGA = second-generation antipsychotic; FGA = first-generation antipsychotic; CLZ = clozapine; Class = drug class; unspec. = unspecified receptor subtype; Enz. SE = enzymatic serotonin modulation; Rel. SE = serotonin release modulati

***Supplementary Table S3. Noradrenergic receptor coding of psychotropic drugs***

| Drug Name | Drug Group | Cat. | α1 | α2 | NET | Enz. NE | Rel. NE | Tot. NE |
| --- | --- | --- | --- | --- | --- | --- | --- | --- |
| Risperidone | AP | SGA | -1 | 1 |  |  |  | 2 |
| Olanzapine | AP | SGA | -1 |  |  |  |  | -1 |
| Quetiapine | AP | SGA | -1 | 1 |  |  | 1 | 2 |
| Aripiprazole | AP |  |  |  |  |  |  | 0 |
| Ziprasidone | AP | SGA | -1 |  | 1 |  |  | 2 |
| Haloperidol | AP | FGA | -1 |  |  |  |  | -1 |
| Clozapin | AP |  | -1 | 1 |  |  |  | 2 |
| Escitalopram | AD |  |  |  |  |  |  | 0 |
| Sertraline | AD |  |  |  |  |  |  | 0 |
| §Paroxetine | AD |  |  |  |  |  |  | 0 |
| Fluoxetine | AD |  |  |  |  |  |  | 0 |
| Bupropion | AD |  |  |  | 1 |  | 1 | 1 |
| Venlafaxine | AD |  |  |  | 1 |  | 1 | 1 |
| Mirtazapine | AD |  |  | 1 |  |  | 1 | 1 |
| Trazodone | AD |  | -1 |  |  |  |  | -1 |

**Note.** Each medication was coded based on its primary pharmacodynamic actions at noradrenergic receptors and transporters. Numeric codes indicate receptor-specific actions: +1 = agonist or reuptake inhibitor; −1 = antagonist; 2 = mixed; 0 = neutral. Blank cells indicate no coded effect at the respective receptor or transporter.
The Tot. NE column summarizes the overall noradrenergic effect per drug.
AP = antipsychotic; AD = antidepressant; SGA = second-generation antipsychotic; FGA = first-generation antipsychotic; CLZ = clozapine; Cat. = drug class; Enz. NE = enzymatic norepinephrine modulation; Rel. NE = norepinephrine release modulation.

***Supplementary Table S4. Histaminergic receptor coding of psychotropic drugs***

| Drug Name | Drug Group | Cat. | H1 | H2 | Rel. H | Tot. HIS |
| --- | --- | --- | --- | --- | --- | --- |
| Risperidone | AP | SGA | -1 |  |  | -1 |
| Olanzapine | AP | SGA | -1 |  |  | -1 |
| Quetiapine | AP | SGA | -1 |  | 1 | 2 |
| Aripiprazole | AP |  |  |  |  | 0 |
| Ziprasidone | AP | SGA |  |  |  | 0 |
| Haloperidol | AP | FGA |  |  |  | 0 |
| Clozapin | AP |  | -1 |  |  | -1 |
| Escitalopram | AD |  |  |  |  | 0 |
| Sertraline | AD |  |  |  |  | 0 |
| Paroxetine | AD |  |  |  |  | 0 |
| Fluoxetine | AD |  |  |  |  | 0 |
| Bupropion | AD |  |  |  |  | 0 |
| Venlafaxine | AD |  |  |  |  | 0 |
| Mirtazapine | AD |  |  |  |  | 0 |
| Trazodone | AD |  |  |  |  | 0 |

**Note.** Each medication was coded based on its primary pharmacodynamic actions at histaminergic receptor subtypes. Numeric codes indicate receptor-specific actions: +1 = agonist or releaser; −1 = antagonist; 2 = mixed; 0 = neutral. The Tot. HIS column summarizes the overall histaminergic effect per drug.
AP = antipsychotic; AD = antidepressant; SGA = second-generation antipsychotic; FGA = first-generation antipsychotic; CLZ = clozapine; Cat. = drug class; Rel. H = histamine release modulation.

***Supplementary Table S5. Cholinergic receptor coding of psychotropic drugs***

| Drug Name | Drug Group | Cat. | M1 | M2 | M3 | M4 | Enz. A | Rel. A | Tot. ACh |
| --- | --- | --- | --- | --- | --- | --- | --- | --- | --- |
| Risperidone | AP | SGA |  |  |  |  |  |  | 0 |
| Olanzapine | AP | SGA | -1 | -1 | -1 | -1 |  |  | -1 |
| Quetiapine | AP | SGA |  |  |  |  |  |  | 0 |
| Aripiprazole | AP |  |  |  |  |  |  |  | 0 |
| Ziprasidone | AP | SGA |  |  |  |  |  |  | 0 |
| Haloperidol | AP | FGA |  |  |  |  |  |  | 0 |
| Clozapin | AP |  | -1 | -1 | -1 | -1 |  |  | -1 |
| Escitalopram | AD |  |  |  |  |  |  |  | 0 |
| Sertraline | AD |  |  |  |  |  |  |  | 0 |
| Paroxetine | AD |  |  |  |  |  |  |  | 0 |
| Fluoxetine | AD |  |  |  |  |  |  |  | 0 |
| Bupropion | AD |  |  |  |  |  |  |  | 0 |
| Venlafaxine | AD |  |  |  |  |  |  |  | 0 |
| Mirtazapine | AD |  |  |  |  |  |  |  | 0 |
| Trazodone | AD |  |  |  |  |  |  |  | 0 |

**Note.** Each medication was coded based on its primary pharmacodynamic actions at cholinergic (muscarinic) receptor subtypes. Numeric codes indicate receptor-specific actions: +1 = agonist or reuptake inhibitor; −1 = antagonist; 2 = mixed or partial agonist; 0 = neutral. Blank cells indicate no coded effect at the respective receptor.
The Tot. ACh column summarizes the overall cholinergic effect per drug.
AP = antipsychotic; AD = antidepressant; SGA = second-generation antipsychotic; FGA = first-generation antipsychotic; CLZ = clozapine; Cat. = drug class; Enz. A = enzymatic acetylcholine modulation; Rel. A = acetylcholine release modulation.

**Supplementary Table S6**. Estimated Marginal Means Contrasts for Delta-Band (1-4 Hz) Power by Neurotransmitter System and Electrode Location

| **Neurotransmitter** | **Electrode Location** | **Contrast** | **Estimate** | **SE** | **z-ratio** | **p** |
| --- | --- | --- | --- | --- | --- | --- |
| Dopamine | Frontal | Antagonist–Neutral | 3.22 | 1.13 | 2.86 | .013* |
|  |  | Agonist–Neutral | 1.59 | 0.98 | 1.63 | .312 |
|  |  | Mixed–Neutral | −0.24 | 1.15 | −0.20 | 1.00 |
|  | Central | Antagonist–Neutral | 4.79 | 1.29 | 3.71 | < .001*** |
|  |  | Agonist–Neutral | 1.32 | 1.09 | 1.21 | .675 |
|  |  | Mixed–Neutral | 2.57 | 1.34 | 1.92 | .164 |
|  | Temporal | Antagonist–Neutral | −0.33 | 1.22 | −0.27 | 1.00 |
|  |  | Agonist–Neutral | 0.92 | 1.04 | 0.88 | 1.00 |
|  |  | Mixed–Neutral | −2.95 | 1.26 | −2.34 | .057 |
|  | Parietal | Antagonist–Neutral | 2.58 | 1.28 | 2.01 | .134 |
|  |  | Agonist–Neutral | 1.47 | 1.09 | 1.36 | .525 |
|  |  | Mixed–Neutral | 0.91 | 1.33 | 0.69 | 1.00 |
|  | Occipital | Antagonist–Neutral | 0.03 | 1.41 | 0.02 | 1.00 |
|  |  | Agonist–Neutral | 2.90 | 1.17 | 2.47 | .040* |
|  |  | Mixed–Neutral | −0.63 | 1.47 | −0.43 | 1.00 |
| Serotonin | Frontal | Antagonist–Neutral | 12.19 | 1.91 | 6.39 | < .001*** |
|  |  | Agonist–Neutral | −0.48 | 1.28 | −0.38 | 1.00 |
|  |  | Mixed–Neutral | −3.17 | 1.34 | −2.36 | .055 |
|  | Central | Antagonist–Neutral | 4.99 | 2.16 | 2.31 | .063 |
|  |  | Agonist–Neutral | −3.13 | 1.43 | −2.19 | .086 |
|  |  | Mixed–Neutral | −2.41 | 1.51 | −1.60 | .329 |
|  | Temporal | Antagonist–Neutral | 11.58 | 2.05 | 5.64 | < .001*** |
|  |  | Agonist–Neutral | −0.32 | 1.37 | −0.23 | 1.00 |
|  |  | Mixed–Neutral | −2.17 | 1.44 | −1.51 | .396 |
|  | Parietal | Antagonist–Neutral | 7.01 | 2.15 | 3.26 | .003** |
|  |  | Agonist–Neutral | −1.96 | 1.43 | −1.37 | .510 |
|  |  | Mixed–Neutral | −2.94 | 1.50 | −1.96 | .151 |
|  | Occipital | Antagonist–Neutral | 9.06 | 2.34 | 3.87 | < .001*** |
|  |  | Agonist–Neutral | −1.74 | 1.54 | −1.13 | .776 |
|  |  | Mixed–Neutral | −4.18 | 1.63 | −2.56 | .031* |
| Norepinephrine | Frontal | Antagonist–Neutral | 5.53 | 1.39 | 3.99 | < .001*** |
|  |  | Agonist–Neutral | 4.03 | 1.13 | 3.56 | .001** |
|  |  | Mixed–Neutral | 7.88 | 2.04 | 3.86 | < .001*** |
|  | Central | Antagonist–Neutral | 1.72 | 1.54 | 1.12 | .794 |
|  |  | Agonist–Neutral | 1.05 | 1.25 | 0.84 | 1.00 |
|  |  | Mixed–Neutral | 2.38 | 2.29 | 1.04 | .897 |
|  | Temporal | Antagonist–Neutral | 5.04 | 1.48 | 3.41 | .002** |
|  |  | Agonist–Neutral | 3.11 | 1.20 | 2.58 | .029* |
|  |  | Mixed–Neutral | 10.09 | 2.18 | 4.63 | < .001*** |
|  | Parietal | Antagonist–Neutral | 3.58 | 1.54 | 2.33 | .060 |
|  |  | Agonist–Neutral | 1.73 | 1.25 | 1.38 | .504 |
|  |  | Mixed–Neutral | 4.83 | 2.28 | 2.12 | .102 |
|  | Occipital | Antagonist–Neutral | 5.26 | 1.66 | 3.16 | .005** |
|  |  | Agonist–Neutral | 2.53 | 1.34 | 1.88 | .181 |
|  |  | Mixed–Neutral | 9.40 | 2.47 | 3.80 | < .001*** |
| Histamine | Frontal | Antagonist–Neutral | −20.13 | 2.36 | −8.51 | < .001*** |
|  |  | Mixed–Neutral | −2.88 | 1.89 | −1.52 | .256 |
|  | Central | Antagonist–Neutral | −13.12 | 2.71 | −4.85 | < .001*** |
|  |  | Mixed–Neutral | −7.41 | 2.16 | −3.43 | .001** |
|  | Temporal | Antagonist–Neutral | −24.17 | 2.56 | −9.42 | < .001*** |
|  |  | Mixed–Neutral | −1.16 | 2.05 | −0.57 | 1.00 |
|  | Parietal | Antagonist–Neutral | −15.48 | 2.70 | −5.74 | < .001*** |
|  |  | Mixed–Neutral | −4.15 | 2.15 | −1.94 | .106 |
|  | Occipital | Antagonist–Neutral | −22.13 | 2.95 | −7.51 | < .001*** |
|  |  | Mixed–Neutral | −2.72 | 2.35 | −1.16 | 1.00 |
| Acetylcholine | Frontal | Antagonist–Neutral | 8.72 | 1.52 | 5.74 | < .001*** |
|  | Central | Antagonist–Neutral | 3.72 | 1.73 | 2.15 | .031* |
|  | Temporal | Antagonist–Neutral | 11.43 | 1.64 | 6.97 | < .001*** |
|  | Parietal | Antagonist–Neutral | 5.81 | 1.72 | 3.37 | .001** |
|  | Occipital | Antagonist–Neutral | 11.09 | 1.87 | 5.92 | < .001*** |

**p < .05; **p < .01; ***p < .001 (Bonferroni-corrected)*

**Supplementary Table S7**. Estimated Marginal Means Contrasts for Theta-Band (4-8 Hz) Power by Neurotransmitter System and Electrode Location

| Neurotransmitter | Electrode  Location | Contrast | Estimate | SE | z | p |
| --- | --- | --- | --- | --- | --- | --- |
| Dopamine | Frontal | Antagonist–Neutral | 1.21 | 0.50 | 2.41 | .048⁎ |
|  |  | Agonist–Neutral | 0.34 | 0.44 | 0.78 | 1.000 |
|  |  | Mixed–Neutral | 0.84 | 0.51 | 1.64 | .302 |
|  | Central | Antagonist–Neutral | 2.39 | 0.57 | 4.19 | < .001⁎⁎⁎ |
|  |  | Agonist–Neutral | 0.35 | 0.49 | 0.71 | 1.000 |
|  |  | Mixed–Neutral | 1.46 | 0.59 | 2.48 | .040⁎ |
|  | Temporal | Antagonist–Neutral | –0.18 | 0.54 | –0.33 | 1.000 |
|  |  | Agonist–Neutral | 0.05 | 0.47 | 0.12 | 1.000 |
|  |  | Mixed–Neutral | –0.04 | 0.56 | –0.08 | 1.000 |
|  | Parietal | Antagonist–Neutral | 1.46 | 0.57 | 2.58 | .030⁎ |
|  |  | Agonist–Neutral | 0.66 | 0.48 | 1.36 | .522 |
|  |  | Mixed–Neutral | 1.04 | 0.59 | 1.77 | .228 |
|  | Occipital | Antagonist–Neutral | –0.28 | 0.62 | –0.45 | 1.000 |
|  |  | Agonist–Neutral | 1.05 | 0.52 | 2.02 | .130 |
|  |  | Mixed–Neutral | 0.47 | 0.65 | 0.72 | 1.000 |
| Serotonin | Frontal | Antagonist–Neutral | 5.75 | 0.85 | 6.75 | < .001⁎⁎⁎ |
|  |  | Agonist–Neutral | 0.96 | 0.58 | 1.65 | .296 |
|  |  | Mixed–Neutral | –0.54 | 0.60 | –0.89 | 1.000 |
|  | Central | Antagonist–Neutral | 3.16 | 0.96 | 3.29 | .003⁎⁎ |
|  |  | Agonist–Neutral | 0.15 | 0.64 | 0.23 | 1.000 |
|  |  | Mixed–Neutral | –0.33 | 0.67 | –0.49 | 1.000 |
|  | Temporal | Antagonist–Neutral | 5.62 | 0.91 | 6.15 | < .001⁎⁎⁎ |
|  |  | Agonist–Neutral | 1.27 | 0.62 | 2.07 | .115 |
|  |  | Mixed–Neutral | –0.24 | 0.64 | –0.38 | 1.000 |
|  | Parietal | Antagonist–Neutral | 3.87 | 0.96 | 4.05 | < .001⁎⁎⁎ |
|  |  | Agonist–Neutral | 0.69 | 0.64 | 1.08 | .847 |
|  |  | Mixed–Neutral | –0.55 | 0.67 | –0.82 | 1.000 |
|  | Occipital | Antagonist–Neutral | 4.97 | 1.04 | 4.80 | < .001⁎⁎⁎ |
|  |  | Agonist–Neutral | 0.53 | 0.69 | 0.76 | 1.000 |
|  |  | Mixed–Neutral | –0.97 | 0.73 | –1.34 | .545 |
| Norepinephrine | Frontal | Antagonist–Neutral | 1.78 | 0.62 | 2.86 | .013⁎ |
|  |  | Agonist–Neutral | –0.67 | 0.51 | –1.33 | .552 |
|  |  | Mixed–Neutral | 3.77 | 0.91 | 4.13 | < .001⁎⁎ |
|  | Central | Antagonist–Neutral | 0.91 | 0.69 | 1.32 | .561 |
|  |  | Agonist–Neutral | –1.49 | 0.56 | –2.67 | .023⁎ |
|  |  | Mixed–Neutral | 1.46 | 1.02 | 1.44 | .452 |
|  | Temporal | Antagonist–Neutral | 1.97 | 0.66 | 2.99 | .008⁎⁎ |
|  |  | Agonist–Neutral | –0.09 | 0.54 | –0.17 | 1.000 |
|  |  | Mixed–Neutral | 4.73 | 0.97 | 4.86 | < .001⁎⁎⁎ |
|  | Parietal | Antagonist–Neutral | 1.79 | 0.69 | 2.60 | .028⁎ |
|  |  | Agonist–Neutral | –0.85 | 0.56 | –1.52 | .384 |
|  |  | Mixed–Neutral | 2.45 | 1.01 | 2.41 | .048⁎ |
|  | Occipital | Antagonist–Neutral | 2.36 | 0.74 | 3.20 | .004⁎⁎ |
|  |  | Agonist–Neutral | –0.34 | 0.60 | –0.57 | 1.000 |
|  |  | Mixed–Neutral | 4.55 | 1.10 | 4.15 | < .001⁎⁎ |
| Histamine | Frontal | Antagonist–Neutral | –8.04 | 1.05 | –7.64 | < .001⁎⁎⁎ |
|  |  | Mixed–Neutral | –2.74 | 0.84 | –3.24 | .002⁎⁎ |
|  | Central | Antagonist–Neutral | –5.57 | 1.20 | –4.64 | < .001⁎⁎⁎ |
|  |  | Mixed–Neutral | –3.53 | 0.96 | –3.69 | .001⁎ |
|  | Temporal | Antagonist–Neutral | –9.54 | 1.14 | –8.38 | < .001⁎⁎⁎ |
|  |  | Mixed–Neutral | –2.08 | 0.91 | –2.29 | .045⁎ |
|  | Parietal | Antagonist–Neutral | –6.31 | 1.20 | –5.28 | < .001⁎⁎⁎ |
|  |  | Mixed–Neutral | –2.47 | 0.95 | –2.59 | .019⁎ |
|  | Occipital | Antagonist–Neutral | –9.22 | 1.30 | –7.08 | < .001⁎⁎⁎ |
|  |  | Mixed–Neutral | –2.40 | 1.04 | –2.31 | .042⁎ |
| Acetylcholine | Frontal | Antagonist–Neutral | 3.42 | 0.68 | 5.05 | < .001⁎⁎⁎ |
|  | Central | Antagonist–Neutral | 1.83 | 0.77 | 2.39 | .017⁎ |
|  | Temporal | Antagonist–Neutral | 5.00 | 0.73 | 6.87 | < .001⁎⁎⁎ |
|  | Parietal | Antagonist–Neutral | 2.71 | 0.76 | 3.55 | < .001⁎⁎⁎ |
|  | Occipital | Antagonist–Neutral | 4.99 | 0.83 | 6.03 | < .001⁎⁎⁎ |

**p < .05; **p < .01; ***p < .001 (Bonferroni-corrected)*

***Supplementary Table S8.*** *Estimated Marginal Means Contrasts for Alpha-Band (8-12 Hz) Power by Neurotransmitter System and Electrode Location*

| Neurotransmitter | Electrode  Location | Contrast | Estimate | SE | z | p |
| --- | --- | --- | --- | --- | --- | --- |
| Dopamine | Frontal | Antagonist–Neutral | -0.31 | 0.27 | -1.13 | 0.777 |
|  |  | Agonist–Neutral | -1.00 | 0.24 | -4.15 | < 0.001*** |
|  |  | Mixed–Neutral | 0.59 | 0.28 | 2.11 | 0.103 |
|  | Central | Antagonist–Neutral | -0.16 | 0.31 | -0.53 | 1.000 |
|  |  | Agonist–Neutral | -1.04 | 0.27 | -3.90 | < 0.001*** |
|  |  | Mixed–Neutral | 0.55 | 0.32 | 1.72 | 0.258 |
|  | Temporal | Antagonist–Neutral | -0.96 | 0.29 | -3.24 | 0.004** |
|  |  | Agonist–Neutral | -1.16 | 0.26 | -4.56 | < 0.001*** |
|  |  | Mixed–Neutral | -0.05 | 0.30 | -0.17 | 1.000 |
|  | Parietal | Antagonist–Neutral | -0.48 | 0.31 | -1.56 | 0.358 |
|  |  | Agonist–Neutral | -0.89 | 0.27 | -3.36 | 0.002** |
|  |  | Mixed–Neutral | 0.37 | 0.32 | 1.15 | 0.754 |
|  | Occipital | Antagonist–Neutral | -1.36 | 0.34 | -4.03 | < 0.001*** |
|  |  | Agonist–Neutral | -0.66 | 0.28 | -2.32 | 0.062 |
|  |  | Mixed–Neutral | 0.56 | 0.35 | 1.59 | 0.333 |
| Serotonin | Frontal | Antagonist–Neutral | 2.27 | 0.47 | 4.83 | < 0.001*** |
|  |  | Agonist–Neutral | 0.92 | 0.32 | 2.86 | 0.013* |
|  |  | Mixed–Neutral | 0.14 | 0.33 | 0.43 | 1.000 |
|  | Central | Antagonist–Neutral | 1.69 | 0.53 | 3.23 | 0.004** |
|  |  | Agonist–Neutral | 0.81 | 0.35 | 2.28 | 0.067 |
|  |  | Mixed–Neutral | 0.17 | 0.37 | 0.46 | 1.000 |
|  | Temporal | Antagonist–Neutral | 1.13 | 0.50 | 2.25 | 0.073 |
|  |  | Agonist–Neutral | 0.06 | 0.34 | 0.19 | 1.000 |
|  |  | Mixed–Neutral | 0.05 | 0.35 | 0.14 | 1.000 |
|  | Parietal | Antagonist–Neutral | 1.26 | 0.52 | 2.40 | 0.049* |
|  |  | Agonist–Neutral | 0.13 | 0.35 | 0.37 | 1.000 |
|  |  | Mixed–Neutral | 0.28 | 0.37 | 0.76 | 1.000 |
|  | Occipital | Antagonist–Neutral | 0.76 | 0.56 | 1.34 | 0.538 |
|  |  | Agonist–Neutral | -1.21 | 0.38 | -3.18 | 0.004** |
|  |  | Mixed–Neutral | 0.34 | 0.40 | 0.85 | 1.000 |
| Norepinephrine | Frontal | Antagonist–Neutral | 0.35 | 0.34 | 1.03 | 0.906 |
|  |  | Agonist–Neutral | 0.19 | 0.28 | 0.70 | 1.000 |
|  |  | Mixed–Neutral | 1.75 | 0.50 | 3.48 | 0.002** |
|  | Central | Antagonist–Neutral | 0.28 | 0.38 | 0.74 | 1.000 |
|  |  | Agonist–Neutral | 0.02 | 0.30 | 0.06 | 1.000 |
|  |  | Mixed–Neutral | 0.87 | 0.56 | 1.55 | 0.360 |
|  | Temporal | Antagonist–Neutral | -0.98 | 0.36 | -2.70 | 0.021* |
|  |  | Agonist–Neutral | 0.60 | 0.29 | 2.03 | 0.126 |
|  |  | Mixed–Neutral | 0.80 | 0.54 | 1.49 | 0.411 |
|  | Parietal | Antagonist–Neutral | -0.53 | 0.38 | -1.39 | 0.492 |
|  |  | Agonist–Neutral | 0.09 | 0.30 | 0.29 | 1.000 |
|  |  | Mixed–Neutral | 0.12 | 0.56 | 0.21 | 1.000 |
|  | Occipital | Antagonist–Neutral | -2.60 | 0.41 | -6.43 | < 0.001*** |
|  |  | Agonist–Neutral | 0.18 | 0.33 | 0.55 | 1.000 |
|  |  | Mixed–Neutral | -0.81 | 0.60 | -1.36 | 0.525 |
| Histamine | Frontal | Antagonist–Neutral | -2.97 | 0.58 | -5.16 | < 0.001*** |
|  |  | Mixed–Neutral | -2.06 | 0.46 | -4.45 | < 0.001*** |
|  | Central | Antagonist–Neutral | -2.06 | 0.65 | -3.15 | 0.003** |
|  |  | Mixed–Neutral | -1.74 | 0.52 | -3.34 | 0.002** |
|  | Temporal | Antagonist–Neutral | -2.66 | 0.62 | -4.28 | < 0.001*** |
|  |  | Mixed–Neutral | -1.16 | 0.50 | -2.34 | 0.039* |
|  | Parietal | Antagonist–Neutral | -1.94 | 0.65 | -2.97 | 0.006** |
|  |  | Mixed–Neutral | -1.44 | 0.52 | -2.76 | 0.012* |
|  | Occipital | Antagonist–Neutral | -2.54 | 0.71 | -3.58 | < 0.001*** |
|  |  | Mixed–Neutral | -1.95 | 0.56 | -3.44 | 0.001** |
| Acetylcholine | Frontal | Antagonist–Neutral | 1.44 | 0.37 | 3.88 | < 0.001*** |
|  | Central | Antagonist–Neutral | 1.13 | 0.42 | 2.71 | 0.007** |
|  | Temporal | Antagonist–Neutral | 1.96 | 0.40 | 4.94 | < 0.001*** |
|  | Parietal | Antagonist–Neutral | 1.33 | 0.42 | 3.19 | 0.001** |
|  | Occipital | Antagonist–Neutral | 1.56 | 0.45 | 3.45 | < 0.001*** |

** p < .05; ** p < .01; *** p < .001 (Bonferroni-corrected)*

***Supplementary Table S9.*** *Estimated Marginal Means Contrasts for Beta-Band (13-30 Hz) Power by Neurotransmitter System and Electrode Location*

| Neurotransmitter | Electrode Location | Contrast | Estimate | SE | z | p |
| --- | --- | --- | --- | --- | --- | --- |
| Dopamine | Frontal | Antagonist - Neutral | 0.25 | 0.11 | 2.25 | 0.074 |
|  |  | Agonist - Neutral | -0.13 | 0.09 | -1.40 | 0.481 |
|  |  | Mixed - Neutral | 0.14 | 0.11 | 1.23 | 0.651 |
|  | Central | Antagonist - Neutral | 0.22 | 0.13 | 1.66 | 0.293 |
|  |  | Agonist - Neutral | -0.20 | 0.11 | -1.86 | 0.189 |
|  |  | Mixed - Neutral | 0.07 | 0.14 | 0.52 | 1.000 |
|  | Temporal | Antagonist - Neutral | 0.07 | 0.12 | 0.61 | 1.000 |
|  |  | Agonist - Neutral | -0.23 | 0.10 | -2.33 | 0.060 |
|  |  | Mixed - Neutral | -0.04 | 0.13 | -0.34 | 1.000 |
|  | Parietal | Antagonist - Neutral | 0.16 | 0.13 | 1.27 | 0.612 |
|  |  | Agonist - Neutral | -0.20 | 0.10 | -1.87 | 0.186 |
|  |  | Mixed - Neutral | 0.05 | 0.14 | 0.40 | 1.000 |
|  | Occipital | Antagonist - Neutral | 0.05 | 0.14 | 0.35 | 1.000 |
|  |  | Agonist - Neutral | -0.18 | 0.12 | -1.58 | 0.343 |
|  |  | Mixed - Neutral | -0.01 | 0.15 | -0.05 | 1.000 |
|  |  |  |  |  |  |  |
| Serotonin | Frontal | Antagonist - Neutral | 0.17 | 0.18 | 0.94 | 1.000 |
|  |  | Agonist - Neutral | 0.08 | 0.11 | 0.73 | 1.000 |
|  |  | Mixed - Neutral | 0.05 | 0.12 | 0.43 | 1.000 |
|  | Central | Antagonist - Neutral | 0.08 | 0.21 | 0.39 | 1.000 |
|  |  | Agonist - Neutral | 0.03 | 0.13 | 0.26 | 1.000 |
|  |  | Mixed - Neutral | 0.10 | 0.15 | 0.69 | 1.000 |
|  | Temporal | Antagonist - Neutral | 0.06 | 0.20 | 0.30 | 1.000 |
|  |  | Agonist - Neutral | 0.08 | 0.12 | 0.62 | 1.000 |
|  |  | Mixed - Neutral | 0.10 | 0.14 | 0.70 | 1.000 |
|  | Parietal | Antagonist - Neutral | 0.03 | 0.21 | 0.12 | 1.000 |
|  |  | Agonist - Neutral | -0.01 | 0.13 | -0.08 | 1.000 |
|  |  | Mixed - Neutral | 0.06 | 0.15 | 0.41 | 1.000 |
|  | Occipital | Antagonist - Neutral | 0.06 | 0.24 | 0.26 | 1.000 |
|  |  | Agonist - Neutral | -0.06 | 0.15 | -0.40 | 1.000 |
|  |  | Mixed - Neutral | 0.08 | 0.16 | 0.52 | 1.000 |
|  |  |  |  |  |  |  |
| Norepinephrine | Frontal | Antagonist - Neutral | -0.08 | 0.13 | -0.66 | 1.000 |
|  |  | Agonist - Neutral | 0.11 | 0.11 | 1.03 | 0.905 |
|  |  | Mixed - Neutral | 0.04 | 0.19 | 0.20 | 1.000 |
|  | Central | Antagonist - Neutral | -0.07 | 0.15 | -0.50 | 1.000 |
|  |  | Agonist - Neutral | 0.05 | 0.12 | 0.41 | 1.000 |
|  |  | Mixed - Neutral | -0.07 | 0.22 | -0.31 | 1.000 |
|  | Temporal | Antagonist - Neutral | -0.14 | 0.14 | -1.00 | 0.953 |
|  |  | Agonist - Neutral | 0.19 | 0.12 | 1.61 | 0.320 |
|  |  | Mixed - Neutral | -0.04 | 0.21 | -0.17 | 1.000 |
|  | Parietal | Antagonist - Neutral | -0.10 | 0.15 | -0.65 | 1.000 |
|  |  | Agonist - Neutral | 0.14 | 0.12 | 1.18 | 0.719 |
|  |  | Mixed - Neutral | -0.09 | 0.22 | -0.39 | 1.000 |
|  | Occipital | Antagonist - Neutral | -0.26 | 0.17 | -1.55 | 0.365 |
|  |  | Agonist - Neutral | 0.12 | 0.13 | 0.86 | 1.000 |
|  |  | Mixed - Neutral | -0.16 | 0.25 | -0.67 | 1.000 |
|  |  |  |  |  |  |  |
| Histamine | Frontal | Antagonist - Neutral | -0.36 | 0.23 | -1.57 | 0.232 |
|  |  | Mixed - Neutral | -0.27 | 0.18 | -1.47 | 0.281 |
|  | Central | Antagonist - Neutral | -0.25 | 0.27 | -0.94 | 0.698 |
|  |  | Mixed - Neutral | -0.31 | 0.21 | -1.45 | 0.294 |
|  | Temporal | Antagonist - Neutral | -0.24 | 0.25 | -0.94 | 0.697 |
|  |  | Mixed - Neutral | -0.07 | 0.20 | -0.35 | 1.000 |
|  | Parietal | Antagonist - Neutral | -0.19 | 0.27 | -0.71 | 0.950 |
|  |  | Mixed - Neutral | -0.24 | 0.21 | -1.13 | 0.516 |
|  | Occipital | Antagonist - Neutral | -0.24 | 0.30 | -0.79 | 0.863 |
|  |  | Mixed - Neutral | -0.18 | 0.24 | -0.74 | 0.917 |
|  |  |  |  |  |  |  |
| Acetylcholine | Frontal | Antagonist - Neutral | 0.10 | 0.14 | 0.71 | 0.476 |
|  | Central | Antagonist - Neutral | 0.03 | 0.17 | 0.20 | 0.844 |
|  | Temporal | Antagonist - Neutral | 0.10 | 0.16 | 0.65 | 0.517 |
|  | Parietal | Antagonist - Neutral | 0.07 | 0.17 | 0.39 | 0.698 |
|  | Occipital | Antagonist - Neutral | 0.12 | 0.19 | 0.61 | 0.541 |

** p < .05; ** p < .01; *** p < .001 (Bonferroni-corrected)*

***Supplementary Table S10.*** *Estimated Marginal Means Contrasts for Gamma-Band (30-100 Hz) Power by Neurotransmitter System and Electrode Location*

| Neurotransmitter | Electrode  Location | Contrast | Estimate | SE | z | p |
| --- | --- | --- | --- | --- | --- | --- |
| Dopamine | Frontal | Antagonist - Neutral | 0.18 | 0.08 | 2.18 | 0.088 |
|  |  | Agonist - Neutral | 0.19 | 0.07 | 2.89 | 0.011* |
|  |  | Mixed - Neutral | -0.04 | 0.09 | -0.45 | 1.000 |
|  | Central | Antagonist - Neutral | 0.07 | 0.10 | 0.72 | 1.000 |
|  |  | Agonist - Neutral | -0.02 | 0.08 | -0.24 | 1.000 |
|  |  | Mixed - Neutral | -0.11 | 0.10 | -1.03 | 0.910 |
|  | Temporal | Antagonist - Neutral | 0.03 | 0.09 | 0.36 | 1.000 |
|  |  | Agonist - Neutral | -0.01 | 0.07 | -0.19 | 1.000 |
|  |  | Mixed - Neutral | -0.18 | 0.10 | -1.91 | 0.170 |
|  | Parietal | Antagonist - Neutral | 0.08 | 0.10 | 0.83 | 1.000 |
|  |  | Agonist - Neutral | -0.04 | 0.08 | -0.46 | 1.000 |
|  |  | Mixed - Neutral | -0.09 | 0.10 | -0.86 | 1.000 |
|  | Occipital | Antagonist - Neutral | 0.12 | 0.11 | 1.07 | 0.855 |
|  |  | Agonist - Neutral | 0.07 | 0.09 | 0.81 | 1.000 |
|  |  | Mixed - Neutral | -0.09 | 0.12 | -0.74 | 1.000 |
|  |  |  |  |  |  |  |
| Serotonin | Frontal | Antagonist - Neutral | 0.19 | 0.13 | 1.44 | 0.449 |
|  |  | Agonist - Neutral | 0.20 | 0.08 | 2.41 | 0.048* |
|  |  | Mixed - Neutral | -0.10 | 0.09 | -1.05 | 0.877 |
|  | Central | Antagonist - Neutral | -0.17 | 0.16 | -1.04 | 0.891 |
|  |  | Agonist - Neutral | 0.12 | 0.10 | 1.23 | 0.659 |
|  |  | Mixed - Neutral | -0.14 | 0.11 | -1.31 | 0.571 |
|  | Temporal | Antagonist - Neutral | -0.12 | 0.15 | -0.81 | 1.000 |
|  |  | Agonist - Neutral | 0.23 | 0.09 | 2.47 | 0.041* |
|  |  | Mixed - Neutral | -0.02 | 0.10 | -0.17 | 1.000 |
|  | Parietal | Antagonist - Neutral | -0.04 | 0.16 | -0.26 | 1.000 |
|  |  | Agonist - Neutral | 0.14 | 0.10 | 1.43 | 0.461 |
|  |  | Mixed - Neutral | -0.09 | 0.11 | -0.83 | 1.000 |
|  | Occipital | Antagonist - Neutral | -0.10 | 0.18 | -0.56 | 1.000 |
|  |  | Agonist - Neutral | 0.24 | 0.11 | 2.12 | 0.101 |
|  |  | Mixed - Neutral | -0.05 | 0.12 | -0.38 | 1.000 |
|  |  |  |  |  |  |  |
| Norepinephrine | Frontal | Antagonist - Neutral | 0.27 | 0.09 | 2.88 | 0.012* |
|  |  | Agonist - Neutral | 0.02 | 0.08 | 0.31 | 1.000 |
|  |  | Mixed - Neutral | 0.32 | 0.14 | 2.26 | 0.072 |
|  | Central | Antagonist - Neutral | 0.29 | 0.11 | 2.62 | 0.026* |
|  |  | Agonist - Neutral | 0.16 | 0.09 | 1.75 | 0.238 |
|  |  | Mixed - Neutral | 0.29 | 0.17 | 1.77 | 0.232 |
|  | Temporal | Antagonist - Neutral | 0.24 | 0.10 | 2.28 | 0.069 |
|  |  | Agonist - Neutral | 0.00 | 0.09 | 0.01 | 1.000 |
|  |  | Mixed - Neutral | 0.23 | 0.15 | 1.48 | 0.416 |
|  | Parietal | Antagonist - Neutral | 0.35 | 0.11 | 3.18 | 0.004** |
|  |  | Agonist - Neutral | 0.10 | 0.09 | 1.05 | 0.883 |
|  |  | Mixed - Neutral | 0.37 | 0.17 | 2.27 | 0.070 |
|  | Occipital | Antagonist - Neutral | 0.16 | 0.12 | 1.26 | 0.620 |
|  |  | Agonist - Neutral | -0.10 | 0.10 | -1.02 | 0.929 |
|  |  | Mixed - Neutral | 0.25 | 0.18 | 1.35 | 0.528 |
|  |  |  |  |  |  |  |
| Histamine | Frontal | Antagonist - Neutral | -0.21 | 0.17 | -1.24 | 0.432 |
|  |  | Mixed - Neutral | -0.06 | 0.13 | -0.43 | 1.000 |
|  | Central | Antagonist - Neutral | -0.06 | 0.20 | -0.31 | 1.000 |
|  |  | Mixed - Neutral | -0.02 | 0.16 | -0.13 | 1.000 |
|  | Temporal | Antagonist - Neutral | -0.02 | 0.19 | -0.10 | 1.000 |
|  |  | Mixed - Neutral | 0.09 | 0.15 | 0.60 | 1.000 |
|  | Parietal | Antagonist - Neutral | -0.17 | 0.20 | -0.86 | 0.779 |
|  |  | Mixed - Neutral | -0.06 | 0.16 | -0.37 | 1.000 |
|  | Occipital | Antagonist - Neutral | -0.13 | 0.23 | -0.56 | 1.000 |
|  |  | Mixed - Neutral | -0.07 | 0.18 | -0.39 | 1.000 |
|  |  |  |  |  |  |  |
| Acetylcholine | Frontal | Antagonist - Neutral | 0.11 | 0.11 | 1.01 | 0.313 |
|  | Central | Antagonist - Neutral | -0.01 | 0.13 | -0.08 | 0.937 |
|  | Temporal | Antagonist - Neutral | -0.06 | 0.12 | -0.48 | 0.628 |
|  | Parietal | Antagonist - Neutral | 0.06 | 0.13 | 0.47 | 0.637 |
|  | Occipital | Antagonist - Neutral | 0.09 | 0.14 | 0.61 | 0.539 |

** p < .05; ** p < .01; *** p < .001 (Bonferroni-corrected)*

***Supplementary Table S11.*** *Bootstrap Estimates, 95% Confidence Intervals, and Bias Values for Region-Specific Contrasts in the Delta Frequency Band* (0.5–4 Hz).

| Neurotransmitter | Electrode Location | Contrast | b | 95% CI |
| --- | --- | --- | --- | --- |
| Dopamine | frontal | Antagonist – Neutral | 3.21 | [0.81, 5.71] |
|  |  | Agonist – Neutral | 1.01 | [−1.35, 3.44] |
|  |  | Mixed – Neutral | 1.42 | [−1.12, 4.06] |
|  | central | Antagonist – Neutral | 4.79 | [2.25, 7.90] |
|  |  | Agonist – Neutral | 1.38 | [−1.09, 3.86] |
|  |  | Mixed – Neutral | 2.58 | [0.32, 5.19] |
|  | temporal | Antagonist – Neutral | 2.52 | [−0.22, 5.11] |
|  |  | Agonist – Neutral | −0.88 | [−3.48, 1.61] |
|  |  | Mixed – Neutral | −2.94 | [−5.28, −0.19] |
|  | parietal | Antagonist – Neutral | 2.01 | [−1.10, 4.85] |
|  |  | Agonist – Neutral | 0.74 | [−1.98, 3.29] |
|  |  | Mixed – Neutral | −0.91 | [−3.52, 1.63] |
|  | occipital | Antagonist – Neutral | 1.89 | [−1.05, 4.66] |
|  |  | Agonist – Neutral | 2.90 | [0.51, 5.28] |
|  |  | Mixed – Neutral | −1.44 | [−4.12, 1.20] |
| Serotonin | frontal | Antagonist – Neutral | 12.11 | [9.00, 15.66] |
|  |  | Agonist – Neutral | 0.94 | [−1.67, 3.41] |
|  |  | Mixed – Neutral | −3.18 | [−5.38, −0.54] |
|  | central | Antagonist – Neutral | 4.99 | [1.20, 9.24] |
|  |  | Agonist – Neutral | −3.12 | [−6.06, −0.34] |
|  |  | Mixed – Neutral | −2.18 | [−5.03, 0.68] |
|  | temporal | Antagonist – Neutral | 11.60 | [8.14, 14.83] |
|  |  | Agonist – Neutral | 0.38 | [−2.37, 3.02] |
|  |  | Mixed – Neutral | −1.21 | [−3.74, 1.35] |
|  | parietal | Antagonist – Neutral | 7.03 | [3.31, 10.27] |
|  |  | Agonist – Neutral | −0.82 | [−3.88, 2.09] |
|  |  | Mixed – Neutral | −1.44 | [−4.32, 1.33] |
|  | occipital | Antagonist – Neutral | 9.07 | [5.06, 12.88] |
|  |  | Agonist – Neutral | −0.75 | [−3.82, 2.12] |
|  |  | Mixed – Neutral | −4.18 | [−6.81, −2.10] |
| Norepinephrine | frontal | Antagonist – Neutral | 5.54 | [3.24, 8.08] |
|  |  | Agonist – Neutral | 4.03 | [2.10, 6.30] |
|  |  | Mixed – Neutral | 7.88 | [4.46, 11.17] |
|  | central | Antagonist – Neutral | 2.01 | [−0.45, 4.40] |
|  |  | Agonist – Neutral | 1.12 | [−1.23, 3.36] |
|  |  | Mixed – Neutral | 2.89 | [−0.02, 5.61] |
|  | temporal | Antagonist – Neutral | 5.04 | [2.19, 7.54] |
|  |  | Agonist – Neutral | 3.10 | [0.57, 5.83] |
|  |  | Mixed – Neutral | 10.11 | [6.21, 13.90] |
|  | parietal | Antagonist – Neutral | 3.58 | [0.16, 6.06] |
|  |  | Agonist – Neutral | 1.44 | [−1.78, 4.22] |
|  |  | Mixed – Neutral | 4.85 | [0.57, 9.25] |
|  | occipital | Antagonist – Neutral | 5.26 | [2.50, 7.81] |
|  |  | Agonist – Neutral | 1.62 | [−1.42, 4.61] |
|  |  | Mixed – Neutral | 9.42 | [4.79, 13.97] |
| Histamine | frontal | Antagonist – Neutral | −20.03 | [−25.21, −15.34] |
|  |  | Mixed – Neutral | −3.12 | [−7.88, 1.34] |
|  | central | Antagonist – Neutral | −13.10 | [−17.65, −9.05] |
|  |  | Mixed – Neutral | −7.41 | [−10.84, −3.78] |
|  | temporal | Antagonist – Neutral | −24.17 | [−29.97, −19.80] |
|  |  | Mixed – Neutral | −2.55 | [−7.44, 1.78] |
|  | parietal | Antagonist – Neutral | −15.48 | [−20.06, −10.54] |
|  |  | Mixed – Neutral | −4.16 | [−7.54, −0.11] |
|  | occipital | Antagonist – Neutral | −22.14 | [−29.20, −16.29] |
|  |  | Mixed – Neutral | −3.08 | [−9.01, 2.22] |
| Acetylcholine | frontal | Antagonist – Neutral | 8.70 | [6.11, 12.27] |
|  | central | Antagonist – Neutral | 3.71 | [−0.11, 7.18] |
|  | temporal | Antagonist – Neutral | 11.42 | [7.96, 14.85] |
|  | parietal | Antagonist – Neutral | 5.80 | [2.55, 9.60] |
|  | occipital | Antagonist – Neutral | 11.08 | [7.21, 14.76] |

***Note****.* *b* = bootstrap estimate. CI = confidence interval. Robust effects are indicated by confidence intervals not including zero.

***Supplementary Table S12.*** *Bootstrap Estimates, 95% Confidence Intervals, and Bias Values for Region-Specific Contrasts in the Theta Frequency Band* (4–8 Hz).

| Neurotransmitter | Electrode Location | Contrast | *b* | 95% CI |
| --- | --- | --- | --- | --- |
| Dopamine | frontal | Antagonist – Neutral | 1.21 | [0.50, 2.25] |
|  |  | Agonist – Neutral | 0.38 | [−1.41, 2.04] |
|  |  | Mixed – Neutral | 0.83 | [0.06, 2.07] |
|  | central | Antagonist – Neutral | 2.40 | [1.33, 3.49] |
|  |  | Agonist – Neutral | 0.55 | [−1.02, 2.11] |
|  |  | Mixed – Neutral | 1.46 | [0.48, 2.69] |
|  | temporal | Antagonist – Neutral | 0.92 | [−0.78, 2.45] |
|  |  | Agonist – Neutral | −0.74 | [−2.31, 0.88] |
|  |  | Mixed – Neutral | −1.22 | [−3.02, 0.42] |
|  | parietal | Antagonist – Neutral | 1.47 | [0.48, 2.52] |
|  |  | Agonist – Neutral | 0.29 | [−1.22, 1.74] |
|  |  | Mixed – Neutral | 1.05 | [0.22, 2.30] |
|  | occipital | Antagonist – Neutral | 0.88 | [−0.64, 2.21] |
|  |  | Agonist – Neutral | 1.05 | [0.24, 2.09] |
|  |  | Mixed – Neutral | −0.61 | [−2.35, 0.95] |
| Serotonin | frontal | Antagonist – Neutral | 5.73 | [3.79, 7.22] |
|  |  | Agonist – Neutral | 0.82 | [−1.35, 2.61] |
|  |  | Mixed – Neutral | −1.44 | [−3.28, 0.42] |
|  | central | Antagonist – Neutral | 3.15 | [0.71, 4.96] |
|  |  | Agonist – Neutral | −1.12 | [−3.22, 0.48] |
|  |  | Mixed – Neutral | −0.98 | [−2.84, 0.88] |
|  | temporal | Antagonist – Neutral | 5.61 | [3.66, 7.21] |
|  |  | Agonist – Neutral | 0.74 | [−1.21, 2.44] |
|  |  | Mixed – Neutral | −0.62 | [−2.48, 1.05] |
|  | parietal | Antagonist – Neutral | 3.87 | [1.88, 5.66] |
|  |  | Agonist – Neutral | 0.41 | [−1.65, 2.08] |
|  |  | Mixed – Neutral | −0.71 | [−2.54, 0.95] |
|  | occipital | Antagonist – Neutral | 4.97 | [3.14, 7.06] |
|  |  | Agonist – Neutral | 0.52 | [−1.58, 2.36] |
|  |  | Mixed – Neutral | −1.88 | [−3.76, 0.12] |
| Norepinephrine | frontal | Antagonist – Neutral | 1.77 | [0.68, 2.89] |
|  |  | Agonist – Neutral | 0.62 | [−1.01, 2.14] |
|  |  | Mixed – Neutral | 3.77 | [1.91, 5.79] |
|  | central | Antagonist – Neutral | 0.91 | [−0.42, 2.30] |
|  |  | Agonist – Neutral | −1.48 | [−2.55, −0.39] |
|  |  | Mixed – Neutral | 1.42 | [−0.38, 3.11] |
|  | temporal | Antagonist – Neutral | 1.97 | [0.75, 3.10] |
|  |  | Agonist – Neutral | 0.44 | [−1.12, 2.03] |
|  |  | Mixed – Neutral | 4.74 | [2.79, 6.78] |
|  | parietal | Antagonist – Neutral | 1.78 | [0.58, 3.04] |
|  |  | Agonist – Neutral | −0.84 | [−1.87, −0.01] |
|  |  | Mixed – Neutral | 2.45 | [0.73, 4.75] |
|  | occipital | Antagonist – Neutral | 2.36 | [0.85, 3.50] |
|  |  | Agonist – Neutral | 0.51 | [−1.21, 2.09] |
|  |  | Mixed – Neutral | 4.56 | [2.06, 6.86] |
| Histamine | frontal | Antagonist – Neutral | −8.02 | [−10.03, −6.34] |
|  |  | Mixed – Neutral | −2.72 | [−4.06, −0.84] |
|  | central | Antagonist – Neutral | −5.56 | [−7.48, −3.62] |
|  |  | Mixed – Neutral | −3.53 | [−5.38, −1.62] |
|  | temporal | Antagonist – Neutral | −9.53 | [−11.65, −7.48] |
|  |  | Mixed – Neutral | −2.08 | [−3.72, −0.60] |
|  | parietal | Antagonist – Neutral | −6.31 | [−8.39, −4.03] |
|  |  | Mixed – Neutral | −2.47 | [−3.95, −0.57] |
|  | occipital | Antagonist – Neutral | −9.21 | [−11.90, −6.73] |
|  |  | Mixed – Neutral | −2.40 | [−3.98, 0.15] |
| Acetylcholine | frontal | Antagonist – Neutral | 3.41 | [2.16, 4.82] |
|  | central | Antagonist – Neutral | 1.82 | [0.04, 3.42] |
|  | temporal | Antagonist – Neutral | 4.99 | [3.56, 6.34] |
|  | parietal | Antagonist – Neutral | 2.71 | [1.07, 4.16] |
|  | occipital | Antagonist – Neutral | 4.99 | [3.53, 6.69] |

**Note.** Estimates (b) and 95% confidence intervals (CI) are based on bootstrap resampling. The central electrode location served as the reference category. Main effects of neurotransmitter systems represent average effects across all electrode locations. Interaction terms (electrode location × neurotransmitter) reflect deviations from the central reference location. Confidence intervals not including zero indicate statistically significant effects.

***Supplementary Table S13.*** *Bootstrap Estimates, 95% Confidence Intervals, and Bias Values for Region-Specific Contrasts in the Alpha Frequency Band* (8–12 Hz).

| Neurotransmitter | Electrode Location | Contrast | b | 95% CI |
| --- | --- | --- | --- | --- |
| Dopamine | **frontal** | Antagonist – Neutral | −0.31 | [−0.69, 0.16] |
|  |  | Agonist – Neutral | −1.00 | [−1.44, −0.53] |
|  |  | Mixed – Neutral | 0.59 | [0.01, 0.99] |
|  | **central** | Antagonist – Neutral | −0.16 | [−0.61, 0.45] |
|  |  | Agonist – Neutral | −1.04 | [−1.55, −0.50] |
|  |  | Mixed – Neutral | 0.55 | [−0.04, 0.99] |
|  | **temporal** | Antagonist – Neutral | −0.95 | [−1.46, −0.37] |
|  |  | Agonist – Neutral | −1.17 | [−1.65, −0.63] |
|  |  | Mixed – Neutral | −0.05 | [−0.61, 0.48] |
|  | **parietal** | Antagonist – Neutral | −0.48 | [−0.96, 0.06] |
|  |  | Agonist – Neutral | −0.89 | [−1.43, −0.43] |
|  |  | Mixed – Neutral | 0.37 | [−0.18, 0.88] |
|  | **occipital** | Antagonist – Neutral | −1.36 | [−1.96, −0.74] |
|  |  | Agonist – Neutral | −0.65 | [−1.19, −0.09] |
|  |  | Mixed – Neutral | 0.56 | [−0.20, 1.26] |
| Serotonin | **frontal** | Antagonist – Neutral | 2.25 | [1.45, 3.15] |
|  |  | Agonist – Neutral | 0.92 | [0.32, 1.44] |
|  |  | Mixed – Neutral | 0.14 | [−0.54, 0.75] |
|  | **central** | Antagonist – Neutral | 1.68 | [0.81, 2.64] |
|  |  | Agonist – Neutral | 0.08 | [-0.73, 0.82] |
|  |  | Mixed – Neutral | −0.20 | [−1.02, 0.63] |
|  | **temporal** | Antagonist – Neutral | 1.10 | [0.23, 2,17] |
|  |  | Agonist – Neutral | 0.17 | [−0.41, 0.74] |
|  |  | Mixed – Neutral | 0.04 | [−0.62, 0.68] |
|  | **parietal** | Antagonist – Neutral | 1.25 | [0.42, 2.28] |
|  |  | Agonist – Neutral | 0.13 | [−0.53, 0.74] |
|  |  | Mixed – Neutral | 0.27 | [−0.53,1.09] |
|  | **occipital** | Antagonist – Neutral | 0.75 | [−0.24, 1.83] |
|  |  | Agonist – Neutral | -1.21 | [−1.99, -0.45] |
|  |  | Mixed – Neutral | 0.33 | [−0.45, 1.11] |
| Norepinephrine | **frontal** | Antagonist – Neutral | 0.35 | [−0.39, 0.99] |
|  |  | Agonist – Neutral | 0.19 | [−0.34, 0.69] |
|  |  | Mixed – Neutral | 1.74 | [0.88, 2.67] |
|  | **central** | Antagonist – Neutral | 0.27 | [−0.26, 1.00] |
|  |  | Agonist – Neutral | 0.02 | [−0.63, 0.60] |
|  |  | Mixed – Neutral | 1.32 | [0.60, 2.04] |
|  | **temporal** | Antagonist – Neutral | −0.38 | [−1.11, 0.38] |
|  |  | Agonist – Neutral | −0.26 | [−0.88, 0.31] |
|  |  | Mixed – Neutral | 0.56 | [−0.42, 1.37] |
|  | **parietal** | Antagonist – Neutral | −0.06 | [−0.63, 0.45] |
|  |  | Agonist – Neutral | 0.16 | [−0.41, 0.73] |
|  |  | Mixed – Neutral | 0.93 | [0.19, 1.68] |
|  | **occipital** | Antagonist – Neutral | 0.52 | [−0.22, 1.29] |
|  |  | Agonist – Neutral | 0.48 | [−0.11, 1.14] |
|  |  | Mixed – Neutral | 2.54 | [1.49, 3.53] |
| Histamine | **frontal** | Antagonist – Neutral | −2.96 | [−3.91, −1.96] |
|  |  | Mixed – Neutral | −2.05 | [−2.83, −1.33] |
|  | **central** | Antagonist – Neutral | −2.05 | [−3.30, −1.02] |
|  |  | Mixed – Neutral | −1.74 | [−2.70, −0.91] |
|  | **temporal** | Antagonist – Neutral | −2.64 | [−3.69, −1.71] |
|  |  | Mixed – Neutral | −0.91 | [−1.70, −0.16] |
|  | **parietal** | Antagonist – Neutral | −1.75 | [−2.69, −0.86] |
|  |  | Mixed – Neutral | −0.71 | [−1.39, 0.02] |
|  | **occipital** | Antagonist – Neutral | −1.36 | [−2.39, −0.35] |
|  |  | Mixed – Neutral | −1.70 | [−2.52, −0.88] |
| Acetylcholine | **frontal** | Antagonist – Neutral | 1.43 | [0.61, 2.17] |
|  | **central** | Antagonist – Neutral | 1.12 | [0.38, 1.98] |
|  | **temporal** | Antagonist – Neutral | 1.95 | [1.10, 2.76] |
|  | **parietal** | Antagonist – Neutral | 1.32 | [0.52, 2.18] |
|  | **occipital** | Antagonist – Neutral | 1.55 | [0.78, 2.52] |

**Note.** Estimates (b) and 95% confidence intervals (CI) are based on bootstrap resampling. The central electrode location served as the reference category. Main effects of neurotransmitter systems represent average effects across all electrode locations. Interaction terms (electrode location × neurotransmitter) reflect deviations from the central reference location. Confidence intervals not including zero indicate statistically significant effects.

***Supplementary Table S14.*** *Bootstrap Estimates, 95% Confidence Intervals, and Bias Values for Region-Specific Contrasts in the Beta Frequency Band* (13–30 Hz).

| Neurotransmitter | Electrode Location | Contrast | b | 95% CI |
| --- | --- | --- | --- | --- |
| Dopamine | frontal | Antagonist – Neutral | 0.24 | [0.00, 0.44] |
|  |  | Agonist – Neutral | -0.13 | [-0.29, 0.02] |
|  |  | Mixed – Neutral | 0.14 | [-0.09, 0.37] |
|  | central | Antagonist – Neutral | 0.22 | [-0.05, 0.41] |
|  |  | Agonist – Neutral | -0.20 | [-0.42, 0.03] |
|  |  | Mixed – Neutral | 0.07 | [-0.22, 0.35] |
|  | temporal | Antagonist – Neutral | 0.07 | [-0.21, 0.32] |
|  |  | Agonist – Neutral | -0.23 | [-0.42, -0.06] |
|  |  | Mixed – Neutral | -0.04 | [-0.33, 0.16] |
|  | parietal | Antagonist – Neutral | 0.16 | [-0.13, 0.45] |
|  |  | Agonist – Neutral | -0.20 | [-0.38, 0.01] |
|  |  | Mixed – Neutral | 0.06 | [-0.24, 0.41] |
|  | occipital | Antagonist – Neutral | 0.05 | [-0.21, 0.28] |
|  |  | Agonist – Neutral | -0.18 | [-0.38, 0.03] |
|  |  | Mixed – Neutral | -0.01 | [-0.28, 0.19] |
| Serotonin | frontal | Antagonist – Neutral | 0.17 | [-0.14, 0.53] |
|  |  | Agonist – Neutral | 0.08 | [-0.13, 0.31] |
|  |  | Mixed – Neutral | 0.05 | [-0.17, 0.31] |
|  | central | Antagonist – Neutral | 0.08 | [-0.35, 0.41] |
|  |  | Agonist – Neutral | 0.03 | [-0.18, 0.32] |
|  |  | Mixed – Neutral | 0.10 | [-0.16, 0.40] |
|  | temporal | Antagonist – Neutral | 0.06 | [-0.41, 0.43] |
|  |  | Agonist – Neutral | 0.08 | [-0.19, 0.28] |
|  |  | Mixed – Neutral | 0.10 | [-0.13, 0.39] |
|  | parietal | Antagonist – Neutral | 0.03 | [-0.50, 0.42] |
|  |  | Agonist – Neutral | -0.01 | [-0.22, 0.23] |
|  |  | Mixed – Neutral | 0.06 | [-0.22, 0.33] |
|  | occipital | Antagonist – Neutral | 0.06 | [-0.40, 0.60] |
|  |  | Agonist – Neutral | -0.06 | [-0.35, 0.25] |
|  |  | Mixed – Neutral | 0.08 | [-0.25, 0.42] |
| Norepinephrine | frontal | Antagonist – Neutral | -0.08 | [-0.31, 0.15] |
|  |  | Agonist – Neutral | 0.11 | [-0.09, 0.27] |
|  |  | Mixed – Neutral | 0.04 | [-0.34, 0.31] |
|  | central | Antagonist – Neutral | -0.07 | [-0.35, 0.22] |
|  |  | Agonist – Neutral | 0.05 | [-0.18, 0.27] |
|  |  | Mixed – Neutral | -0.07 | [-0.48, 0.26] |
|  | temporal | Antagonist – Neutral | -0.14 | [-0.44, 0.11] |
|  |  | Agonist – Neutral | 0.19 | [-0.05, 0.38] |
|  |  | Mixed – Neutral | -0.04 | [-0.48, 0.33] |
|  | parietal | Antagonist – Neutral | -0.10 | [-0.38, 0.18] |
|  |  | Agonist – Neutral | 0.14 | [-0.09, 0.36] |
|  |  | Mixed – Neutral | -0.08 | [-0.53, 0.35] |
|  | occipital | Antagonist – Neutral | -0.26 | [-0.57, 0.00] |
|  |  | Agonist – Neutral | 0.12 | [-0.14, 0.39] |
|  |  | Mixed – Neutral | -0.16 | [-0.64, 0.32] |
| Histamine | frontal | Antagonist – Neutral | -0.35 | [-0.78, 0.03] |
|  |  | Mixed – Neutral | -0.26 | [-0.59, 0.05] |
|  | central | Antagonist – Neutral | -0.25 | [-0.78, 0.23] |
|  |  | Mixed – Neutral | -0.31 | [-0.73, 0.11] |
|  | temporal | Antagonist – Neutral | -0.24 | [-0.75, 0.18] |
|  |  | Mixed – Neutral | -0.07 | [-0.47, 0.25] |
|  | parietal | Antagonist – Neutral | -0.19 | [-0.70, 0.24] |
|  |  | Mixed – Neutral | -0.24 | [-0.63, 0.12] |
|  | occipital | Antagonist – Neutral | -0.24 | [-0.82, 0.34] |
|  |  | Mixed – Neutral | -0.18 | [-0.70, 0.24] |
| Acetylcholine | frontal | Antagonist – Neutral | 0.10 | [-0.14, 0.32] |
|  | central | Antagonist – Neutral | 0.03 | [-0.30, 0.33] |
|  | temporal | Antagonist – Neutral | 0.10 | [-0.20, 0.37] |
|  | parietal | Antagonist – Neutral | 0.07 | [-0.28, 0.37] |
|  | occipital | Antagonist – Neutral | 0.12 | [-0.28, 0.44] |

**Note.** Estimates (b) and 95% confidence intervals (CI) are based on bootstrap resampling. The central electrode location served as the reference category. Main effects of neurotransmitter systems represent average effects across all electrode locations. Interaction terms (electrode location × neurotransmitter) reflect deviations from the central reference location. Confidence intervals not including zero indicate statistically significant effects.

***Supplementary Table S15.*** *Bootstrap Estimates, 95% Confidence Intervals, and Bias Values for Region-Specific Contrasts in the Gamma Frequency Band* (30–100 Hz)

| Neurotransmitter | Electrode Location | Contrast | b | 95% CI |
| --- | --- | --- | --- | --- |
| Dopamine | frontal | Antagonist – Neutral | 0.18 | [-0.01, 0.33] |
|  |  | Agonist – Neutral | 0.19 | [0.06, 0.31] |
|  |  | Mixed – Neutral | -0.04 | [-0.22, 0.12] |
|  | central | Antagonist – Neutral | 0.07 | [-0.12, 0.25] |
|  |  | Agonist – Neutral | -0.02 | [-0.18, 0.11] |
|  |  | Mixed – Neutral | -0.11 | [-0.33, 0.09] |
|  | temporal | Antagonist – Neutral | 0.03 | [-0.17, 0.21] |
|  |  | Agonist – Neutral | -0.01 | [-0.18, 0.12] |
|  |  | Mixed – Neutral | -0.18 | [-0.41, -0.01] |
|  | parietal | Antagonist – Neutral | 0.08 | [-0.07, 0.25] |
|  |  | Agonist – Neutral | -0.04 | [-0.24, 0.13] |
|  |  | Mixed – Neutral | -0.09 | [-0.30, 0.11] |
|  | occipital | Antagonist – Neutral | 0.12 | [-0.14, 0.34] |
|  |  | Agonist – Neutral | 0.07 | [-0.14, 0.20] |
|  |  | Mixed – Neutral | -0.09 | [-0.30, 0.16] |
| Serotonin | frontal | Antagonist – Neutral | 0.19 | [-0.04, 0.44] |
|  |  | Agonist – Neutral | 0.20 | [0.03, 0.38] |
|  |  | Mixed – Neutral | -0.10 | [-0.25, 0.08] |
|  | central | Antagonist – Neutral | -0.16 | [-0.45, 0.12] |
|  |  | Agonist – Neutral | 0.12 | [-0.10, 0.29] |
|  |  | Mixed – Neutral | -0.14 | [-0.36, 0.07] |
|  | temporal | Antagonist – Neutral | -0.12 | [-0.37, 0.10] |
|  |  | Agonist – Neutral | 0.23 | [0.06, 0.36] |
|  |  | Mixed – Neutral | -0.02 | [-0.18, 0.16] |
|  | parietal | Antagonist – Neutral | -0.04 | [-0.34, 0.22] |
|  |  | Agonist – Neutral | 0.14 | [-0.07, 0.32] |
|  |  | Mixed – Neutral | -0.09 | [-0.28, 0.09] |
|  | occipital | Antagonist – Neutral | -0.10 | [-0.38, 0.27] |
|  |  | Agonist – Neutral | 0.24 | [0.01, 0.39] |
|  |  | Mixed – Neutral | -0.05 | [-0.26, 0.16] |
| Norepinephrine | frontal | Antagonist – Neutral | 0.27 | [0.08, 0.49] |
|  |  | Agonist – Neutral | 0.03 | [-0.16, 0.17] |
|  |  | Mixed – Neutral | 0.31 | [0.07, 0.54] |
|  | central | Antagonist – Neutral | 0.29 | [0.08, 0.51] |
|  |  | Agonist – Neutral | 0.16 | [-0.02, 0.33] |
|  |  | Mixed – Neutral | 0.29 | [-0.04, 0.58] |
|  | temporal | Antagonist – Neutral | 0.24 | [-0.01, 0.44] |
|  |  | Agonist – Neutral | 0.00 | [-0.15, 0.13] |
|  |  | Mixed – Neutral | 0.23 | [-0.09, 0.50] |
|  | parietal | Antagonist – Neutral | 0.35 | [0.15, 0.59] |
|  |  | Agonist – Neutral | 0.10 | [-0.09, 0.24] |
|  |  | Mixed – Neutral | 0.37 | [0.13, 0.69] |
|  | occipital | Antagonist – Neutral | 0.16 | [-0.14, 0.47] |
|  |  | Agonist – Neutral | -0.10 | [-0.28, 0.08] |
|  |  | Mixed – Neutral | 0.25 | [-0.16, 0.69] |
| Histamine | frontal | Antagonist – Neutral | -0.20 | [-0.56, 0.10] |
|  |  | Mixed – Neutral | -0.05 | [-0.37, 0.23] |
|  | central | Antagonist – Neutral | -0.06 | [-0.46, 0.31] |
|  |  | Mixed – Neutral | -0.02 | [-0.35, 0.27] |
|  | temporal | Antagonist – Neutral | -0.02 | [-0.35, 0.31] |
|  |  | Mixed – Neutral | 0.09 | [-0.13, 0.35] |
|  | parietal | Antagonist – Neutral | -0.17 | [-0.57, 0.19] |
|  |  | Mixed – Neutral | -0.06 | [-0.39, 0.22] |
|  | occipital | Antagonist – Neutral | -0.13 | [-0.58, 0.20] |
|  |  | Mixed – Neutral | -0.07 | [-0.42, 0.24] |
| Acetylcholine | frontal | Antagonist – Neutral | 0.11 | [-0.09, 0.35] |
|  | central | Antagonist – Neutral | -0.01 | [-0.25, 0.26] |
|  | temporal | Antagonist – Neutral | -0.06 | [-0.23, 0.16] |
|  | parietal | Antagonist – Neutral | 0.06 | [-0.14, 0.36] |
|  | occipital | Antagonist – Neutral | 0.09 | [-0.12, 0.35] |

**Note.** Estimates (b) and 95% confidence intervals (CI) are based on bootstrap resampling. The central electrode location served as the reference category. Main effects of neurotransmitter systems represent average effects across all electrode locations. Interaction terms (electrode location × neurotransmitter) reflect deviations from the central reference location. Confidence intervals not including zero indicate statistically significant effects.

***Supplementary Table S16. Simplified mixed-effects model.*** *Estimated Marginal Means Contrasts for Delta-Band Power by Neurotransmitter System and Electrode Location (mixed-effects model with patient-level random intercept only).*

| \| **Neurotransmitter** \| **Electrode Location** \| **Contrast** \| **Estimate** \| **SE** \| **z-ratio** \| **p** \| \| --- \| --- \| --- \| --- \| --- \| --- \| --- \| \| Dopamine \| Frontal \| Antagonist–neutral \| 0.039 \| 0.008 \| 4.81 \| < .001*** \| \|  \|  \| Agonist–neutral \| 0.012 \| 0.007 \| 1.77 \| .229 \| \|  \|  \| Mixed–neutral \| −0.006 \| 0.009 \| −0.73 \| 1.000 \| \|  \| Central \| Antagonist–neutral \| 0.059 \| 0.010 \| 6.00 \| < .001*** \| \|  \|  \| Agonist–neutral \| 0.010 \| 0.008 \| 1.24 \| .644 \| \|  \|  \| Mixed–neutral \| 0.025 \| 0.011 \| 2.40 \| .049* \| \|  \| Temporal \| Antagonist–neutral \| 0.000 \| 0.009 \| 0.05 \| 1.000 \| \|  \|  \| Agonist–neutral \| 0.007 \| 0.008 \| 0.94 \| 1.000 \| \|  \|  \| Mixed–neutral \| −0.034 \| 0.010 \| −3.49 \| .002** \| \|  \| Parietal \| Antagonist–neutral \| 0.032 \| 0.010 \| 3.33 \| .003** \| \|  \|  \| Agonist–neutral \| 0.012 \| 0.008 \| 1.51 \| .391 \| \|  \|  \| Mixed–neutral \| 0.005 \| 0.011 \| 0.47 \| 1.000 \| \|  \| Occipital \| Antagonist–neutral \| 0.006 \| 0.011 \| 0.57 \| 1.000 \| \|  \|  \| Agonist–neutral \| 0.026 \| 0.009 \| 2.95 \| .010** \| \|  \|  \| Mixed–neutral \| −0.011 \| 0.012 \| −0.90 \| 1.000 \| \| Serotonin \| Frontal \| Antagonist–neutral \| 0.077 \| 0.014 \| 5.49 \| < .001*** \| \|  \|  \| Agonist–neutral \| 0.006 \| 0.005 \| 1.18 \| .719 \| \|  \|  \| Mixed–neutral \| −0.025 \| 0.009 \| −2.84 \| .014** \| \|  \| Central \| Antagonist–neutral \| 0.016 \| 0.017 \| 0.97 \| 1.000 \| \|  \|  \| Agonist–neutral \| −0.013 \| 0.006 \| −2.26 \| .072 \| \|  \|  \| Mixed–neutral \| −0.008 \| 0.010 \| −0.78 \| 1.000 \| \|  \| Temporal \| Antagonist–neutral \| 0.078 \| 0.016 \| 5.04 \| < .001*** \| \|  \|  \| Agonist–neutral \| 0.003 \| 0.005 \| 0.48 \| 1.000 \| \|  \|  \| Mixed–neutral \| −0.016 \| 0.010 \| −1.72 \| .255 \| \|  \| Parietal \| Antagonist–neutral \| 0.039 \| 0.017 \| 2.34 \| .059 \| \|  \|  \| Agonist–neutral \| −0.006 \| 0.006 \| −1.09 \| .831 \| \|  \|  \| Mixed–neutral \| −0.015 \| 0.010 \| −1.46 \| .431 \| \|  \| Occipital \| Antagonist–neutral \| 0.062 \| 0.018 \| 3.37 \| .002** \| \|  \|  \| Agonist–neutral \| −0.003 \| 0.007 \| −0.48 \| 1.000 \| \|  \|  \| Mixed–neutral \| −0.027 \| 0.011 \| −2.34 \| .058 \| \| Norepinephrine \| Frontal \| Antagonist–neutral \| 0.050 \| 0.009 \| 5.49 \| < .001*** \| \|  \|  \| Agonist–neutral \| 0.033 \| 0.008 \| 4.08 \| < .001*** \| \|  \|  \| Mixed–neutral \| 0.066 \| 0.014 \| 4.68 \| < .001*** \| \|  \| Central \| Antagonist–neutral \| 0.018 \| 0.011 \| 1.65 \| .297 \| \|  \|  \| Agonist–neutral \| 0.005 \| 0.009 \| 0.55 \| 1.000 \| \|  \|  \| Mixed–neutral \| 0.007 \| 0.017 \| 0.45 \| 1.000 \| \|  \| Temporal \| Antagonist–neutral \| 0.045 \| 0.010 \| 4.47 \| < .001*** \| \|  \|  \| Agonist–neutral \| 0.027 \| 0.009 \| 3.03 \| .007** \| \|  \|  \| Mixed–neutral \| 0.079 \| 0.015 \| 5.09 \| < .001*** \| \|  \| Parietal \| Antagonist–neutral \| 0.034 \| 0.011 \| 3.24 \| .004** \| \|  \|  \| Agonist–neutral \| 0.013 \| 0.009 \| 1.41 \| .480 \| \|  \|  \| Mixed–neutral \| 0.031 \| 0.017 \| 1.88 \| .179 \| \|  \| Occipital \| Antagonist–neutral \| 0.050 \| 0.012 \| 4.20 \| < .001*** \| \|  \|  \| Agonist–neutral \| 0.023 \| 0.010 \| 2.19 \| .084 \| \|  \|  \| Mixed–neutral \| 0.076 \| 0.018 \| 4.08 \| < .001*** \| \| Histamine \| Frontal \| Antagonist–neutral \| −0.139 \| 0.017 \| −8.05 \| < .001*** \| \|  \|  \| Mixed–neutral \| −0.021 \| 0.013 \| −1.58 \| .226 \| \|  \| Central \| Antagonist–neutral \| −0.066 \| 0.021 \| −3.17 \| .003** \| \|  \|  \| Mixed–neutral \| −0.062 \| 0.016 \| −3.86 \| < .001*** \| \|  \| Temporal \| Antagonist–neutral \| −0.174 \| 0.019 \| −8.99 \| < .001*** \| \|  \|  \| Mixed–neutral \| 0.017 \| 0.015 \| 1.10 \| .545 \| \|  \| Parietal \| Antagonist–neutral \| −0.088 \| 0.021 \| −4.27 \| < .001*** \| \|  \|  \| Mixed–neutral \| −0.023 \| 0.016 \| −1.43 \| .304 \| \|  \| Occipital \| Antagonist–neutral \| −0.156 \| 0.023 \| −6.74 \| < .001*** \| \|  \|  \| Mixed–neutral \| −0.004 \| 0.018 \| −0.23 \| 1.000 \| \| Acetylcholine \| Frontal \| Antagonist–neutral \| 0.072 \| 0.011 \| 6.29 \| < .001*** \| \|  \| Central \| Antagonist–neutral \| 0.022 \| 0.014 \| 1.59 \| .112 \| \|  \| Temporal \| Antagonist–neutral \| 0.106 \| 0.013 \| 8.27 \| < .001*** \| \|  \| Parietal \| Antagonist–neutral \| 0.043 \| 0.014 \| 3.17 \| .002** \| \|  \| Occipital \| Antagonist–neutral \| 0.100 \| 0.015 \| 6.63 \| < .001*** \| |
| --- | --- | --- | --- | --- | --- | --- | --- | --- | --- | --- | --- | --- | --- | --- | --- | --- | --- | --- | --- | --- | --- | --- | --- | --- | --- | --- | --- | --- | --- | --- | --- | --- | --- | --- | --- | --- | --- | --- | --- | --- | --- | --- | --- | --- | --- | --- | --- | --- | --- | --- | --- | --- | --- | --- | --- | --- | --- | --- | --- | --- | --- | --- | --- | --- | --- | --- | --- | --- | --- | --- | --- | --- | --- | --- | --- | --- | --- | --- | --- | --- | --- | --- | --- | --- | --- | --- | --- | --- | --- | --- | --- | --- | --- | --- | --- | --- | --- | --- | --- | --- | --- | --- | --- | --- | --- | --- | --- | --- | --- | --- | --- | --- | --- | --- | --- | --- | --- | --- | --- | --- | --- | --- | --- | --- | --- | --- | --- | --- | --- | --- | --- | --- | --- | --- | --- | --- | --- | --- | --- | --- | --- | --- | --- | --- | --- | --- | --- | --- | --- | --- | --- | --- | --- | --- | --- | --- | --- | --- | --- | --- | --- | --- | --- | --- | --- | --- | --- | --- | --- | --- | --- | --- | --- | --- | --- | --- | --- | --- | --- | --- | --- | --- | --- | --- | --- | --- | --- | --- | --- | --- | --- | --- | --- | --- | --- | --- | --- | --- | --- | --- | --- | --- | --- | --- | --- | --- | --- | --- | --- | --- | --- | --- | --- | --- | --- | --- | --- | --- | --- | --- | --- | --- | --- | --- | --- | --- | --- | --- | --- | --- | --- | --- | --- | --- | --- | --- | --- | --- | --- | --- | --- | --- | --- | --- | --- | --- | --- | --- | --- | --- | --- | --- | --- | --- | --- | --- | --- | --- | --- | --- | --- | --- | --- | --- | --- | --- | --- | --- | --- | --- | --- | --- | --- | --- | --- | --- | --- | --- | --- | --- | --- | --- | --- | --- | --- | --- | --- | --- | --- | --- | --- | --- | --- | --- | --- | --- | --- | --- | --- | --- | --- | --- | --- | --- | --- | --- | --- | --- | --- | --- | --- | --- | --- | --- | --- | --- | --- | --- | --- | --- | --- | --- | --- | --- | --- | --- | --- | --- | --- | --- | --- | --- | --- | --- | --- | --- | --- | --- | --- | --- | --- | --- | --- | --- | --- | --- | --- | --- | --- | --- | --- | --- | --- | --- | --- | --- | --- | --- | --- | --- | --- | --- | --- | --- | --- | --- | --- | --- | --- | --- | --- | --- | --- | --- | --- | --- | --- | --- | --- | --- | --- | --- | --- | --- | --- | --- | --- | --- | --- | --- | --- | --- | --- | --- | --- | --- | --- | --- | --- | --- | --- | --- | --- | --- | --- | --- | --- | --- | --- | --- | --- | --- | --- | --- | --- | --- | --- | --- | --- | --- | --- | --- | --- | --- | --- | --- | --- |

**p < .05; **p < .01; ***p < .001 (Bonferroni-corrected)*

***Supplementary Table S17. Simplified mixed-effects model. Estimated Marginal Means Contrasts for Theta-Band Power by Neurotransmitter System and Electrode Location (mixed-effects model with patient-level random intercept only).***

| Neurotransmitter | Electrode | Contrast | Estimate | SE | t | p |
| --- | --- | --- | --- | --- | --- | --- |
| **Dopamine** | Frontal | Antagonist–Neutral | 0.015 | 0.004 | 4.21 | < .001*** |
|  |  | Agonist–Neutral | 0.002 | 0.003 | 0.81 | 1.000 |
|  |  | Mixed–Neutral | 0.002 | 0.004 | 0.60 | 1.000 |
|  | Central | Antagonist–Neutral | 0.030 | 0.004 | 6.97 | < .001*** |
|  |  | Agonist–Neutral | 0.003 | 0.004 | 0.98 | .980 |
|  |  | Mixed–Neutral | 0.011 | 0.005 | 2.33 | .060 |
|  | Temporal | Antagonist–Neutral | −0.000 | 0.004 | −0.10 | 1.000 |
|  |  | Agonist–Neutral | 0.000 | 0.003 | 0.10 | 1.000 |
|  |  | Mixed–Neutral | −0.007 | 0.004 | −1.56 | .354 |
|  | Parietal | Antagonist–Neutral | 0.018 | 0.004 | 4.25 | < .001*** |
|  |  | Agonist–Neutral | 0.007 | 0.004 | 1.92 | .167 |
|  |  | Mixed–Neutral | 0.005 | 0.005 | 1.05 | .877 |
|  | Occipital | Antagonist–Neutral | 0.001 | 0.005 | 0.19 | 1.000 |
|  |  | Agonist–Neutral | 0.011 | 0.004 | 2.74 | .019* |
|  |  | Mixed–Neutral | −0.001 | 0.005 | −0.12 | 1.000 |
| **Serotonin** | Frontal | Antagonist–Neutral | 0.040 | 0.006 | 6.48 | < .001*** |
|  |  | Agonist–Neutral | 0.005 | 0.002 | 2.44 | .044* |
|  |  | Mixed–Neutral | −0.006 | 0.004 | −1.53 | .381 |
|  | Central | Antagonist–Neutral | 0.016 | 0.007 | 2.26 | .071 |
|  |  | Agonist–Neutral | 0.002 | 0.003 | 0.87 | 1.000 |
|  |  | Mixed–Neutral | 0.000 | 0.004 | 0.04 | 1.000 |
|  | Temporal | Antagonist–Neutral | 0.038 | 0.007 | 5.63 | < .001*** |
|  |  | Agonist–Neutral | 0.004 | 0.002 | 1.86 | .189 |
|  |  | Mixed–Neutral | −0.005 | 0.004 | −1.24 | .642 |
|  | Parietal | Antagonist–Neutral | 0.024 | 0.007 | 3.35 | .002** |
|  |  | Agonist–Neutral | 0.004 | 0.003 | 1.70 | .269 |
|  |  | Mixed–Neutral | −0.003 | 0.004 | −0.78 | 1.000 |
|  | Occipital | Antagonist–Neutral | 0.034 | 0.008 | 4.29 | < .001*** |
|  |  | Agonist–Neutral | 0.001 | 0.003 | 0.44 | 1.000 |
|  |  | Mixed–Neutral | −0.009 | 0.005 | −1.84 | .197 |
| **Norepinephrine** | Frontal | Antagonist–Neutral | 0.013 | 0.004 | 3.33 | .003** |
|  |  | Agonist–Neutral | −0.002 | 0.004 | −0.68 | 1.000 |
|  |  | Mixed–Neutral | 0.023 | 0.006 | 3.81 | < .001*** |
|  | Central | Antagonist–Neutral | 0.008 | 0.005 | 1.80 | .217 |
|  |  | Agonist–Neutral | −0.009 | 0.004 | −2.27 | .070 |
|  |  | Mixed–Neutral | 0.003 | 0.007 | 0.38 | 1.000 |
|  | Temporal | Antagonist–Neutral | 0.013 | 0.004 | 3.05 | .007** |
|  |  | Agonist–Neutral | 0.004 | 0.004 | 0.96 | 1.000 |
|  |  | Mixed–Neutral | 0.032 | 0.007 | 4.70 | < .001*** |
|  | Parietal | Antagonist–Neutral | 0.016 | 0.005 | 3.36 | .002** |
|  |  | Agonist–Neutral | −0.003 | 0.004 | −0.66 | 1.000 |
|  |  | Mixed–Neutral | 0.012 | 0.007 | 1.72 | .256 |
|  | Occipital | Antagonist–Neutral | 0.018 | 0.005 | 3.57 | .001** |
|  |  | Agonist–Neutral | 0.003 | 0.005 | 0.59 | 1.000 |
|  |  | Mixed–Neutral | 0.033 | 0.008 | 4.02 | < .001*** |
| **Histamine** | Frontal | Antagonist–Neutral | −0.052 | 0.008 | −6.94 | < .001*** |
|  |  | Mixed–Neutral | −0.008 | 0.006 | −1.37 | .342 |
|  | Central | Antagonist–Neutral | −0.028 | 0.009 | −3.12 | .004** |
|  |  | Mixed–Neutral | −0.017 | 0.007 | −2.35 | .038* |
|  | Temporal | Antagonist–Neutral | −0.067 | 0.008 | −7.98 | < .001*** |
|  |  | Mixed–Neutral | 0.002 | 0.007 | 0.34 | 1.000 |
|  | Parietal | Antagonist–Neutral | −0.036 | 0.009 | −3.98 | < .001*** |
|  |  | Mixed–Neutral | −0.005 | 0.007 | −0.71 | .951 |
|  | Occipital | Antagonist–Neutral | −0.066 | 0.010 | −6.51 | < .001*** |
|  |  | Mixed–Neutral | −0.003 | 0.008 | −0.35 | 1.000 |
| **Acetylcholine** | Frontal | Antagonist–Neutral | 0.027 | 0.005 | 5.30 | < .001*** |
|  | Central | Antagonist–Neutral | 0.010 | 0.006 | 1.73 | .085 |
|  | Temporal | Antagonist–Neutral | 0.044 | 0.006 | 7.93 | < .001*** |
|  | Parietal | Antagonist–Neutral | 0.019 | 0.006 | 3.27 | .001** |
|  | Occipital | Antagonist–Neutral | 0.045 | 0.007 | 6.75 | < .001*** |

**p < .05; **p < .01; ***p < .001 (Bonferroni-corrected)*

**Supplementary Table S18. Simplified mixed-effects model. Estimated Marginal Means Contrasts for Alpha-Band Power by Neurotransmitter System and Electrode Location (mixed-effects model with patient-level random intercept only).**

| Neurotransmitter | Electrode | Contrast | Estimate | SE | t | p |
| --- | --- | --- | --- | --- | --- | --- |
| Dopamine | Frontal | Antagonist–Neutral | -0.001 | 0.002 | -0.57 | 1.000 |
|  |  | Agonist–Neutral | -0.006 | 0.002 | -2.87 | .012* |
|  |  | Mixed–Neutral | 0.006 | 0.002 | 2.36 | .055 |
|  | Central | Antagonist–Neutral | 0.002 | 0.003 | 0.79 | 1.000 |
|  |  | Agonist–Neutral | -0.005 | 0.002 | -2.32 | .061 |
|  |  | Mixed–Neutral | 0.007 | 0.003 | 2.52 | .036* |
|  | Temporal | Antagonist–Neutral | -0.010 | 0.003 | -3.80 | < .001*** |
|  |  | Agonist–Neutral | -0.007 | 0.002 | -3.25 | .003** |
|  |  | Mixed–Neutral | -0.001 | 0.003 | -0.50 | 1.000 |
|  | Parietal | Antagonist–Neutral | -0.002 | 0.003 | -0.86 | 1.000 |
|  |  | Agonist–Neutral | -0.004 | 0.002 | -1.66 | .291 |
|  |  | Mixed–Neutral | 0.005 | 0.003 | 1.59 | .337 |
|  | Occipital | Antagonist–Neutral | -0.016 | 0.003 | -5.16 | < .001*** |
|  |  | Agonist–Neutral | -0.002 | 0.002 | -0.68 | 1.000 |
|  |  | Mixed–Neutral | 0.005 | 0.003 | 1.47 | .422 |
| Serotonin | Frontal | Antagonist–Neutral | 0.018 | 0.004 | 4.56 | < .001*** |
|  |  | Agonist–Neutral | 0.003 | 0.001 | 2.30 | .064 |
|  |  | Mixed–Neutral | -0.001 | 0.002 | -0.42 | 1.000 |
|  | Central | Antagonist–Neutral | 0.015 | 0.005 | 3.15 | .005** |
|  |  | Agonist–Neutral | 0.008 | 0.002 | 4.92 | < .001*** |
|  |  | Mixed–Neutral | 0.003 | 0.003 | 0.87 | 1.000 |
|  | Temporal | Antagonist–Neutral | 0.007 | 0.004 | 1.61 | .320 |
|  |  | Agonist–Neutral | -0.008 | 0.002 | -5.04 | < .001*** |
|  |  | Mixed–Neutral | -0.004 | 0.003 | -1.50 | .400 |
|  | Parietal | Antagonist–Neutral | 0.011 | 0.005 | 2.38 | .051 |
|  |  | Agonist–Neutral | 0.001 | 0.002 | 0.51 | 1.000 |
|  |  | Mixed–Neutral | 0.003 | 0.003 | 1.20 | .685 |
|  | Occipital | Antagonist–Neutral | 0.000 | 0.005 | 0.03 | 1.000 |
|  |  | Agonist–Neutral | -0.024 | 0.002 | -13.28 | < .001*** |
|  |  | Mixed–Neutral | -0.004 | 0.003 | -1.32 | .559 |
| Norepinephrine | Frontal | Antagonist–Neutral | 0.003 | 0.003 | 1.28 | .603 |
|  |  | Agonist–Neutral | -0.006 | 0.002 | -2.54 | .033* |
|  |  | Mixed–Neutral | 0.010 | 0.004 | 2.59 | .029* |
|  | Central | Antagonist–Neutral | 0.007 | 0.003 | 2.27 | .070 |
|  |  | Agonist–Neutral | -0.006 | 0.003 | -2.41 | .048* |
|  |  | Mixed–Neutral | 0.005 | 0.005 | 1.03 | .906 |
|  | Temporal | Antagonist–Neutral | -0.010 | 0.003 | -3.66 | .001** |
|  |  | Agonist–Neutral | -0.002 | 0.003 | -0.64 | 1.000 |
|  |  | Mixed–Neutral | 0.003 | 0.004 | 0.68 | 1.000 |
|  | Parietal | Antagonist–Neutral | -0.001 | 0.003 | -0.30 | 1.000 |
|  |  | Agonist–Neutral | -0.006 | 0.003 | -2.32 | .061 |
|  |  | Mixed–Neutral | -0.002 | 0.005 | -0.36 | 1.000 |
|  | Occipital | Antagonist–Neutral | -0.028 | 0.003 | -8.36 | < .001*** |
|  |  | Agonist–Neutral | -0.007 | 0.003 | -2.28 | .067 |
|  |  | Mixed–Neutral | -0.017 | 0.005 | -3.22 | .004** |
| Histamine | Frontal | Antagonist–Neutral | -0.026 | 0.005 | -5.26 | < .001*** |
|  |  | Mixed–Neutral | -0.012 | 0.004 | -3.03 | .005** |
|  | Central | Antagonist–Neutral | -0.017 | 0.006 | -2.91 | .007** |
|  |  | Mixed–Neutral | -0.010 | 0.005 | -2.22 | .053 |
|  | Temporal | Antagonist–Neutral | -0.025 | 0.005 | -4.56 | < .001*** |
|  |  | Mixed–Neutral | -0.003 | 0.004 | -0.69 | .976 |
|  | Parietal | Antagonist–Neutral | -0.016 | 0.006 | -2.82 | .010** |
|  |  | Mixed–Neutral | -0.008 | 0.005 | -1.68 | .185 |
|  | Occipital | Antagonist–Neutral | -0.018 | 0.006 | -2.86 | .008** |
|  |  | Mixed–Neutral | -0.007 | 0.005 | -1.39 | .330 |
| Acetylcholine | Frontal | Antagonist–Neutral | 0.012 | 0.003 | 3.74 | < .001*** |
|  | Central | Antagonist–Neutral | 0.009 | 0.004 | 2.37 | .018* |
|  | Temporal | Antagonist–Neutral | 0.018 | 0.004 | 4.90 | < .001*** |
|  | Parietal | Antagonist–Neutral | 0.011 | 0.004 | 2.76 | .006** |
|  | Occipital | Antagonist–Neutral | 0.012 | 0.004 | 2.72 | .007** |

** p < .05; ** p < .01; *** p < .001 (Bonferroni-corrected)*

**Supplementary Table S19. Simplified mixed-effects model.** Estimated Marginal Means Contrasts for Beta 1-Band Power by Neurotransmitter System and Electrode Location (mixed-effects model with patient-level random intercept only).

| Neurotransmitter | Electrode | Contrast | Estimate | SE | t | p |
| --- | --- | --- | --- | --- | --- | --- |
| Dopamine | Frontal | Antagonist–Neutral | 0.002 | 0.001 | 1.99 | .140 |
|  |  | Agonist–Neutral | -0.002 | 0.001 | -1.68 | .280 |
|  |  | Mixed–Neutral | 0.002 | 0.001 | 1.58 | .342 |
|  | Central | Antagonist–Neutral | 0.003 | 0.002 | 1.88 | .182 |
|  |  | Agonist–Neutral | -0.002 | 0.001 | -1.64 | .306 |
|  |  | Mixed–Neutral | 0.002 | 0.002 | 1.24 | .642 |
|  | Temporal | Antagonist–Neutral | -0.000 | 0.001 | -0.09 | 1.000 |
|  |  | Agonist–Neutral | -0.003 | 0.001 | -2.66 | .023* |
|  |  | Mixed–Neutral | -0.001 | 0.002 | -0.38 | 1.000 |
|  | Parietal | Antagonist–Neutral | 0.001 | 0.002 | 0.96 | 1.000 |
|  |  | Agonist–Neutral | -0.002 | 0.001 | -1.93 | .160 |
|  |  | Mixed–Neutral | 0.001 | 0.002 | 0.57 | 1.000 |
|  | Occipital | Antagonist–Neutral | -0.001 | 0.002 | -0.31 | 1.000 |
|  |  | Agonist–Neutral | -0.002 | 0.001 | -1.69 | .273 |
|  |  | Mixed–Neutral | -0.000 | 0.002 | -0.05 | 1.000 |
| Serotonin | Frontal | Antagonist–Neutral | 0.002 | 0.002 | 1.20 | .694 |
|  |  | Agonist–Neutral | -0.000 | 0.001 | -0.25 | 1.000 |
|  |  | Mixed–Neutral | -0.001 | 0.001 | -0.53 | 1.000 |
|  | Central | Antagonist–Neutral | 0.001 | 0.003 | 0.49 | 1.000 |
|  |  | Agonist–Neutral | 0.001 | 0.001 | 0.78 | 1.000 |
|  |  | Mixed–Neutral | 0.001 | 0.002 | 0.45 | 1.000 |
|  | Temporal | Antagonist–Neutral | 0.001 | 0.002 | 0.33 | 1.000 |
|  |  | Agonist–Neutral | -0.001 | 0.001 | -0.98 | .981 |
|  |  | Mixed–Neutral | 0.000 | 0.001 | 0.02 | 1.000 |
|  | Parietal | Antagonist–Neutral | 0.001 | 0.003 | 0.36 | 1.000 |
|  |  | Agonist–Neutral | -0.000 | 0.001 | -0.11 | 1.000 |
|  |  | Mixed–Neutral | 0.000 | 0.002 | 0.25 | 1.000 |
|  | Occipital | Antagonist–Neutral | 0.002 | 0.003 | 0.54 | 1.000 |
|  |  | Agonist–Neutral | -0.002 | 0.001 | -2.27 | .070 |
|  |  | Mixed–Neutral | 0.000 | 0.002 | 0.21 | 1.000 |
| Norepinephrine | Frontal | Antagonist–Neutral | -0.001 | 0.001 | -0.67 | 1.000 |
|  |  | Agonist–Neutral | 0.001 | 0.001 | 0.85 | 1.000 |
|  |  | Mixed–Neutral | 0.001 | 0.002 | 0.64 | 1.000 |
|  | Central | Antagonist–Neutral | -0.000 | 0.002 | -0.09 | 1.000 |
|  |  | Agonist–Neutral | 0.000 | 0.001 | 0.02 | 1.000 |
|  |  | Mixed–Neutral | -0.000 | 0.003 | -0.01 | 1.000 |
|  | Temporal | Antagonist–Neutral | -0.003 | 0.002 | -1.77 | .229 |
|  |  | Agonist–Neutral | 0.002 | 0.001 | 1.74 | .246 |
|  |  | Mixed–Neutral | -0.000 | 0.002 | -0.20 | 1.000 |
|  | Parietal | Antagonist–Neutral | -0.000 | 0.002 | -0.25 | 1.000 |
|  |  | Agonist–Neutral | 0.001 | 0.001 | 0.97 | .995 |
|  |  | Mixed–Neutral | -0.000 | 0.003 | -0.12 | 1.000 |
|  | Occipital | Antagonist–Neutral | -0.004 | 0.002 | -2.11 | .104 |
|  |  | Agonist–Neutral | 0.002 | 0.002 | 0.95 | 1.000 |
|  |  | Mixed–Neutral | -0.002 | 0.003 | -0.75 | 1.000 |
| Histamine | Frontal | Antagonist–Neutral | -0.006 | 0.003 | -2.43 | .030* |
|  |  | Mixed–Neutral | -0.003 | 0.002 | -1.34 | .360 |
|  | Central | Antagonist–Neutral | -0.005 | 0.003 | -1.46 | .286 |
|  |  | Mixed–Neutral | -0.003 | 0.003 | -1.30 | .388 |
|  | Temporal | Antagonist–Neutral | -0.004 | 0.003 | -1.49 | .273 |
|  |  | Mixed–Neutral | 0.000 | 0.002 | 0.19 | 1.000 |
|  | Parietal | Antagonist–Neutral | -0.004 | 0.003 | -1.22 | .446 |
|  |  | Mixed–Neutral | -0.002 | 0.003 | -0.90 | .735 |
|  | Occipital | Antagonist–Neutral | -0.005 | 0.004 | -1.28 | .399 |
|  |  | Mixed–Neutral | -0.001 | 0.003 | -0.50 | 1.000 |
| Acetylcholine | Frontal | Antagonist–Neutral | 0.003 | 0.002 | 1.61 | .107 |
|  | Central | Antagonist–Neutral | 0.002 | 0.002 | 1.08 | .281 |
|  | Temporal | Antagonist–Neutral | 0.003 | 0.002 | 1.53 | .125 |
|  | Parietal | Antagonist–Neutral | 0.002 | 0.002 | 1.08 | .278 |
|  | Occipital | Antagonist–Neutral | 0.003 | 0.002 | 1.36 | .175 |

** p < .05; ** p < .01; *** p < .001 (Bonferroni-corrected)*

**Supplementary Table S20. Simplified mixed-effects model**. Estimated Marginal Means Contrasts for Beta 2-Band Power by Neurotransmitter System and Electrode Location (mixed-effects model with patient-level random intercept only).

| Neurotransmitter | Electrode | Contrast | Estimate | SE | t | p |
| --- | --- | --- | --- | --- | --- | --- |
| Dopamine | Frontal | Antagonist–Neutral | 0.002 | 0.001 | 2.70 | .021* |
|  |  | Agonist–Neutral | -0.001 | 0.001 | -0.89 | 1.000 |
|  |  | Mixed–Neutral | 0.001 | 0.001 | 0.89 | 1.000 |
|  | Central | Antagonist–Neutral | 0.002 | 0.001 | 2.24 | .075 |
|  |  | Agonist–Neutral | -0.001 | 0.001 | -1.80 | .217 |
|  |  | Mixed–Neutral | 0.000 | 0.001 | 0.51 | 1.000 |
|  | Temporal | Antagonist–Neutral | 0.001 | 0.001 | 1.01 | .939 |
|  |  | Agonist–Neutral | -0.001 | 0.001 | -2.03 | .127 |
|  |  | Mixed–Neutral | -0.001 | 0.001 | -0.71 | 1.000 |
|  | Parietal | Antagonist–Neutral | 0.002 | 0.001 | 1.87 | .183 |
|  |  | Agonist–Neutral | -0.001 | 0.001 | -1.69 | .275 |
|  |  | Mixed–Neutral | 0.000 | 0.001 | 0.39 | 1.000 |
|  | Occipital | Antagonist–Neutral | 0.001 | 0.001 | 0.86 | 1.000 |
|  |  | Agonist–Neutral | -0.001 | 0.001 | -1.29 | .591 |
|  |  | Mixed–Neutral | -0.000 | 0.001 | -0.09 | 1.000 |
| Serotonin | Frontal | Antagonist–Neutral | -0.000 | 0.001 | -0.39 | 1.000 |
|  |  | Agonist–Neutral | -0.000 | 0.000 | -0.71 | 1.000 |
|  |  | Mixed–Neutral | 0.000 | 0.001 | 0.21 | 1.000 |
|  | Central | Antagonist–Neutral | -0.000 | 0.001 | -0.12 | 1.000 |
|  |  | Agonist–Neutral | 0.000 | 0.001 | 0.06 | 1.000 |
|  |  | Mixed–Neutral | 0.001 | 0.001 | 1.08 | .848 |
|  | Temporal | Antagonist–Neutral | -0.001 | 0.001 | -0.50 | 1.000 |
|  |  | Agonist–Neutral | 0.000 | 0.000 | 0.00 | 1.000 |
|  |  | Mixed–Neutral | 0.000 | 0.001 | 0.48 | 1.000 |
|  | Parietal | Antagonist–Neutral | -0.000 | 0.001 | -0.30 | 1.000 |
|  |  | Agonist–Neutral | -0.000 | 0.000 | -0.80 | 1.000 |
|  |  | Mixed–Neutral | 0.001 | 0.001 | 0.71 | 1.000 |
|  | Occipital | Antagonist–Neutral | -0.000 | 0.002 | -0.30 | 1.000 |
|  |  | Agonist–Neutral | -0.000 | 0.001 | -0.73 | 1.000 |
|  |  | Mixed–Neutral | 0.001 | 0.001 | 0.69 | 1.000 |
| Norepinephrine | Frontal | Antagonist–Neutral | -0.001 | 0.001 | -1.43 | .461 |
|  |  | Agonist–Neutral | 0.001 | 0.001 | 0.96 | 1.000 |
|  |  | Mixed–Neutral | -0.001 | 0.001 | -0.56 | 1.000 |
|  | Central | Antagonist–Neutral | -0.001 | 0.001 | -0.56 | 1.000 |
|  |  | Agonist–Neutral | 0.001 | 0.001 | 0.67 | 1.000 |
|  |  | Mixed–Neutral | -0.001 | 0.001 | -0.60 | 1.000 |
|  | Temporal | Antagonist–Neutral | -0.001 | 0.001 | -1.33 | .548 |
|  |  | Agonist–Neutral | 0.001 | 0.001 | 1.53 | .375 |
|  |  | Mixed–Neutral | -0.001 | 0.001 | -0.63 | 1.000 |
|  | Parietal | Antagonist–Neutral | -0.001 | 0.001 | -0.77 | 1.000 |
|  |  | Agonist–Neutral | 0.001 | 0.001 | 1.35 | .535 |
|  |  | Mixed–Neutral | -0.001 | 0.001 | -0.74 | 1.000 |
|  | Occipital | Antagonist–Neutral | -0.002 | 0.001 | -1.62 | .318 |
|  |  | Agonist–Neutral | 0.000 | 0.001 | 0.46 | 1.000 |
|  |  | Mixed–Neutral | -0.002 | 0.002 | -1.09 | .832 |
| Histamine | Frontal | Antagonist–Neutral | -0.002 | 0.001 | -1.22 | .447 |
|  |  | Mixed–Neutral | -0.001 | 0.001 | -1.29 | .392 |
|  | Central | Antagonist–Neutral | -0.001 | 0.002 | -0.77 | .889 |
|  |  | Mixed–Neutral | -0.002 | 0.001 | -1.63 | .207 |
|  | Temporal | Antagonist–Neutral | -0.001 | 0.002 | -0.75 | .903 |
|  |  | Mixed–Neutral | 0.000 | 0.001 | 0.11 | 1.000 |
|  | Parietal | Antagonist–Neutral | -0.001 | 0.002 | -0.57 | 1.000 |
|  |  | Mixed–Neutral | -0.002 | 0.001 | -1.25 | .423 |
|  | Occipital | Antagonist–Neutral | -0.001 | 0.002 | -0.51 | 1.000 |
|  |  | Mixed–Neutral | -0.001 | 0.002 | -0.59 | 1.000 |
| Acetylcholine | Frontal | Antagonist–Neutral | 0.001 | 0.001 | 0.77 | .444 |
|  | Central | Antagonist–Neutral | 0.000 | 0.001 | 0.27 | .789 |
|  | Temporal | Antagonist–Neutral | 0.001 | 0.001 | 0.81 | .419 |
|  | Parietal | Antagonist–Neutral | 0.001 | 0.001 | 0.62 | .534 |
|  | Occipital | Antagonist–Neutral | 0.001 | 0.001 | 0.64 | .522 |

** p < .05; ** p < .01; *** p < .001 (Bonferroni-corrected)*

**Supplementary Table S21. Simplified mixed-effects model.** Estimated Marginal Means Contrasts for Gamma-Band Power by Neurotransmitter System and Electrode Location (mixed-effects model with patient-level random intercept only).

| Neurotransmitter | Electrode | Contrast | Estimate | SE | t | p |
| --- | --- | --- | --- | --- | --- | --- |
| Dopamine | Frontal | Antagonist–Neutral | 0.005 | 0.002 | 2.99 | .008** |
|  |  | Agonist–Neutral | 0.005 | 0.001 | 4.28 | .000*** |
|  |  | Mixed–Neutral | -0.001 | 0.002 | -0.61 | 1.000 |
|  | Central | Antagonist–Neutral | 0.002 | 0.002 | 0.81 | 1.000 |
|  |  | Agonist–Neutral | 0.002 | 0.002 | 1.01 | .944 |
|  |  | Mixed–Neutral | 0.000 | 0.002 | 0.04 | 1.000 |
|  | Temporal | Antagonist–Neutral | -0.000 | 0.002 | -0.05 | 1.000 |
|  |  | Agonist–Neutral | 0.001 | 0.001 | 0.51 | 1.000 |
|  |  | Mixed–Neutral | -0.002 | 0.002 | -1.31 | .568 |
|  | Parietal | Antagonist–Neutral | 0.001 | 0.002 | 0.38 | 1.000 |
|  |  | Agonist–Neutral | -0.001 | 0.002 | -0.70 | 1.000 |
|  |  | Mixed–Neutral | -0.003 | 0.002 | -1.64 | .305 |
|  | Occipital | Antagonist–Neutral | 0.003 | 0.002 | 1.49 | .409 |
|  |  | Agonist–Neutral | 0.003 | 0.002 | 1.70 | .270 |
|  |  | Mixed–Neutral | -0.000 | 0.002 | -0.14 | 1.000 |
| Serotonin | Frontal | Antagonist–Neutral | 0.006 | 0.003 | 2.17 | .091 |
|  |  | Agonist–Neutral | 0.003 | 0.001 | 3.75 | .001*** |
|  |  | Mixed–Neutral | -0.002 | 0.002 | -1.20 | .692 |
|  | Central | Antagonist–Neutral | -0.005 | 0.003 | -1.47 | .427 |
|  |  | Agonist–Neutral | 0.001 | 0.001 | 1.19 | .700 |
|  |  | Mixed–Neutral | -0.004 | 0.002 | -2.27 | .070 |
|  | Temporal | Antagonist–Neutral | -0.006 | 0.003 | -1.95 | .155 |
|  |  | Agonist–Neutral | 0.002 | 0.001 | 1.62 | .317 |
|  |  | Mixed–Neutral | -0.003 | 0.002 | -1.60 | .332 |
|  | Parietal | Antagonist–Neutral | 0.001 | 0.003 | 0.29 | 1.000 |
|  |  | Agonist–Neutral | 0.002 | 0.001 | 1.99 | .139 |
|  |  | Mixed–Neutral | -0.001 | 0.002 | -0.42 | 1.000 |
|  | Occipital | Antagonist–Neutral | -0.003 | 0.004 | -0.82 | 1.000 |
|  |  | Agonist–Neutral | 0.005 | 0.001 | 3.79 | .001*** |
|  |  | Mixed–Neutral | -0.001 | 0.002 | -0.52 | 1.000 |
| Norepinephrine | Central | Antagonist–Neutral | 0.007 | 0.002 | 3.24 | .004** |
|  |  | Agonist–Neutral | 0.002 | 0.002 | 1.32 | .564 |
|  |  | Mixed–Neutral | 0.007 | 0.003 | 2.08 | .112 |
|  | Frontal | Antagonist–Neutral | 0.005 | 0.002 | 2.94 | .010* |
|  |  | Agonist–Neutral | 0.002 | 0.001 | 1.12 | .787 |
|  |  | Mixed–Neutral | 0.005 | 0.003 | 2.07 | .116 |
|  | Temporal | Antagonist–Neutral | 0.004 | 0.002 | 2.37 | .053 |
|  |  | Agonist–Neutral | -0.001 | 0.002 | -0.36 | 1.000 |
|  |  | Mixed–Neutral | 0.005 | 0.003 | 1.75 | .241 |
|  | Parietal | Antagonist–Neutral | 0.007 | 0.002 | 3.58 | .001*** |
|  |  | Agonist–Neutral | 0.001 | 0.002 | 0.84 | 1.000 |
|  |  | Mixed–Neutral | 0.008 | 0.003 | 2.47 | .040* |
|  | Occipital | Antagonist–Neutral | 0.002 | 0.002 | 0.94 | 1.000 |
|  |  | Agonist–Neutral | -0.003 | 0.002 | -1.52 | .383 |
|  |  | Mixed–Neutral | 0.005 | 0.004 | 1.39 | .498 |
| Histamine | Frontal | Antagonist–Neutral | -0.006 | 0.003 | -1.73 | .169 |
|  |  | Mixed–Neutral | -0.001 | 0.003 | -0.51 | 1.000 |
|  | Central | Antagonist–Neutral | -0.002 | 0.004 | -0.48 | 1.000 |
|  |  | Mixed–Neutral | 0.000 | 0.003 | 0.06 | 1.000 |
|  | Temporal | Antagonist–Neutral | 0.001 | 0.004 | 0.19 | 1.000 |
|  |  | Mixed–Neutral | 0.002 | 0.003 | 0.79 | .858 |
|  | Parietal | Antagonist–Neutral | -0.006 | 0.004 | -1.39 | .327 |
|  |  | Mixed–Neutral | -0.001 | 0.003 | -0.29 | 1.000 |
|  | Occipital | Antagonist–Neutral | -0.004 | 0.005 | -0.92 | .714 |
|  |  | Mixed–Neutral | -0.001 | 0.004 | -0.38 | 1.000 |
| Acetylcholine | Frontal | Antagonist–Neutral | 0.005 | 0.002 | 2.26 | .024* |
|  | Central | Antagonist–Neutral | 0.001 | 0.003 | 0.41 | .686 |
|  | Temporal | Antagonist–Neutral | -0.002 | 0.002 | -0.63 | .530 |
|  | Parietal | Antagonist–Neutral | 0.002 | 0.003 | 0.79 | .429 |
|  | Occipital | Antagonist–Neutral | 0.004 | 0.003 | 1.25 | .213 |

** p < .05; ** p < .01; *** p < .001 (Bonferroni-corrected)*
